# Exploring the cellular and molecular basis of murine cardiac development through spatiotemporal transcriptome sequencing

**DOI:** 10.1093/gigascience/giaf012

**Published:** 2025-02-17

**Authors:** Jingmin Kang, Qingsong Li, Jie Liu, Lin Du, Peng Liu, Fuyan Liu, Yue Wang, Xunan Shen, Xujiao Luo, Ninghe Wang, Renhua Wu, Lei Song, Jizheng Wang, Xin Liu

**Affiliations:** BGI Research, Beijing 102601, China; BGI Research, Shenzhen 518083, China; BGI Research, Beijing 102601, China; BGI Research, Shenzhen 518083, China; Cardiomyopathy Ward, Fuwai Hospital, National Center for Cardiovascular Disease, Chinese Academy of Medical Science and Peking Union Medical College, Beijing 100037, China; BGI Research, Beijing 102601, China; College of Life Sciences, University of Chinese Academy of Sciences, Beijing 100049, China; BGI Research, Beijing 102601, China; BGI Research, Beijing 102601, China; BGI Research, Shenzhen 518083, China; BGI Research, Beijing 102601, China; BGI Research, Shenzhen 518083, China; State Key Laboratory of Quality Research in Chinese Medicine and Institute of Chinese Medical Sciences, University of Macau, Macao 999078, China; BGI Research, Beijing 102601, China; BGI Research, Shenzhen 518083, China; BGI Research, Beijing 102601, China; Clin Lab, BGI Genomics, Tianjin 300308, China; Clin Lab, BGI Genomics, Tianjin 300308, China; Cardiomyopathy Ward, Fuwai Hospital, National Center for Cardiovascular Disease , Chinese Academy of Medical Science and Peking Union Medical College, Beijing 100037, China; State Key Laboratory of Cardiovascular Disease, Fuwai Hospital, National Center for Cardiovascular Diseases, Chinese Academy of Medical Sciences and Peking Union Medical College, Beijing 100037, China; National Clinical Research Center of Cardiovascular Diseases, Fuwai Hospital, National Center for Cardiovascular Diseases, Chinese Academy of Medical Sciences and Peking Union Medical College, Beijing 100037, China; State Key Laboratory of Cardiovascular Disease, Fuwai Hospital, National Center for Cardiovascular Diseases, Chinese Academy of Medical Sciences and Peking Union Medical College, Beijing 100037, China; BGI Research, Beijing 102601, China; BGI Research, Shenzhen 518083, China

**Keywords:** spatial transcriptomics, mouse heart development, myocardial regeneration ability, left and right atria

## Abstract

**Background:**

Spatial transcriptomics is a powerful tool that integrates molecular data with spatial information, thereby facilitating a deeper comprehension of tissue morphology and cellular interactions. In our study, we utilized cutting-edge spatial transcriptome sequencing technology to explore the development of the mouse heart and construct a comprehensive spatiotemporal cell atlas of early murine cardiac development.

**Results:**

Through the analysis of this atlas, we elucidated the spatial organization of cardiac cellular lineages and their interactions during the developmental process. Notably, we observed dynamic changes in gene expression within fibroblasts and cardiomyocytes. Moreover, we identified critical genes, such as *Igf2, H19*, and *Tcap*, as well as transcription factors *Tcf12* and *Plagl1*, which may be associated with the loss of myocardial regeneration ability during early heart development. In addition, we successfully identified marker genes, like *Adamts8* and *Bmp10*, that can distinguish between the left and right atria.

**Conclusion:**

Our study provides novel insights into murine cardiac development and offers a valuable resource for future investigations in the field of heart research, highlighting the significance of spatial transcriptomics in understanding the complex processes of organ development.

## Background

Single-cell sequencing has emerged as a crucial tool for investigating the cellular and molecular aspects of heart and cardiac diseases. This approach enables the identification of previously unknown cell types, as well as the characterization of gene expression patterns and regulatory networks at single-cell level [1–4]. Through the use of single-cell sequencing, researchers have made significant progress in understanding various cardiac diseases, including heart failure [5, 6], arrhythmia [7], and cardiomyopathy [8, 9]. These advancements have provided novel insights into disease mechanisms and potential therapeutic targets. However, despite these achievements, there is still a lack of comprehensive understanding regarding the cellular and molecular features of the heart during cardiac development and diseases. This knowledge gap is primarily due to technical limitations inherent in single-cell sequencing, especially the absence of spatial information and biases associated with different cell types [10]. Therefore, it is crucial to explore the spatial organization of heart cells and utilize this spatial information to comprehensively investigate the cellular basis of cardiac development and diseases.

Spatial transcriptomics is a rapidly advancing field that aims to integrate spatial information with transcriptomic data, allowing for the investigation of tissue organization and cellular interactions [11]. Recent advancements in spatial transcriptome technologies, such as *in situ* sequencing [12] and capture-based spatial transcriptome sequencing [13], have revolutionized the simultaneous visualization and quantification of gene expression *in situ*, eliminating the need for tissue dissociation. These innovative approaches have proven successful in studying various biological systems, including the brain [14], developing embryos [13], and disease contexts such as cancer [15] and cardiovascular disease. In the field of cardiac research, spatial transcriptomics has been employed to explore cardiac development in chickens [16], providing novel insights into the interplay between cellular differentiation and morphogenesis that underlies heart function and pathology. To comprehensively study organs like the heart, the application of spatial transcriptome technologies should be further expanded, which will enable a more comprehensive understanding of the spatial organization of cells within the heart and its implications for cardiac function and disease.

Studies on the mouse heart have demonstrated its regenerative capacity during the neonatal stage, which diminishes as it matures [17]. To gain a deeper understanding of the mechanisms underlying neonatal heart regeneration and explore possible strategies to enhance regeneration, various approaches, including genetic manipulation, tissue engineering, and stem cell therapy, have been investigated. However, despite these efforts, a comprehensive understanding of these mechanisms is still lacking, highlighting the need for further research. In this study, we employed SpaTial Enhanced REsolution Omics-sequencing (Stereo-seq) [13] to construct a spatiotemporal cell atlas of developing mouse hearts. This atlas encompasses spatial transcriptome data from both regeneration-capable neonatal hearts and relatively mature hearts. By analyzing the dynamics of cellular and gene expression during heart development, we identified specific genes that may be associated with the loss of regeneration ability. Our findings provide new insights into murine cardiac development and serve as a valuable resource for future investigations in the field of heart research.

## Results

### Constructing a spatiotemporal atlas of mouse heart during early development

**Figure 1:**
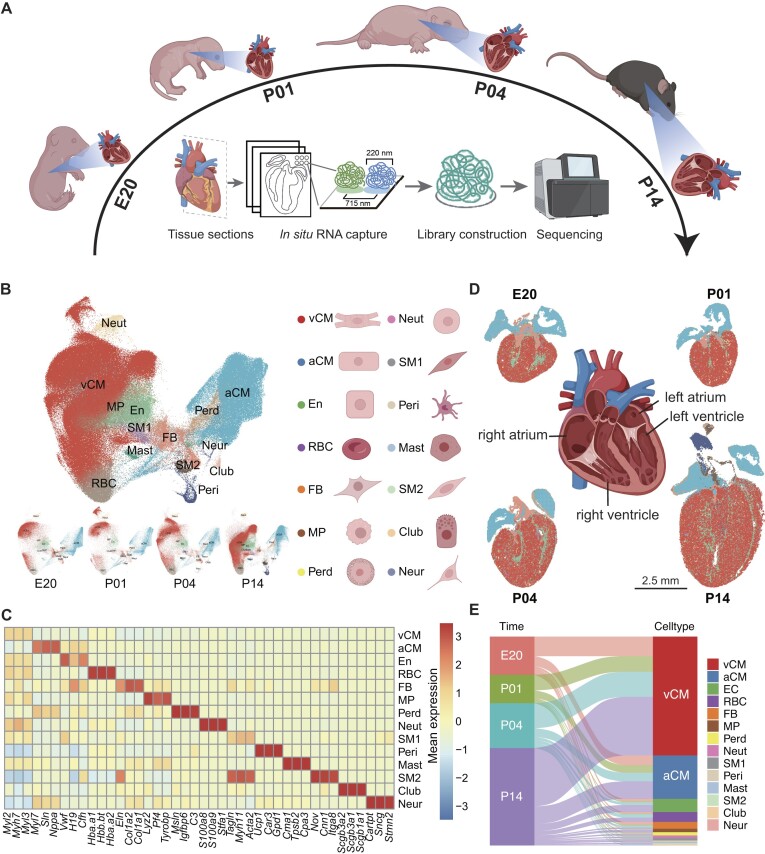
Spatial and temporal atlas of mouse heart development. (A) Mouse heart sampling and Stereo-seq protocol. (B) Uniform manifold approximation and projection (UMAP) of Stereo-seq clustering and annotation of 22 spatial mouse chips. (C) Heatmap of the expression of the top 3 marker genes for each cell type. (D) Spatial and temporal distribution of mouse heart cells. (E) Proportional changes of different cell types at 4 time points.

To create a comprehensive spatiotemporal transcriptomic atlas of early mouse heart development, we carefully selected 4 specific time points, including embryonic day 20 (E20), postnatal day 1 (P01), postnatal day 4 (P04), and postnatal day 14 (P14). Heart samples were obtained by freezing and embedding 2 mice at each time point. From each heart, 3 adjacent frozen sections were chosen from the middle region and subjected to spatial transcriptome sequencing using the Stereo-seq technique (Fig. 1A). In total, we obtained 6 spatiotemporal sections with 3 technical replicates for each time point, resulting in 22 high-quality sections after excluding any low-quality data. This dataset, consisting of high-quality sections, represents a substantial resource for early mouse heart development (Supplementary Fig. S1a). We then performed cell segmentation to obtain cell bins, based on a bin size of approximately 25 μm (bin 50), resulting in 330,857 cell bins in total (Supplementary Table S1). For these obtained cell bins, we performed gene expression–based cell clustering and utilized known marker genes to annotate the cell types. This analysis revealed the presence of 14 distinct cell types (Fig. 1B, Supplementary Fig. S1b, and Supplementary Table S2). By identifying differentially expressed genes, we further determined marker genes for each cell type (Fig. 1C). To generate a single-cell transcriptomic atlas with spatial information, we mapped the annotated cell types to their respective spatial positions (Fig. 1D). This atlas provides a visual representation of the location of each cell and the expression levels of individual genes *in situ*. Overall, we successfully constructed a comprehensive spatiotemporal transcriptomic atlas of mouse heart development.

Leveraging this constructed atlas, we conducted a detailed investigation into the cellular composition of the heart during early development. Our analysis revealed that cardiomyocytes were the predominant cell type. Cardiomyocytes constitute the primary cell type, wherein atrial cardiomyocytes (aCMs) account for approximately 21% of the total cell population, and ventricular cardiomyocytes (vCMs) represent approximately 57% of the overall cell proportion (Fig. 1E). In terms of developmental dynamics, we observed an increase in the proportion of vCMs over time, accompanied by a decrease in the proportion of aCMs (Fig. 1E). This suggests a major shift in the cellular composition of the heart during early development. Additionally, we identified a distinct population of fibroblasts (FBs) located within the ventricle and atrium, characterized by the expression of collagen family genes, including *Fbln5*. Interestingly, the number of these fibroblasts decreased as heart development progressed (Fig. 1E). Furthermore, our analysis revealed a significant presence of endothelial cells, primarily situated in the middle region of the ventricle. These endothelial cells may play a crucial role in vascular development and maintenance within the heart. Lastly, we identified pericardial cells (Perd) located on the surface of the heart, which exhibited notable expression of *C3, Igfbp6*, and *Msln* genes (Supplementary Fig. S4a). These pericardial cells likely contribute to the structural integrity and protection of the heart. Collectively, our spatiotemporal transcriptomic atlas of heart development provides a comprehensive understanding of the cellular composition within the heart.

### Cardiomyocytes mediated cell–cell interaction weakening during heart development

**Figure 2:**
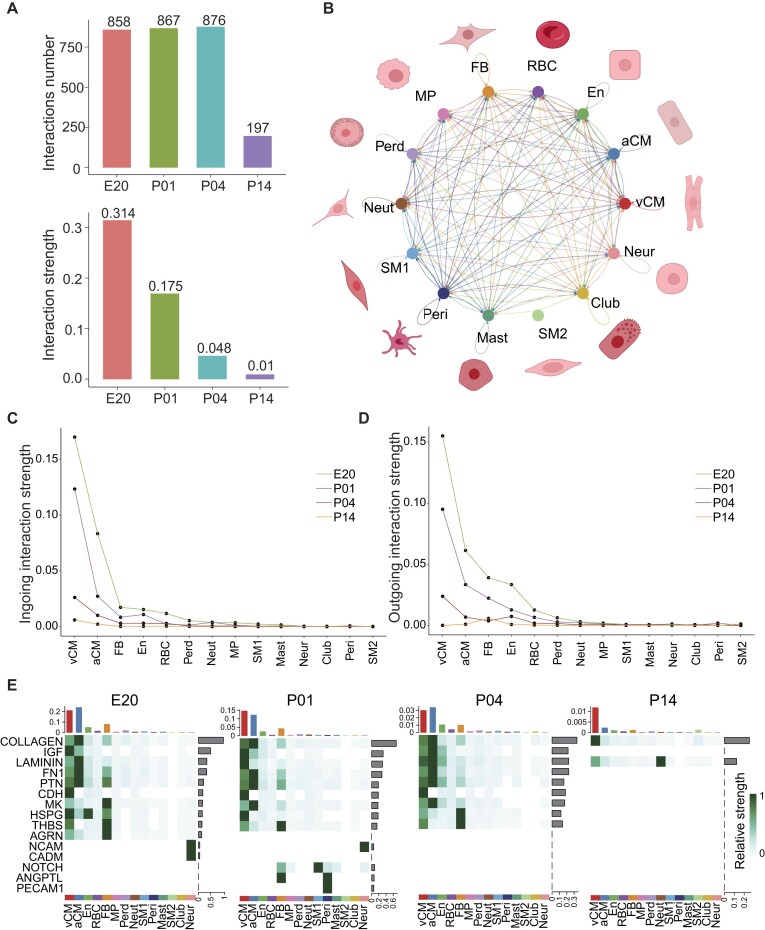
Cell–cell interactions of different cell types. (A) Cell interaction strength and interaction count at 4 time points. (B) Cell interaction strength among cells at E20. The direction of the arrow indicates the direction of the signal, and the thickness of the line segment indicates the signal strength. (C) Ingoing interaction strength of different cell types at 4 time points. (D) Outgoing interaction strength of different cell types at 4 time points. (E) Signal pathway strength of different cell types at 4 time points. The shade represents the relative strength of the interaction between the 2 cell types.

Cell–cell interactions play a vital role in ensuring the proper functioning of biological systems by facilitating coordination and communication among cells [18, 19]. These interactions are crucial for various biological processes, including development. In addition to analyzing the cellular composition using our spatiotemporal atlas of heart development, we delved into the investigation of cell–cell interactions (Supplementary Table S3), particularly focusing on how these interactions change throughout development. Initially, we calculated the overall number and strength of cell interactions at different time points (Fig. 2A). The result reflected that the number of cell–cell interactions did not significantly decrease until P14, while the strength of interactions gradually diminished over time. This suggests that during early development, the factors involved in cell–cell interactions remain intact, but the interactions weaken as development progresses. Further analysis of the pathways of interaction utilized by cells (Supplementary Fig. S2a) unveiled distinct patterns of activity at different time points. As time passes, the signals related to the construction of the cell skeleton, such as fibronectin 1, laminin, and collagen [20], gradually weaken. Along with that, signals related to insulin-like growth factor cell growth [21] also gradually weaken and disappear after 14 days. This suggests that there is a certain correlation between cell growth and the construction of the cell skeleton. As the cell skeleton is completed, the cell morphology gradually becomes fixed, and probably the cells lose their regenerative ability.

In addition to examining the overall cell–cell interactions at the 4 stages of development, we conducted a detailed investigation into the interactions between different cell types (Fig. 2B and Supplementary Fig. S3a–d). Our analysis revealed that the interactions between aCMs, vCMs, and FBs exhibited the highest strength. Further exploration of the input and output interactions of each cell type (Fig. 2C, D) unveiled that vCMs predominantly acted as the major signal-receiving cell type, while aCMs served as the primary signal-output cell type, particularly during the relatively early stages (E20, P01, and P04). However, at P14, in addition to the overall weakening of interactions, we observed a substantial reduction in the output strength of aCMs, which was previously the main output cell type, despite vCMs still receiving strong signals. This substantial reduction in interaction from aCMs likely contributed significantly to the overall decrease and weakening of cell–cell interactions during cardiac maturation. Thus, we further analyzed the interaction factors present in different cell types at each of the 4 stages (Fig. 2E) to elucidate the changes in cell interaction factors within aCMs. Our findings indicated that the stage-specific interaction factors of the neural cell adhesion molecule (NCAM) and cell adhesion molecule (CADM) at E20 were primarily present in neurons. Similarly, the stage-specific interaction factors Notch receptor (NOTCH), angiopoietin-like protein, and platelet endothelial cell adhesion molecule 1 at P01 were mainly observed in FBs and pericytes. At P14, particularly in aCMs and vCMs, which were previously active in interactions, all cell interaction factors, except for collagen, were scarcely present. These results further support the notion that the decrease in cell–cell interactions during cardiac maturation can be attributed to the absence of these interaction factors. In summary, leveraging the spatiotemporal atlas of heart development, we conducted an extensive investigation into cell–cell interactions during cardiac development and identified cell-type-specific interaction factors. Notably, we discovered that a key characteristic of mouse cardiac maturation is the decrease in cell–cell interactions among FBs, aCMs, and vCMs.

### Investigating detailed cell subtypes in mouse heart and their dynamics

**Figure 3:**
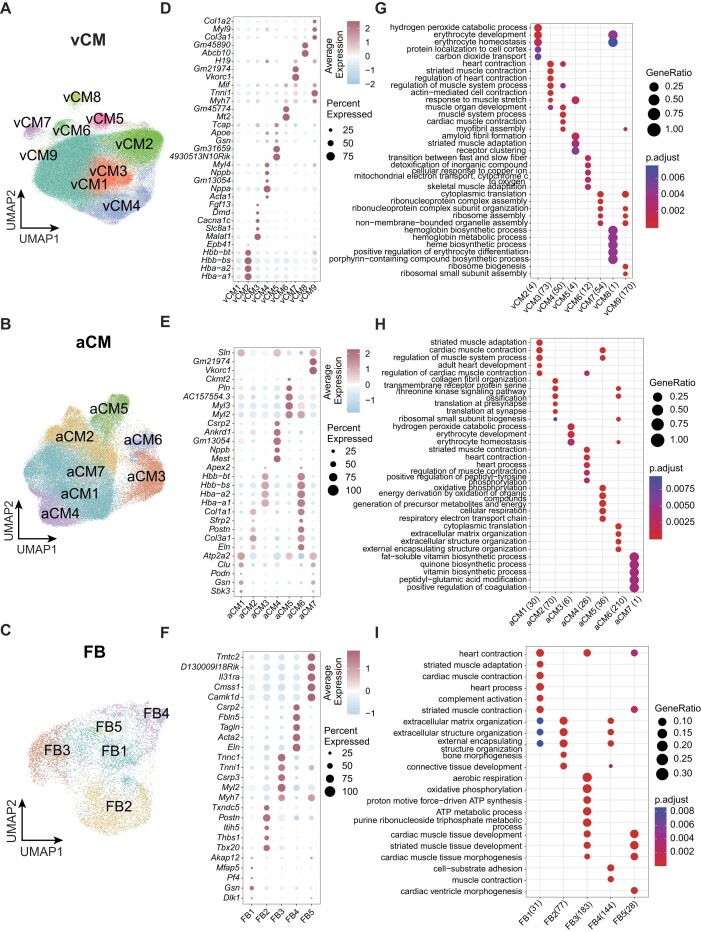
Classification of cell subtypes. (A–C) Subtype clustering of vCMs, aCMs, and FBs. (D–F) Expression of the top 5 marker genes in vCMs, aCMs, and FBs. (G–I) Enrichment of the top 5 GO functions in vCMs, aCMs, and FBs.

The spatiotemporal atlas provides a valuable subcellular-level transcriptomic dataset, offering spatial positional gene expression information about the heart. This resource enables us to delve into finer cell classification and characterization. Therefore, focusing on the 3 most abundant and prominent cell types—namely, vCMs, aCMs, and FBs—we conducted a comprehensive cell-type annotation. By analyzing their gene expression characteristics, we successfully annotated vCMs, aCMs, and FBs into 9, 7, and 5 distinct cell subtypes, respectively (Fig. 3A–C). To gain further insights into these annotated subtypes, we examined their gene expression profiles and identified subtype-specific genes (Fig. 3D–F). Leveraging these genes that exhibited specific expression patterns within each subtype, we conducted gene function enrichment analysis (Fig. 3G–I). This analysis allowed us to uncover the functional roles and pathways associated with each cell subtype.

Based on the above cell clustering results, we further mapped these subtype cells to their physical locations in the heart (Supplementary Fig. S4b–d). We observed that certain subtypes exhibited obviously distinct spatial distribution characteristics, which showed consistency with their possible functions. For example, cells from 1 subtype of vCMs (vCM4) were found to aggregate near the ventricular cavity (Supplementary Fig. S4 e), and specially expressed genes of these cells were enriched in functions related to muscle organ development, muscle system process, cardiac muscle contraction, myofibril assembly, and so on (Fig. 3G, Supplementary Table S4). Both their spatial distribution and functional enrichment suggest that vCM4 cells are involved in the contraction and relaxation activities of the heart. Meanwhile, cells from the other subtype of aCMs (aCM5) were observed to aggregate in the region of the tricuspid and bicuspid valves ( Supplementary Fig. S4f). Enrichment of their specially expressed genes indicated their possible functions related to oxidative phosphorylation, energy derivation by oxidation of organic compounds, generation of precursor metabolites and energy, cellular respiration, respiratory electron transport chain, and so on (Fig. 3h, Supplementary Table S5). We thus deduce that these cells may play a crucial role in providing energy for the opening and closing movements of the tricuspid and bicuspid valves, contributing to the regularity and effectiveness of cardiac pulsation.

Similarly, for fibroblasts, we found cells from 1 subtype (FB4) to aggregate in the region of the aorta (Supplementary Fig. S4g), with a higher proportion at E20, P01, and P04 and a lower proportion at P14. We found the specially expressed genes of these cells to be enriched in functions related to cell-substrate adhesion and cardiac ventricle morphogenesis (Fig. 3I, Supplementary Table S6). This suggests FB4 cells are associated with the construction of the cardiac cell scaffold, and this activity is more active in the early stages of heart development. While not all subtypes could be functionally annotated, this investigation allows us to gain a deeper understanding of the dynamic changes occurring within these cell subtypes throughout the developmental stages of the heart.

### Cell trajectories revealed critical genes involved in regeneration ability loss

**Figure 4:**
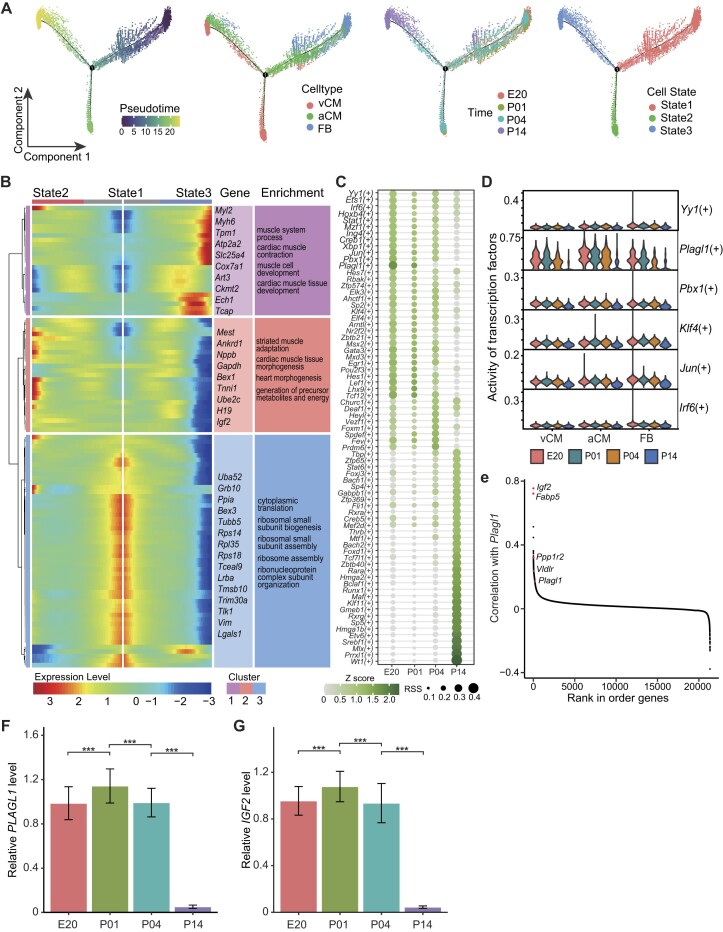
Trajectory analysis and transcription factor analysis. (A) Trajectory analysis of 3 cell types (aCMs, vCMs, FBs). The distribution of pseudo-time, cell types, time points, and cell states along the trajectory from left to right. (B) Heatmap of gene expression changes in 3 cell states at branch node 1 and GO enrichment results of different clustered genes. The left cluster tree divides genes into 3 groups according to the trend of gene expression. (C) Transcription factor activity changes among 3 cell types (aCMs, vCMs, FBs) at 4 time points. (D) Changes in the activity of transcription factors targeting the gene *Igf2* in 3 different cell types over time. (E) Correlation between *Plagl1* transcription factor activity and gene expression. The red dots represent the target genes of *Plagl1*. (F) Relative quantification of *Plagl1* at 4 time points using quantitative PCR. (G) Relative quantification of *Igf2* at 4 time points using quantitative PCR.

While human cardiac muscle cells lack regenerative capacity, mouse cardiac muscle cells possess the ability to regenerate during early development [22]. It is crucial to understand the mechanisms underlying the loss of regenerative ability in mouse cardiac muscle cells during the developmental process. This understanding will unravel the molecular intricacies of cardiac muscle regeneration and aid future research on important heart diseases. Our heart spatiotemporal atlas of the heart encompasses 4 time points during early embryonic development. The first 3 time points correspond to the regenerative period of the mouse heart, while the final time point (P14) signifies the loss of regenerative ability [23]. To investigate the cellular and molecular changes associated with cardiac muscle regeneration, we conducted an in-depth analysis by comparing the data from the final time point with the preceding 3 time points. Pseudotime analysis using Monocle2 was conducted on the subtypes of vCMs, aCMs, and FBs. Given that cardiomyocytes are deemed terminally differentiated cells, FBs were designated as the origin in the pseudotime trajectory (Fig. 4A and Supplementary Fig. S5a, b). This analysis revealed distinct temporal trends for these major cell types. At E20, aCMs and FBs exhibited high similarity compared to vCMs. As time progressed, all 3 major cell types underwent further differentiation, and by P14, the states of several cell types became consistent. The branching pattern observed among these subtypes (Fig. 4A) indicated clear differentiation during heart development. Moreover, genes can be clustered into 3 cell states based on their expression changes along the trajectory (Fig. 4B, Supplementary Table S7). During the development of the heart and maturation of cardiac muscle cells, we observed notable changes in gene expression levels that correlated with the loss of regenerative capacity and the acquisition of contractile ability and cardiac function. Specifically, the expression levels of *Igf2* (insulin like growth factor 2 gene) [24, 25], *H19* (a long noncoding RNA gene, *H19* imprinted maternally expressed transcript) [26, 27], and other genes associated with cell differentiation gradually decreased. Conversely, the expression levels of *Tcap* (titin-cap gene) [28], *Myh6* (myosin heavy chain 6 gene) [29], *Atp2a2* (ATPase sarcoplasmic, endoplasmic reticulum Ca^2+^ transporting 2 gene) [30], and other genes gradually increased, indicating the acquisition of contractile ability and the initiation of cardiac function. These changes in gene expression profiles provide insights into the molecular processes underlying the transition from regenerative capacity to contractile ability during cardiac muscle development.

Transcription factors (TFs) are pivotal in regulating gene expression during various biological processes [31]. Thus, we conducted a detailed investigation into the changes in transcription factors during cardiac development to identify TFs that may contribute to the loss of myocardial cell regeneration capacity (Supplementary Table S8). Based on the regulon specificity score (RSS, Fig. 4C), we categorized the identified TFs into 2 groups. The first group comprised TFs that exhibited higher activity levels in the early stages of cardiac development but gradually decreased in specificity as development progressed. In contrast, the second group consisted of TFs that displayed higher activity levels during the later stages of cardiac maturation. Among TFs of the second group, we identified several important TFs that have been previously reported to be associated with cardiac development. These include *Wt1* (Wilms’ tumor 1 gene), *Prrxl1* (paired related homeobox 1 gene), and *Srebf1* (sterol regulatory element binding transcription factor 1). For instance, *Wt1* contains 4 zinc finger motifs at the C-terminus, which are crucial for DNA binding and gene activation [32]. In the context of cardiac development, *Wt1* primarily regulates processes such as the epithelial-to-mesenchymal transition and angiogenesis, and it has been shown to play a critical role in early cardiac development [33]. Additionally, our analysis uncovered previously unreported TFs that may play a role in cardiac maturation, such as *Mlx* (max-like protein X gene), which warrants further investigation. On the other hand, the TFs in the first group are more likely to be associated with the loss of myocardial cell regeneration capacity during this process. Among these, we identified *Tcf12* (transcription factor 12) [34], *Plagl1* (pleomorphic adenoma gene-like 1), and other transcription factors that exhibited decreased activity levels with cardiac maturation.

In addition, as our analysis of differential gene expression identified *Igf2* to be possibly involved in heart development and regeneration ability loss, we further investigated whether TFs targeting the *Igf* genes exhibited expression changes associated with cardiac maturation. Specifically, in our analysis of fibroblasts and myocardial cells, we identified 6 transcription factors targeting *Igf2*, including the aforementioned *Plagl1*. Notably, we observed a sustained decrease in the transcription factor activity of *Plagl1* along fibroblasts and myocardial cells during cardiac development and maturation (Fig. 4D). Furthermore, when examining its TF activity correlation with the expression of other genes (Fig. 4E), we found that the TF *Plagl1* exhibited the strongest correlation with *Igf2*. We further validated the temporal expression changes of *Igf2* and *Plagl1* through quantitative PCR (qPCR) (Fig. 4F, G). We found that both genes exhibited a significant decrease in expression on postnatal day 14, which is consistent with our spatial data.

In addition, we investigated the characteristics of early mouse cardiac cells using spatial transcriptomics data from early mouse embryos [35]. First, we performed clustering on the spatial transcriptomics data of early embryos (E9.5, E10.5, E11.5, and E12.5) and identified clusters 8 and 16 as undifferentiated cardiac cells using marker genes specific to myocardial cells (Supplementary Fig. S6a–c). Furthermore, we identified the expression of key genes and transcription factors in early cardiac cells. *Igf2, H19, Tcf12*, and *Plagl1* were highly expressed, while *Wt1, Prrxl1*, and *Srebf1* were expressed at lower levels, consistent with the developmental changes observed in cardiac cells (Supplementary Fig. S6d).

This finding suggests that *Plagl1* may have been regulating *Igf2* during early heart development. These findings point to *Plagl1* as a potential TF involved in the differentiation and regeneration of myocardial cells. However, further comprehensive research is necessary to delve deeper into these findings and investigate the functional roles of the identified candidate genes.

### Identification of atrial asymmetry related genes

**Figure 5:**
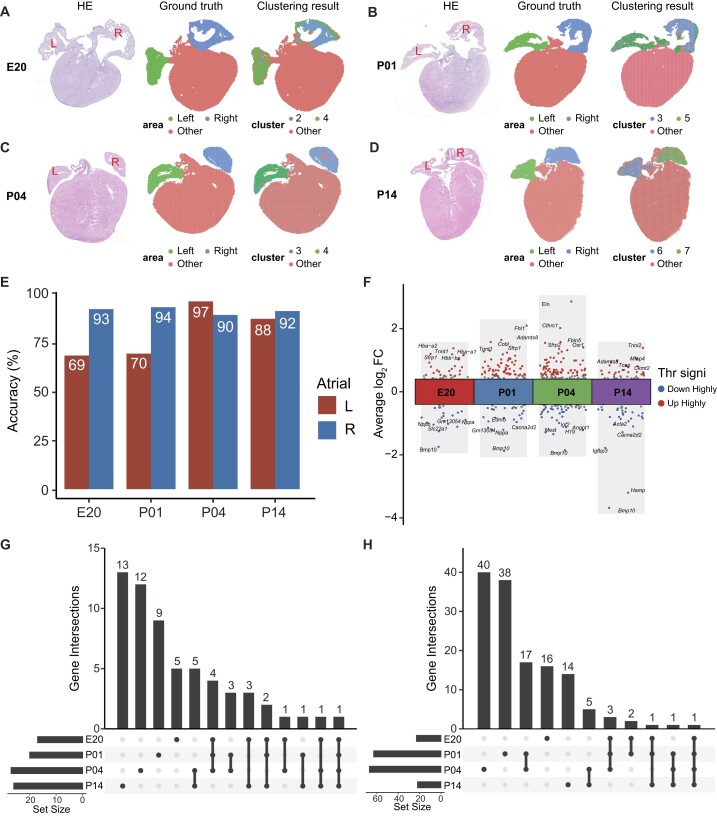
Identification of left and right atria. (A–D) The spatial representation of HE sections, the actual regions of the left and right atria, and the clustering results of mouse hearts at 4 time points. (E) Accuracy of clustering results of the left and right atria relative to the actual distribution. (F) Differential expression of genes comparing the left atrium to the right atrium at 4 time points. (G) Intersection of highly variable genes in the left atrium at 4 time points. (H) Intersection of highly variable genes in the right atrium at 4 time points.

By utilizing the spatiotemporal transcriptomic atlas, we can leverage the spatial information of gene expression for molecular-level investigations. The left–right asymmetry of the heart is crucial for its functional performance [36], making it essential to understand the development of the left and right ventricles and atria. To achieve this, we first divided the left and right atrial regions based on their positions for all the Stereo-seq sections. We then validated these divisions by performing hematoxylin and eosin (HE) staining on adjacent sections (Fig. 5A–D). This approach ensured the accuracy of our spatially defined regions. In contrast, when clustering and analyzing without incorporating spatial positional information, using only the uniform manifold approximation and projection (UMAP) plot of all cells from each chip, there would be a certain degree of error. We mapped the results of unsupervised clustering of the left and right atria performed solely based on the UMAP plots onto our spatial atlas (Fig. 5A–D, Supplementary Fig. S7a–d) and counted the number of cells predominantly distributed in the left or right atrium, calculating the percentage of these cells compared to the total cells in the left or right atrium. We found that the proportion of left atrial cells could be directly differentiated to be lower (69%, 70%, 97%, and 88%) compared to the right atrial cells (93%, 94%, 90%, and 92%) at the 4 time points (Fig. 5e, Supplementary Table S9). These findings highlight the importance of considering spatial information for accurate identification and analysis of left and right atrial cells.

Subsequently, following the successful distinction between left and right atrial cells, we proceeded to investigate the disparities in gene expression between these 2 regions. By comparing the cells of the left and right atria at 4 time points, we identified a set of genes that were differentially expressed (Fig. 5F). This set comprised genes that were upregulated in the left atrium (Fig. 5G) and genes that were upregulated in the right atrium (Fig. 5H) at the 4 designated time points. Specifically, in the left atrium, we observed 17, 20, 27, and 26 upregulated genes at E20, P01, P04, and P14, respectively. Conversely, the right atrium exhibited 23, 63, 67, and 22 upregulated genes at the corresponding time points. To gain further insights into the functional implications of these gene expression differences, we conducted a functional enrichment analysis of the identified genes (Supplementary Fig. S7e). Notably, we found that the upregulated genes in the right atrium were significantly enriched in Gene Ontology (GO) terms related to heart development. In contrast, the upregulated genes in the left atrium did not exhibit substantial enrichment in any heart development–related GO terms.

We then conducted an investigation into specific genes that may be associated with atrial asymmetry. Within one of the previously mentioned enriched GO terms (GO:0003228, atrial cardiac muscle tissue development), we identified 4 genes, including *Pitx2, Bmp10, Eng*, and *Adamts8. Pitx2*, a transcription factor known for its involvement in regulating left–right asymmetry development of the heart and other organs, has been established as a gene specific to the left atrium [37]. Our analysis revealed significant upregulation of *Pitx2* in the left atrium at the P01 and P04 time points (Supplementary Fig. S8a). *Bmp10*, known as a regulatory gene of *Pitx2*, exhibited specific expression in the right atrium throughout all stages (Supplementary Fig. S8b). Furthermore, *Eng* displayed significantly higher expression in the right atrium at the P04 stage (Supplementary Fig. S8c). In addition, we consistently observed high expression of *Adamts8* in the left atrium throughout the entire developmental stage (Supplementary Fig. S8d). These findings shed light on the potential involvement of these genes in atrial asymmetry and provide valuable insights into the molecular mechanisms underlying the development of the left and right atria. Further research is warranted to explore the functional roles of these genes and their contributions to atrial cardiac muscle tissue development.

## Discussion

The heart plays a crucial role in our physiology, and conducting single-cell and spatial transcriptomic studies on this organ can significantly enhance our understanding of cellular and molecular changes occurring during heart development and related diseases. Such studies provide essential insights into the molecular mechanisms underlying heart-related conditions and lay the groundwork for the identification of novel targets for the treatment of heart disease [1, 6, 10, 38, 39]. In our study, we constructed a comprehensive spatiotemporal transcriptomic atlas of early mouse heart development. We examined the changes occurring at the cellular and gene levels throughout the developmental process, including alterations in cell-type composition, intercellular communication, shifts in cardiac cell types during development, and the identification of candidate genes crucial for cardiac development. Our findings largely align with previous single-cell studies conducted in mice, validating the reliability of our spatiotemporal atlas. For instance, we identified the transcription factor *Wt1* as potentially playing a crucial role in mouse heart development, which has already been reported in a previous mouse single-cell study [39]. However, our spatiotemporal atlas allowed us to systematically describe the cellular and gene-level changes that occur during early heart development, providing a more comprehensive understanding of the process. Moreover, we identified novel genes that may be associated with heart development. Utilizing the spatial information available in our atlas, we compared the cell types present in the left and right atria and identified genes with specific expression patterns throughout development. This analysis deepened our understanding of heart asymmetry and shed light on the molecular underpinnings of this phenomenon. Notably, our study focused on the unique aspects of mouse heart development in the early stages, distinguishing it from previously published spatiotemporal atlases of chicken heart development [16]. We specifically explored cell-type changes and potential genes related to regeneration, which are distinctive to the early developmental stage of the mouse heart.

Cardiac regeneration in mice has been a prominent focus of heart research, with previous studies identifying molecules and genes associated with early cardiac regeneration in mice [40–43]. However, the mechanisms underlying myocardial cell regeneration specific to early mouse hearts, which are absent in later stages and other mammals, remain poorly understood. In our study, we conducted a comparative analysis of spatiotemporal transcriptomic data from early and late stages of mouse hearts, leading us to identify *Igf2* and the transcription factor *Plagl1*, which regulates *Igf2*, as potential contributors to early mouse cardiac regeneration. Previous studies have suggested the involvement of *Igf2* in the regeneration of the heart and other tissues in mice [44–46], while other studies, including a recent preprint article, have described the role of *Plagl1* in retinal regeneration in mice [47, 48]. Notably, *Plagl1* has been shown to have inhibitory effects on cell proliferation and act as a tumor suppressor in humans [49, 50]. Additionaly, *Plagl1* is an imprinted gene, exhibiting specific expression of the paternal allele in multiple tissues and being implicated in the pathogenesis of congenital heart disease [51]. Given that *Plagl1* regulates numerous downstream genes, the regulatory pathway from *Plagl1* to *Igf2* and its impact on early myocardial cell regeneration remain unclear. Our study highlights the significance of investigating the *Plagl1*–*Igf2* regulation pathway for further research on myocardial cell regeneration in mice and even humans, laying the foundation for subsequent mechanistic studies.

Our study constructed a spatiotemporal transcriptomic atlas of early mouse heart development and proposed cellular and molecular changes related to mouse heart development based on the analysis of the atlas. However, establishing an atlas that covers more time points, conducting further research on heart regeneration and heart asymmetry–related studies, and performing functional validation and mechanistic analysis are potential future directions based on our study.

## Methods

### Experimental animal mice and heart sample preparation

The Institutional Animal Care and Use Committee of BGI thoroughly reviewed and granted approval for the animal experimental protocol. All procedures pertaining to mouse experiments in this study strictly adhered to the ethical regulations and guidelines outlined in the animal experimentation protocols of BGI, in addition to compliance with the Guidelines for the Care and Use of Laboratory Animals in China (license number BGI-IRB A21030-T1). All mice were housed in standard Specific Pathogen Free (SPF) conditions with temperatures of 65–75°F (∼18–23°C) and with 40–60% humidity. In this study, male C57BL/6 mice at embryonic day 20 (E20), postnatal day 1 (P1), postnatal day 4 (P4), and postnatal day 14 (P14) were used; 2 mice were used for each experiment.

We performed the following steps: (i) anesthetize mice with chloral hydrate, then extract the hearts and place them in precooled 1× phosphate-buffered saline (PBS); (ii) thoroughly clean the heart surfaces, ensuring the removal of blood; (iii) employ gauze to absorb any excess 1× PBS from the heart surfaces; and (iv) subsequently, position the hearts in a dish on ice, awaiting subsequent processing steps, such as embedding.

### Stereo-seq sample and library preparation

The hearts of mice were dissected from the thoracic cavity at 4 time points, the pericardium was removed using fine forceps, the surface, and intracardiac blood were rinsed in cold PBS and blotted dry with gauze, and the hearts were embedded in Tissue-Tek O.C.T. Compound (Sakura, 4583) and rapidly frozen on dry ice.

The Stereo-seq libraries were prepared using Stereo-seq [13]. Briefly, the OCT-embedded heart was longitudinally sectioned into 10-mm-thick slices using a Leica CM1950 cryostat. These slices were then adhered to the Stereo-seq chip. The chip was completely immersed in methanol at −20°C for 30 minutes. Afterward, the tissue was fixed in methanol at −20°C for 30 minutes. Subsequently, the tissue sections affixed to the chip underwent permeabilization (6 minutes for E20 and P01, 7 minutes for P04, and 9 minutes for P14). Probes on the chip, equipped with coordinate tags, captured the polyA RNA released *in situ* from the tissue sections. Following this step, the captured polyA RNA underwent *in situ* reverse transcription, resulting in the synthesis of cDNA with coordinate tags. Subsequently, after tissue digestion to eliminate any residual sliced tissue from the chip, cDNA with coordinate tags was liberated using release enzymes and recovered utilizing magnetic beads. Following cDNA amplification, 20 ng cDNA was employed for fragmentation and additional amplification to finalize library construction. Ultimately, sequencing was executed using the MGI DNBSEQ sequencing platform.

### HE staining

The HE staining was performed following standard protocols [52]. In total, 10-μm OCT frozen sections were mounted on glass slides and fixed in 4% paraformaldehyde in 1× PBS at room temperature for 10 minutes. Subsequently, the sections were incubated in hematoxylin for 7 minutes, washed in nuclease-free water, incubated in eosin for 2 minutes, and washed again in nuclease-free water. After air-drying at room temperature, bright-field images were captured using a Motic fluorescence microscope.

### Binning data of spatial Stereo-seq data

The Stereo-seq transcriptomic data in this study underwent processing using the Stereo-seq Analysis Workflow software suite (RRID:SCR_025001) [53]. This suite facilitated the mapping of sequencing reads onto tissue sections, allowing for the quantitative assessment of gene expression levels at each spatial position (Spot). Typically, the “bin_size” parameter is configured to group nanochannels within a specific range into a bin unit. Through a statistical analysis of cell and bin sizes across all tissues, we selected an individual bin size of 50 (∼25 μm) as the fundamental unit for downstream analysis of the transcriptomic data generated by Stereo-seq in mouse hearts [13]. This ensured that each unit contained a sufficient number of genes to represent its molecular characteristics. Moreover, in the data quality control phase, we excluded bin 50 units with low gene counts. By examining the gene count distribution curve for each bin 50, we observed that low-quality bin 50 units tended to cluster into a small peak, and we determined the filtering threshold as the minimum value on the right side of this peak.

### Cell clustering and annotation

The downstream analysis of the mouse heart transcriptomic data primarily involved the Seurat software package (version 4.1.1) [54, 55]. Initially, we utilized the “SCTransform” function for spatial section data normalization, scaling, and integration across distinct datasets from various time points. Then we integrated the Seurat objects of each chip for downstream analysis. Subsequently, Principal Component Analysis (PCA) reduction and UMAP embedding were applied for dimensionality reduction. The “FindNeighbors” function (utilizing the top 30 principal components) performed cell clustering, followed by the “FindClusters” function for graph-based clustering. Marker genes for different clusters were identified using the “FindAllMarkers” function in Seurat with parameters set to (min.pct=0.1, logfc.threshold=0.25), and a filter based on *P*adj < 0.05 was applied. Cell clusters were determined using established cell-type-specific markers for each cluster. For a more nuanced categorization of cell types, bins associated with a particular cell type, or groups comprising related cell types, underwent additional clustering and annotation. The subcluster annotation process for a specific cell type mirrors the description provided earlier for general cell types.

### Cell chat (ligand–receptor analysis)

We utilized the CellChat package (version 1.5.0) [56] for the inference, analysis, and visualization of the cell–cell communication network in the mouse heart transcriptomic dataset. Initially, we created a new CellChat object for each time point using the CellChatDB.mouse database within the Seurat framework. This database comprises 2,021 validated molecular interactions, encompassing 60% secreted autocrine/paracrine signaling interactions, 21% extracellular matrix–receptor interactions, and 19% cell–cell contact interactions. After preprocessing the expression data by identifying overexpressed genes and interactions, we employed the “computeCommunProb” and “filterCommunication” functions to infer the cellular communication network and compute communication probabilities. The “computeCommunProbPathway” function calculated communication probabilities at the signaling pathway level by summarizing probabilities associated with ligand–receptor interactions in each pathway. For an aggregated view of the cell–cell communication network, the “aggregateNet” function was used to count links or summarize communication probabilities for each communication pair. Subsequently, the 3 CellChat objects for each time point were merged using the “mergeCellChat” function for downstream analysis.

### Cell differentiation inference

To facilitate subsequent trajectory analysis and transcription factor analysis, we randomly selected 500 cells from each subtype of the 3 cell types (FBs, aCMs, vCMs) using the “subset” function with the “downsample” parameter of the SeuratObject [Satija R] package. To analyze the differentiation trajectory of mouse heart cells and investigate their pseudotime relationships, we employed the R package Monocle 2 (version 2.22.0) [57]. Initially, we used the “importCDS” function (object, import_all = F) to convert the raw counts into the CellDataSet format. Subsequently, the “estimateSizeFactors” and “estimateDispersions” functions were employed to precompute crucial parameters related to the data. The “differenceGeneTest” function was used to select potential ordering genes (*q* < 0.01), providing information for ordering cells along the pseudotime trajectory. Dimensionality reduction and clustering analysis were performed using the “reduceDimension” function (reduction_method = “DDRTree”), with “fullModelFormulaStr” set for cell type and time point. Trajectory inference was executed using the default parameters through the “orderCells” function.

### Differential gene expression analysis and GO enrichment

Differential expression analysis of the same cell type between 2 time points was conducted using the “FindMarkers” function in Seurat packages. We filtered significant differentially expressed genes with an adjusted *P* < 0.05 and an absolute average log_2_ fold change (FC) > 0.5. Specifically for the identification of the left and right atria, differential analysis was performed using data from the corresponding spatial sections. The “FindMarkers” function in the Seurat package was employed, focusing on highly variable genes based on criteria such as *P* < 0.05 and log_2_FC > 0.5. For a deeper understanding of the functional implications of these genes, we conducted GO enrichment analysis in the Biological Process category. This was achieved using the clusterProfiler software package (version 4.7.1) [58], with gene annotations sourced from the org.Mm.eg.db annotation database for mouse loci.

### Transcription factor regulation activity prediction

We utilized pySCENIC [59] for transcription factor analysis across 3 cell types (FBs, aCMs, and vCMs). Initially, we downloaded the relevant cisTarget database for mice with version mm10 [60]. This database was constructed using the 2022 SCENIC+ motif collection, defining the search space as 500 bp upstream of the transcription start site (TSS) and 100 bp downstream around the TSS of the gene where the motif is scored.

We inferred potential TFs and computed the transcription factor activity for each cell using the default workflow of pySCENIC [61] (RRID:SCR_025802). Subsequently, the SCENIC package (v1.3.1) [59] in R was employed to calculate the regulon specificity score of the transcription factors at different time points. The transcription factor activity was integrated with the gene expression matrix, and the correlation coefficient between the transcription factors and target genes was determined using the cor.test function from the stats (v4.3.0) [62] package.

## Supplementary Material

giaf012_Supplement_Files

giaf012_GIGA-D-24-00351_Original_Submission

giaf012_GIGA-D-24-00351_Revision_1

giaf012_Response_to_Reviewer_Comments_Original_Submission

giaf012_Reviewer_1_Report_Original_Submission Joseph C. Wu -- 10/13/2024

giaf012_Reviewer_2_Report_Original_Submission Yuliang Feng -- 11/27/2024

giaf012_Reviewer_2_Report_Revision_1 Yuliang Feng -- 12/20/2024

## Data Availability

All raw data generated by this study can be found in NCBI-SRA with the accession number PRJNA1148773. Processed data have been deposited in the China National GeneBank Database (CNGBdb) in the China National GeneBank Sequence Archive (CNSA) with the accession number STT0000062. All spatial transcriptomic data of mouse embryo samples (E9.5, E10.5, E11.5, and E12.5) are available at NCBI Gene Expression Omnibus with accession number GSE178636. All supporting data and materials are available in the *GigaScience* repository, GigaDB [63].

## Additional Files

**Supplementary Fig. S1**. Cell-type distribution in the mouse heart revealed by Stereo-seq. (a) Spatial and temporal distribution of 22 mouse heart sections across 4 time points (E20, P01, P04, P14). (b) UMAP plots for cell clustering and annotation of 4 time points (E20, P01, P04, P14).

**Supplementary Fig. S2**. Signaling pathways of different developmental stages. (a) The overall information flow of each signaling pathway at 4 time points.

**Supplementary Fig. S3**. Cell–cell interactions of different developmental stages. (a) Interaction strength among different cell types and interactions between receptors and ligands in 3 cell types at E20. (b) Interaction strength among different cell types and interactions between receptors and ligands in 3 cell types at P01. (c) Interaction strength among different cell types and interactions between receptors and ligands in 3 cell types at P04. (d) Interaction strength among different cell types and interactions between receptors and ligands in 3 cell types at P14.

**Supplementary Fig. S4**. Cell-type distribution in the mouse heart during development. (a) Spatial visualization of representative marker genes specifically expressed in the pericardial cells on the surface of the mouse heart at 4 time points (E20, P01, P04, P14). (b–d) At 4 time points (E20, P01, P04, P14), spatial visualization depicting the annotation of subtypes for vCM4, aCM5, and FB4 subclusters. (e–g) At 4 time points (E20, P01, P04, P14), spatial visualization displays the locations of 3 subclusters (vCM4, aCM5, and FB4) in the mouse heart.

**Supplementary Fig. S5**. Trajectory analysis and GO enrichment analysis of different developmental stages. (a) Trajectory analysis of aCM, vCM, and FB at 4 time points (E20, P01, P04, and P14). (b) Trajectory analysis of subtypes among aCM, vCM, and FB at 4 time points (E20, P01, P04, and P14).

**Supplementary Fig. S6**. Development of myocardial cells in early mouse embryos. (a) UMAP of spatial transcriptomic data in mouse embryos. (b) Expression of myocardial cell marker genes in clusters of spatial data in mouse embryos. (c) Spatial distribution of myocardial cells (clusters 8 and 16) in mouse embryos. (d) Expression of heart development-regulated genes in early embryonic myocardial cells.

**Supplementary Fig. S7**. Accuracy analysis for the identification of the left and right atria. (a) Cell clustering without utilizing spatial position information and annotation of the left and right atria using spatial position information at the E20. (b) Cell clustering without utilizing spatial position information and annotation of the left and right atria using spatial position information at the P01. (c) Cell clustering without utilizing spatial position information and annotation of the left and right atria using spatial position information at the P04. (d) Cell clustering without utilizing spatial position information and annotation of the left and right atria using spatial position information at the P14. (e) Functional enrichment analysis of the identified genes in the left and right atria at 4 time points (E20, P01, P04, and P14)

**Supplementary Fig. S8**. Spatial distribution of differentially expressed genes between left and right atria. (a) Spatial visualization depicting the expression patterns of *Pitx2* in the heart. (b) Spatial visualization depicting the expression patterns of *Bmp10* in the heart. (c) Spatial visualization depicting the expression patterns of *Eng* in the heart. (d) Spatial visualization depicting the expression patterns of *Adamts8* in the heart.

**Supplementary Table S1**. The marker genes and the number of cells used in cellular annotation.

**Supplementary Table S2**. Gene counts and count numbers for different cell types at 4 time points.

**Supplementary Table S3**. GO enrichment of different subtypes of ventricular cardiomyocyte.

**Supplementary Table S4**. GO enrichment of different subtypes of atrial cardiomyocyte.

**Supplementary Table S5**. GO enrichment of different subtypes of fibroblast cells.

**Supplementary Table S6**. Interactions among different cell types at 4 time points.

**Supplementary Table S7**. Driver genes involved in the differentiation process of fibroblasts and cardiomyocytes.

**Supplementary Table S8**. Inference of transcription factor activity in different subtypes of fibroblasts and cardiomyocytes.

**Supplementary Table S9**. The annotation accuracy of left and right atrial cells.

## Abbreviations

aCM: atrial cardiomyocyte; CADM: cell adhesion molecule; FB: fibroblast; FC: fold change; GO: Gene Ontology; HE: hematoxylin and eosin; NCAM: neural cell adhesion molecule; PBS: phosphate-buffered saline; RSS: regulon specificity score; Stereo-seq: SpaTial Enhanced REsolution Omics-sequencing; TF: transcription factor; TSS: transcription start site; UMAP: uniform manifold approximation and projection; vCM: ventricular cardiomyocyte.
