# Supplementary material for: Exploring the cellular and molecular basis of murine cardiac development through spatiotemporal transcriptome sequencing
Source: Gigascience. 2025 Feb 17;14:giaf012. doi: 10.1093/gigascience/giaf012 (PMC11831923; doi:10.1093/gigascience/giaf012)
Supplement: giaf012_Supplement_Files [file giaf012_supplement_files.zip › Supplementary data_V2.docx]

# Supplementary Information for:

# Exploring the Cellular and Molecular Basis of Murine Cardiac Development through Spatiotemporal Transcriptome Sequencing

## Jingmin Kang^1,2,*^, Qingsong Li^1,2,*^, Jie Liu^3,*^, Lin Du^1,4,*^, Peng Liu^1^, Fuyan Liu^1,2^, Yue Wang^1,2,5^, Xunan Shen^1,2^, Xujiao Luo^1^, Ninghe Wang^6^, Renhua Wu^6^, Jizheng Wang^7,#^, Lei Song^3,7,8,#^, Xin Liu^1,2,#^

^1^ BGI Research, Beijing, China

^2^ BGI Research, Shenzhen, China

^3^ Cardiomyopathy Ward, Fuwai Hospital, National Center for Cardiovascular Disease, Chinese Academy of Medical Science and Peking Union Medical College, 167, Beilishilu, Xicheng District, Beijing 100037, China

^4^ College of Life Sciences, University of Chinese Academy of Sciences, Beijing 100049, China

^5^ State Key Laboratory of Quality Research in Chinese Medicine and Institute of Chinese Medical Sciences, University of Macau, Macao, China

^6^ Clin Lab, BGI Genomics, Tianjin, China

^7^ State Key Laboratory of Cardiovascular Disease, Fuwai Hospital, National Center for Cardiovascular Diseases, Chinese Academy of Medical Sciences and Peking Union Medical College, 167, Beilishi Road, Xicheng District, Beijing 100037, China

^8^ National Clinical Research Center of Cardiovascular Diseases, Fuwai Hospital, National Center for Cardiovascular Diseases, Chinese Academy of Medical Sciences and Peking Union Medical College, 167, Beilishilu, Xicheng District, Beijing 100037, China

* These authors contributed equally.

# Correspondence

## Supplementary Figures

**Supplementary Figure 1. Cell type distribution in the mouse heart revealed by Stereo-seq.**

**Supplementary Figure 2. Signaling pathways of different developmental stages.**

**Supplementary Figure 3. Cell-cell interactions of different developmental stages.**

**Supplementary Figure 4. Cell type distribution in the mouse heart during development.**

**Supplementary Figure 5. Trajectory analysis and GO enrichment analysis of different developmental stages.**

**Supplementary Figure 6. Development of myocardial cells in early mouse embryos.**

**Supplementary Figure 7. Accuracy analysis for the identification of the left and right atria.**

**Supplementary Figure 8. Spatial Distribution of Differentially Expressed Genes Between Left and Right Atria.**

**Supplementary Tables**

**Supplementary Table 1. The marker genes and the number of cells used in cellular annotation.**

**Supplementary Table 2. Gene counts and count numbers for different cell types at four time points.**

**Supplementary Table 3. GO enrichment of different subtypes of ventricular cardiomyocyte.**

**Supplementary Table 4. GO enrichment of different subtypes of atrial cardiomyocyte.**

**Supplementary Table 5. GO enrichment of different subtypes of Fibroblast cells.**

**Supplementary Table 6. Interactions among different cell types at four time points.**

**Supplementary Table 7. Driver genes involved in the differentiation process of fibroblasts and cardiomyocytes.**

**Supplementary Table 8. Inference of transcription factor activity in different subtypes of fibroblasts and cardiomyocytes.**

**Supplementary Table 9. The annotation accuracy of left and right atrial cell.**

**
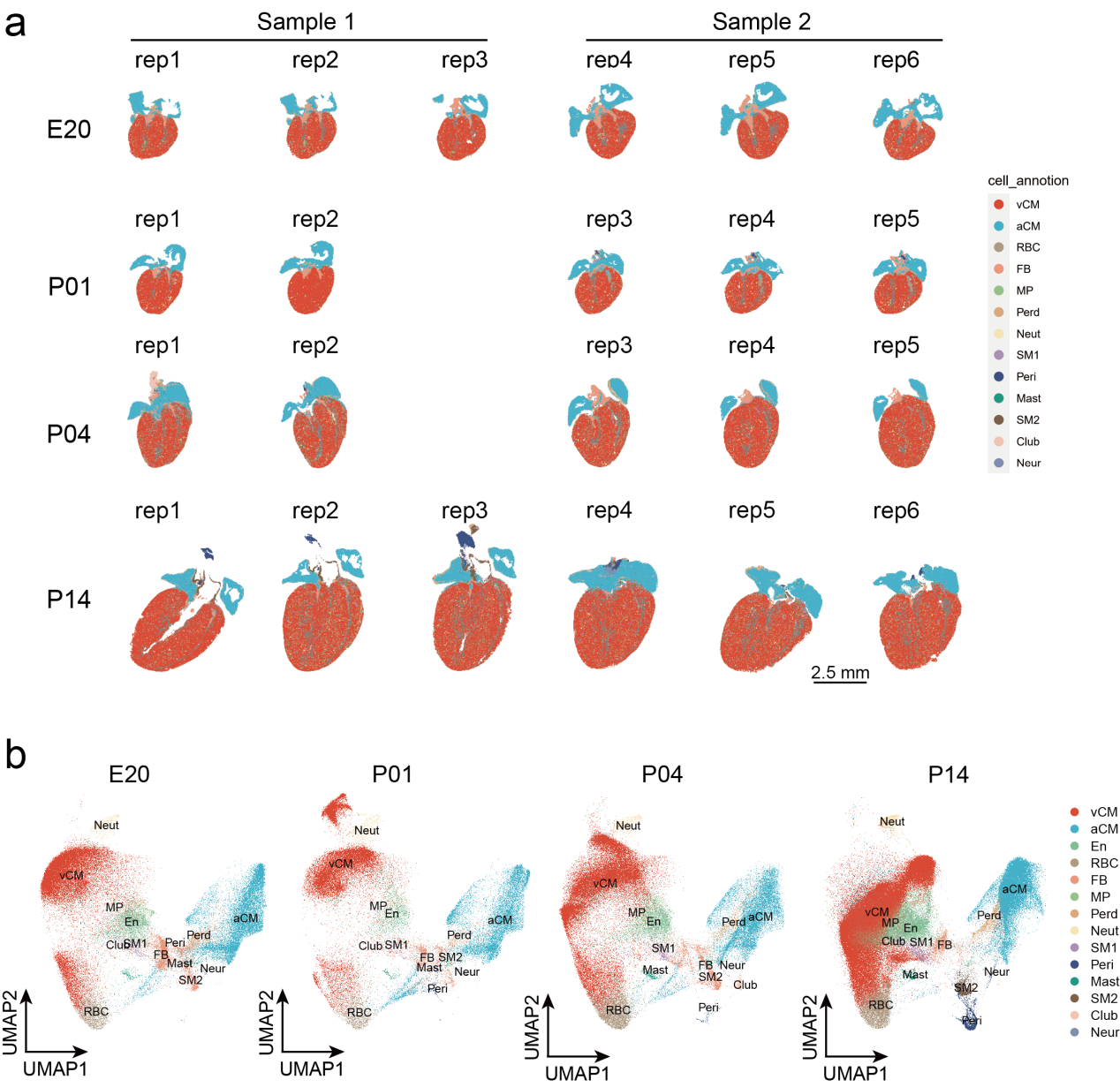
**

**Supplementary Figure 1. Cell type distribution in the mouse heart revealed by Stereo-seq.**

**a.** Spatial and temporal distribution of 22 mouse heart sections across 4 time points (E20, P01, P04, P14). **b.** UMAP plots for cell clustering and annotation of 4 time points (E20, P01, P04, P14).


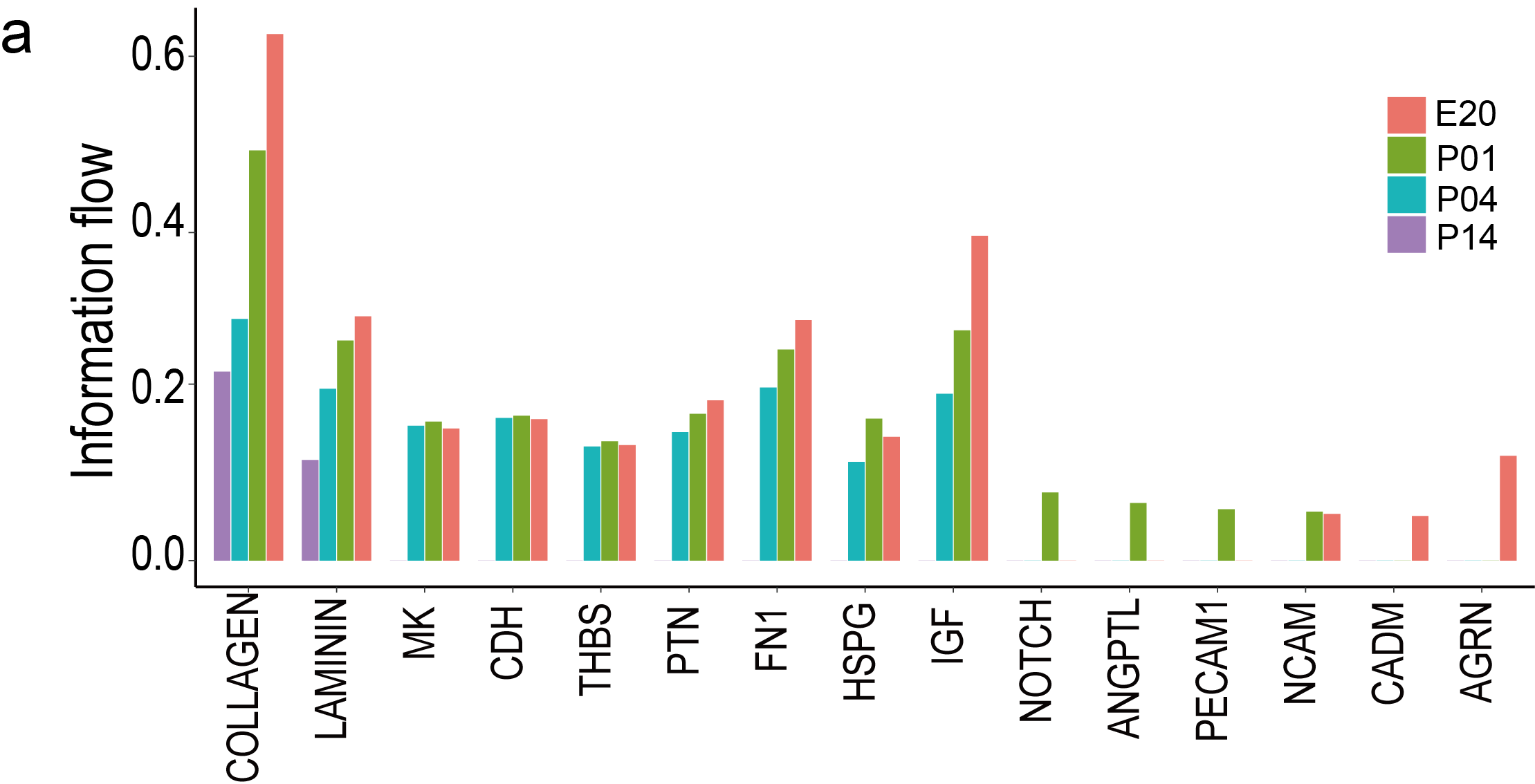


**Supplementary Figure 2. Signaling pathways of different developmental stages.**

**a.** The overall information flow of each signaling pathway at four time points


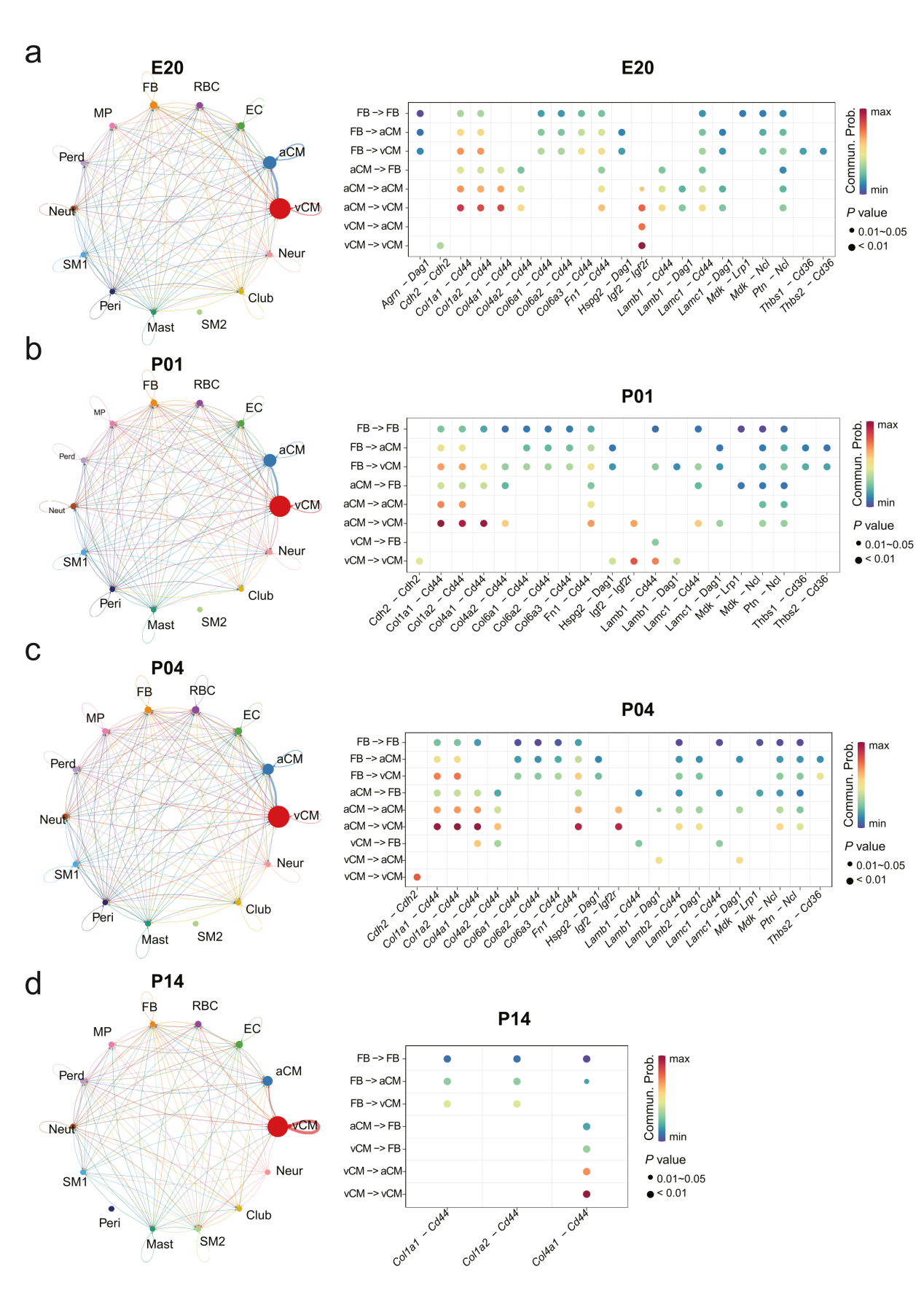


**Supplementary Figure 3. Cell-cell interactions of different developmental stages.**

**a.** Interaction strength among different cell types and interactions between receptors and ligands in three cell types at E20. **b.** Interaction strength among different cell types and interactions between receptors and ligands in three cell types at P01. **c.** Interaction strength among different cell types and interactions between receptors and ligands in three cell types at P04. **d.** Interaction strength among different cell types and interactions between receptors and ligands in three cell types at P14.


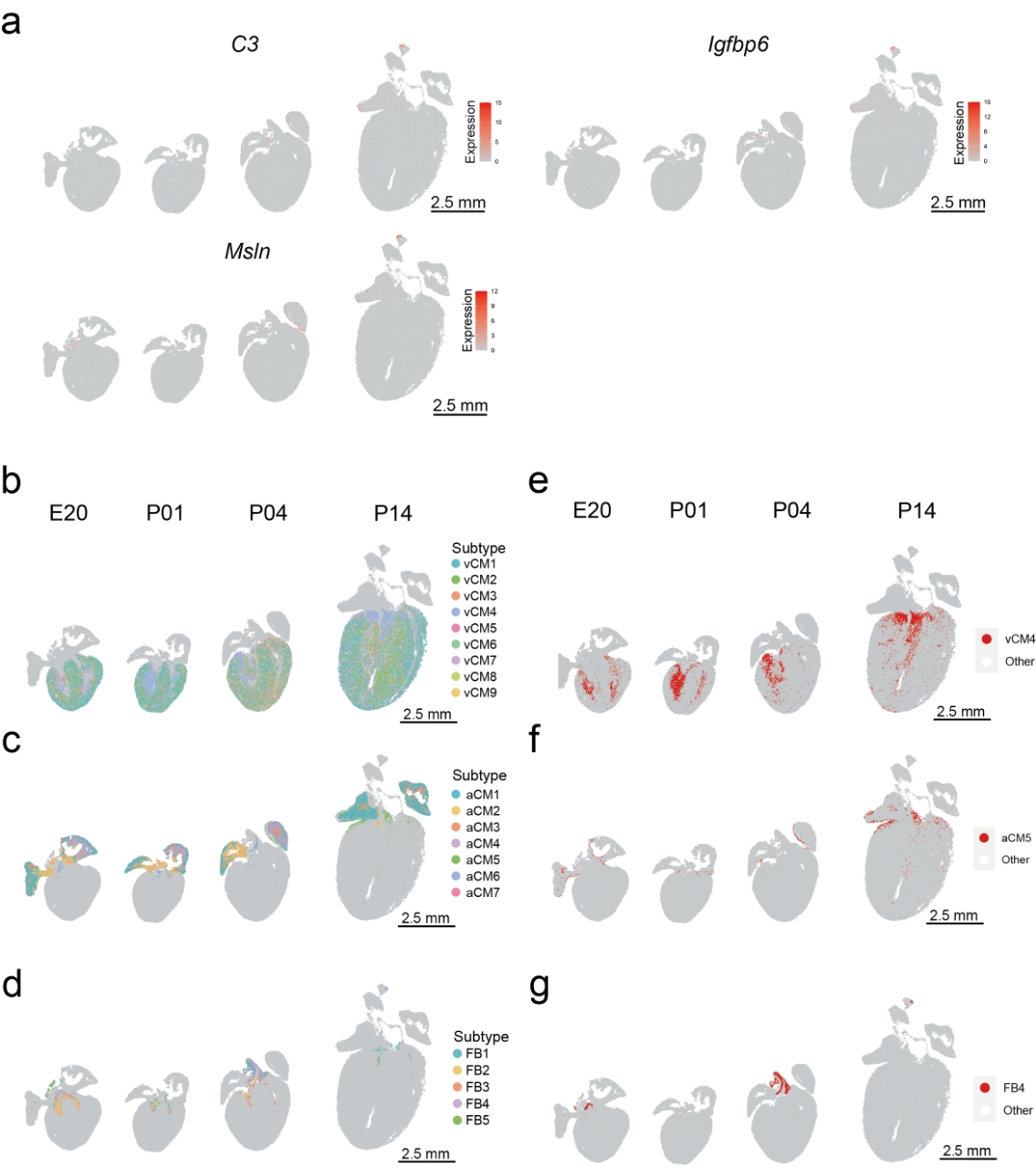


**Supplementary Figure 4. Cell type distribution in the mouse heart during development.**

**a.** Spatial visualization of representative marker genes specifically expressed in the pericardial cells on the surface of the mouse heart at four time points (E20, P01, P04, P14). **b-d.** At four time points (E20, P01, P04, P14), spatial visualization depicting the annotation of subtypes for vCM4, aCM5, and FB4 subclusters. **e-g.** At four time points (E20, P01, P04, P14), spatial visualization displays the locations of three subclusters (vCM4, aCM5, and FB4) in the mouse heart.


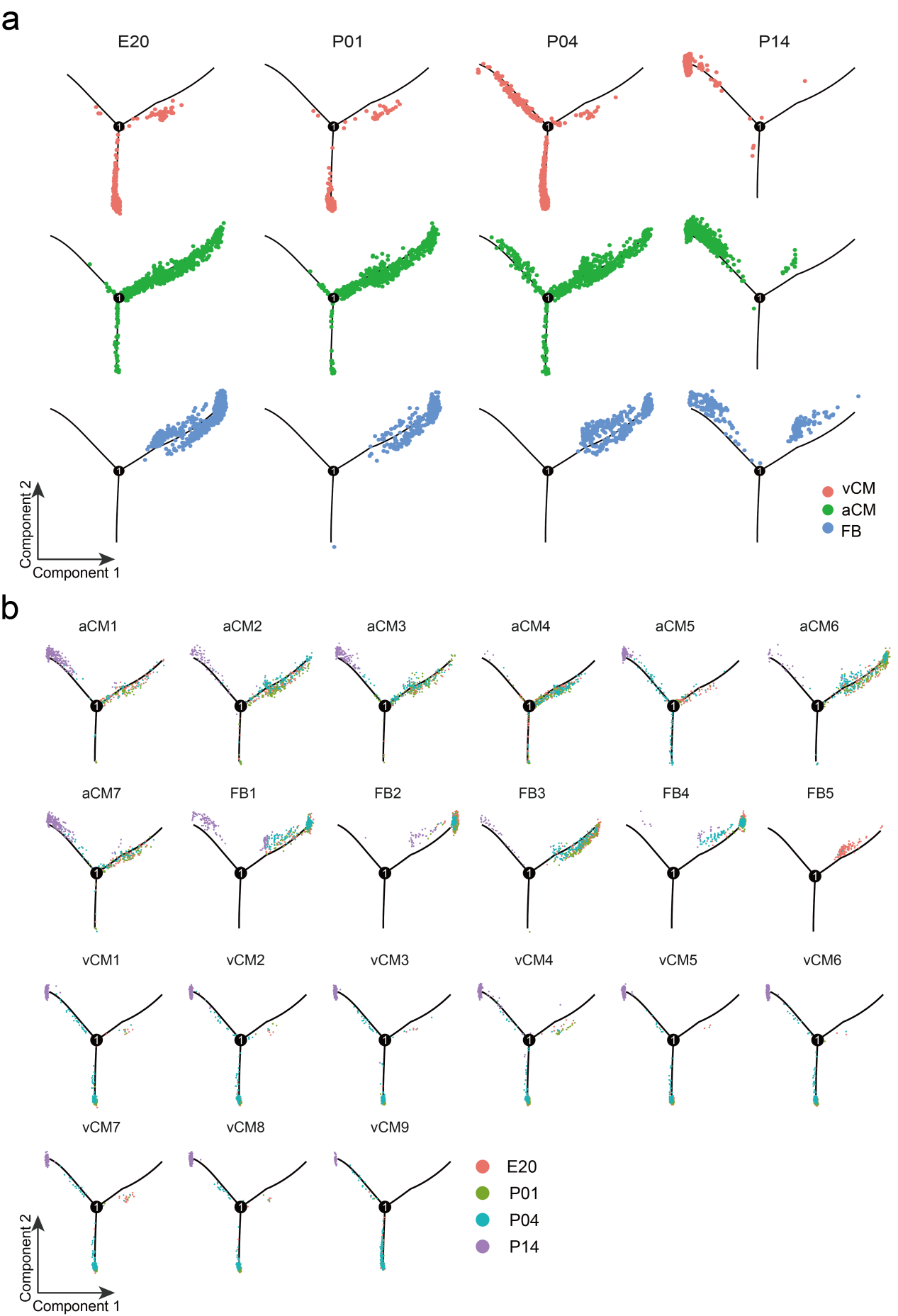


**Supplementary Figure 5. Trajectory analysis and GO enrichment analysis of different developmental stages.**

**a.** Trajectory analysis of aCM, vCM, and FB at four time points (E20, P01, P04, and P14). **b.** Trajectory analysis of subtypes among aCM, vCM, and FB at four time points (E20, P01, P04, and P14).


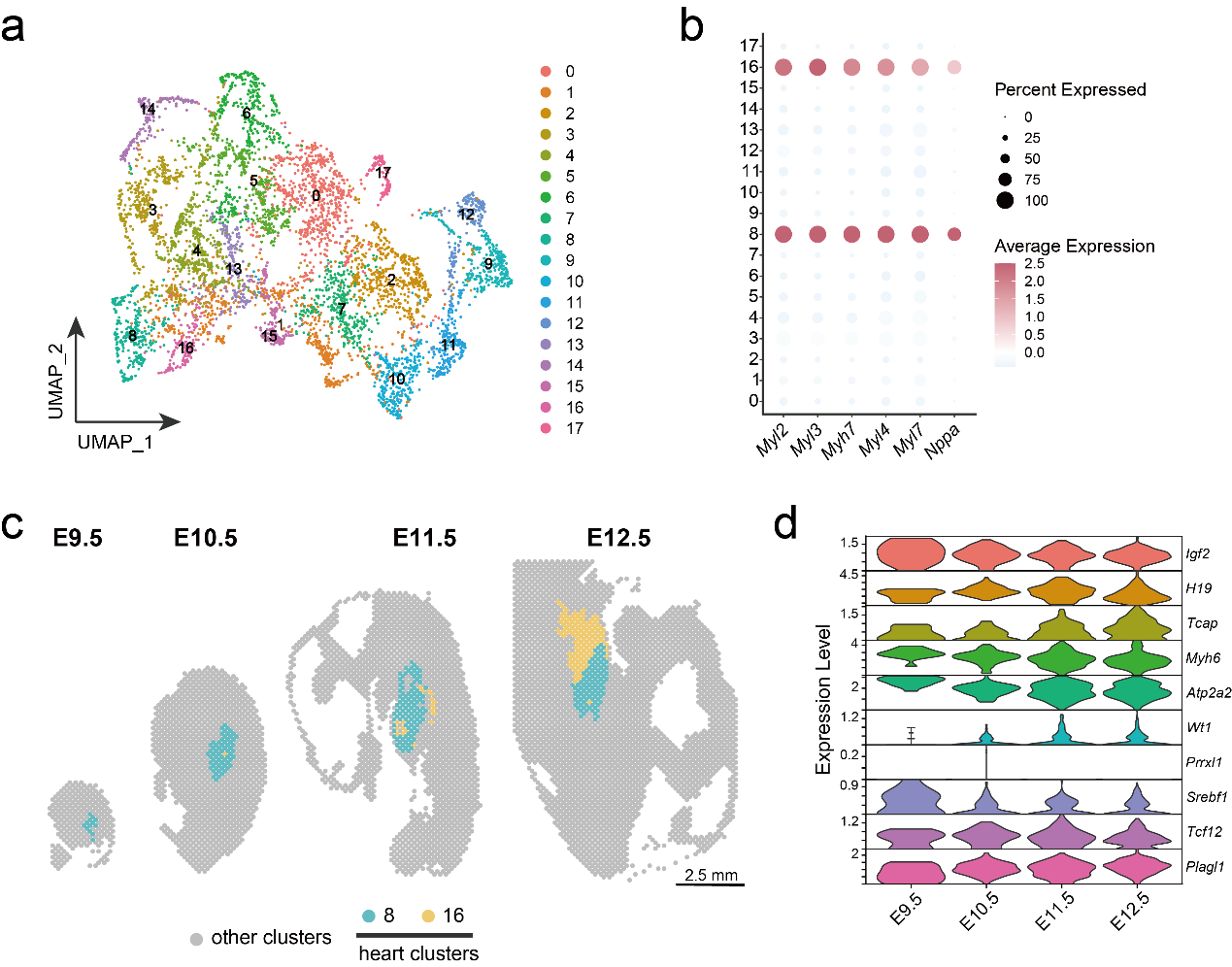


**Supplementary Figure 6. Development of myocardial cells in early mouse embryos.**

**a.** UMAP of spatial transcriptomic data in mouse embryos. **b.** Expression of myocardial cell marker genes in clusters of spatial data in mouse embryos. **c.** Spatial distribution of myocardial cells (clusters 8 and 16) in mouse embryos. **d.** Expression of heart development-regulated genes in early embryonic myocardial cells.


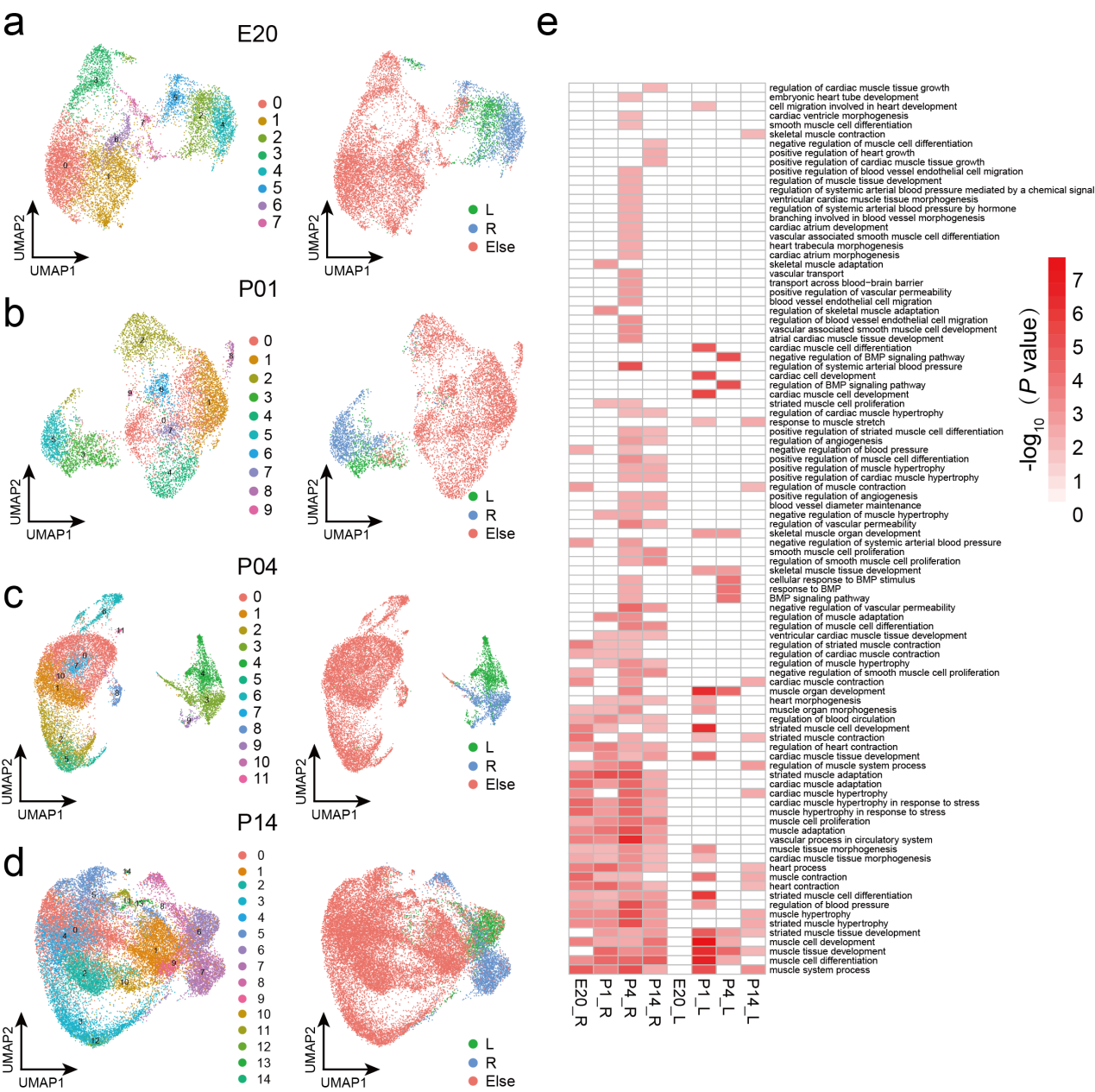


**Supplementary Figure 7. Accuracy analysis for the identification of the left and right atria.**

**a.** Cell clustering without utilizing spatial position information and annotation of the left and right atria using spatial position information at the E20. **b.** Cell clustering without utilizing spatial position information and annotation of the left and right atria using spatial position information at the P01. **c.** Cell clustering without utilizing spatial position information and annotation of the left and right atria using spatial position information at the P04. **d.** Cell clustering without utilizing spatial position information and annotation of the left and right atria using spatial position information at the P14. **e.** Functional enrichment analysis of the identified genes in the left and right atria at four time points (E20, P01, P04, and P14)


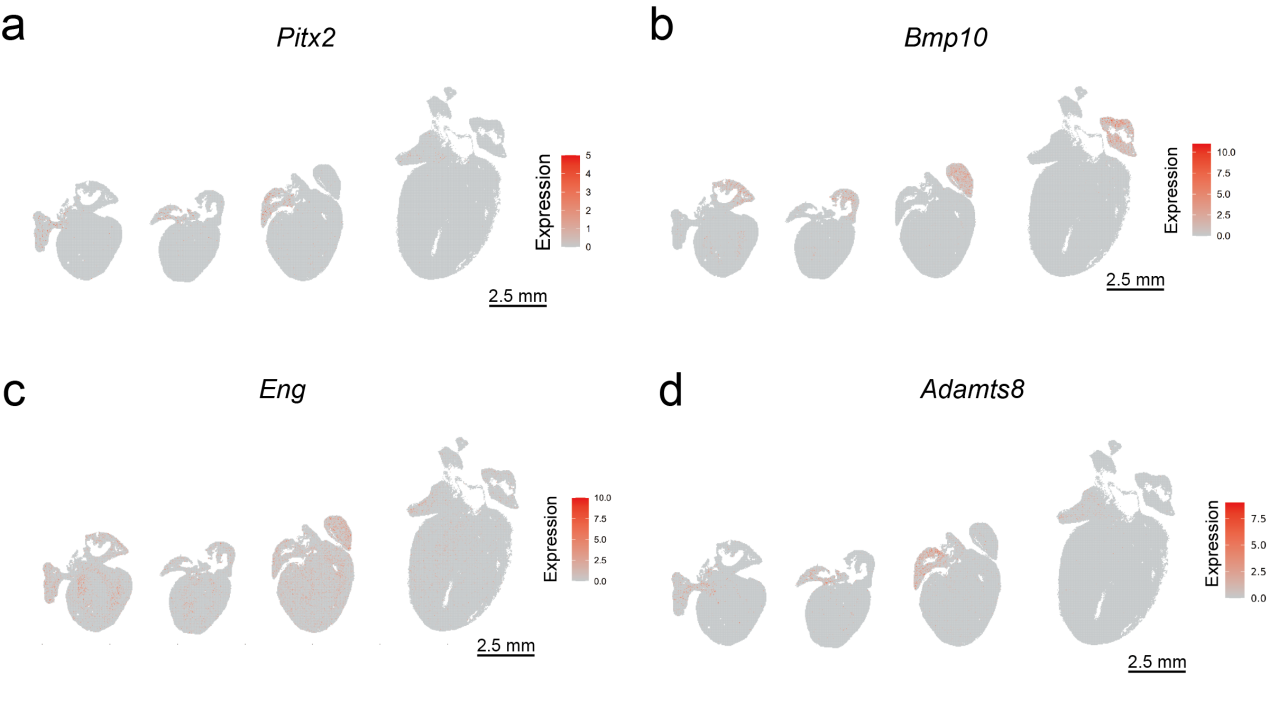


**Supplementary Figure 8. Spatial Distribution of Differentially Expressed Genes Between Left and Right Atria.**

**a.** Spatial visualization depicting the expression patterns of *Pitx2* in the heart. **b.** Spatial visualization depicting the expression patterns of *Bmp10* in the heart. **c.** Spatial visualization depicting the expression patterns of *Eng* in the heart. **d.** Spatial visualization depicting the expression patterns of *Adamts8* in the heart.
