# Supplementary figures and images for: Exploring the cellular and molecular basis of murine cardiac development through spatiotemporal transcriptome sequencing
Source: Gigascience. 2025 Feb 17;14:giaf012. doi: 10.1093/gigascience/giaf012 (PMC11831923; doi:10.1093/gigascience/giaf012)

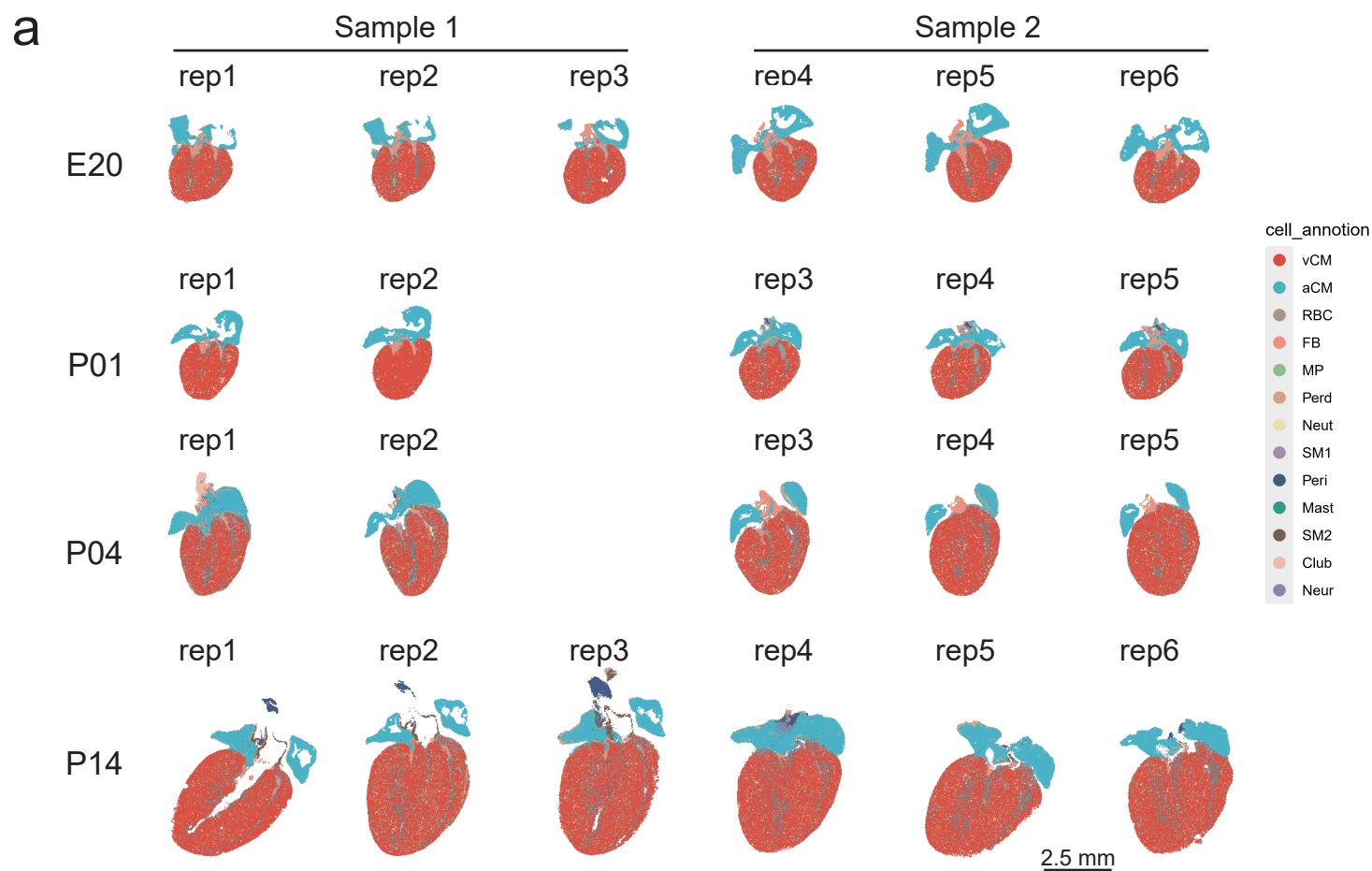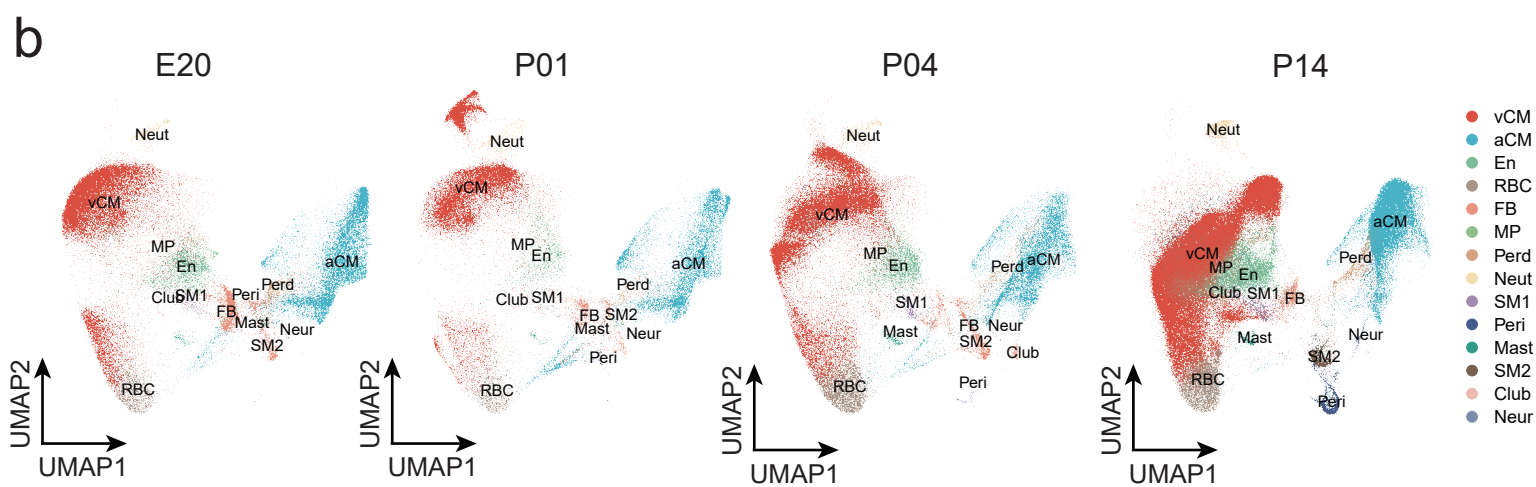

Supplement: giaf012_Supplement_Files [file giaf012_supplement_files.zip › SF1.pdf]

a

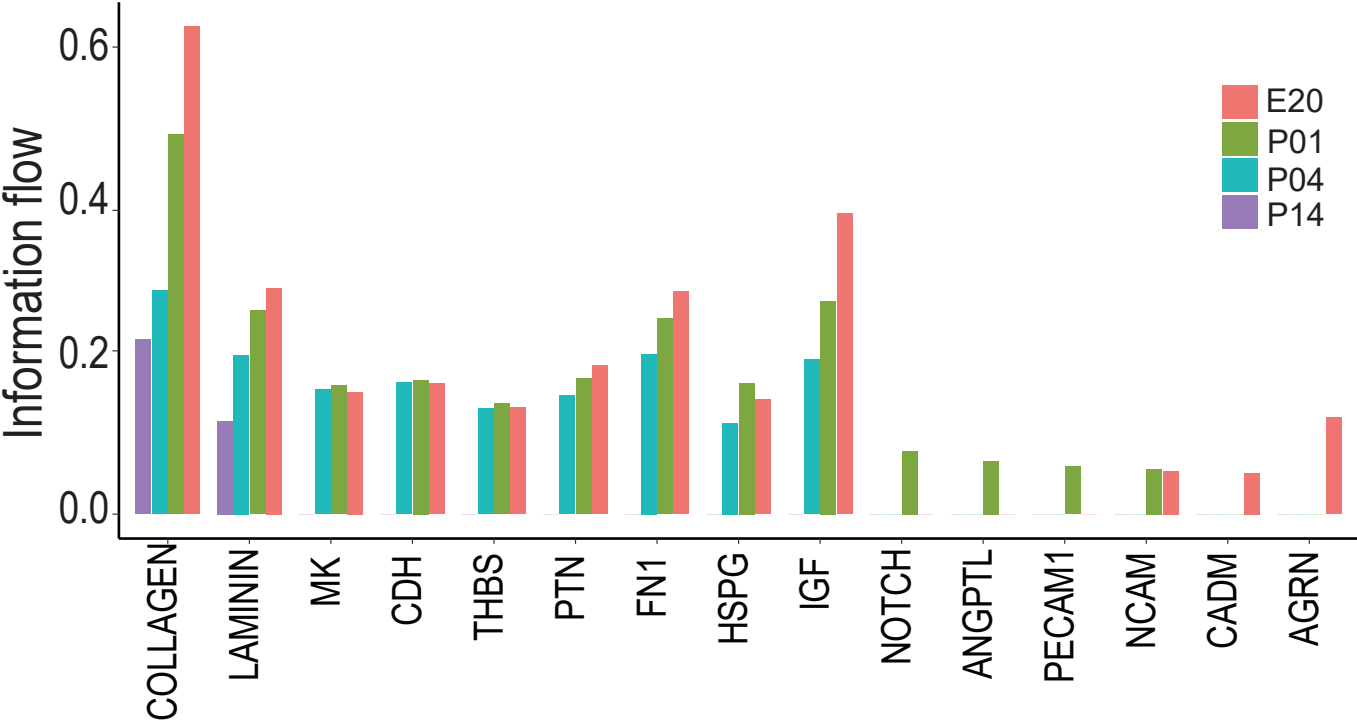

Supplement: giaf012_Supplement_Files [file giaf012_supplement_files.zip › SF2.pdf]

a

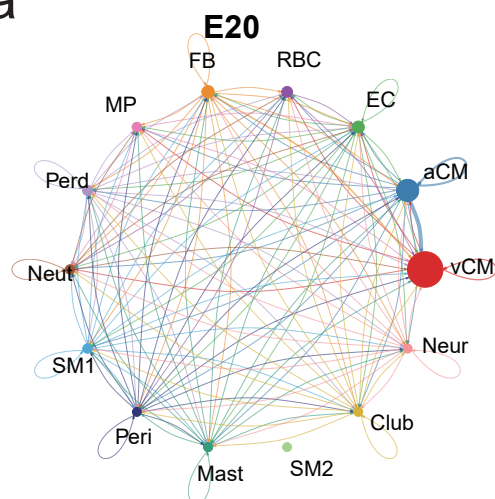

E20

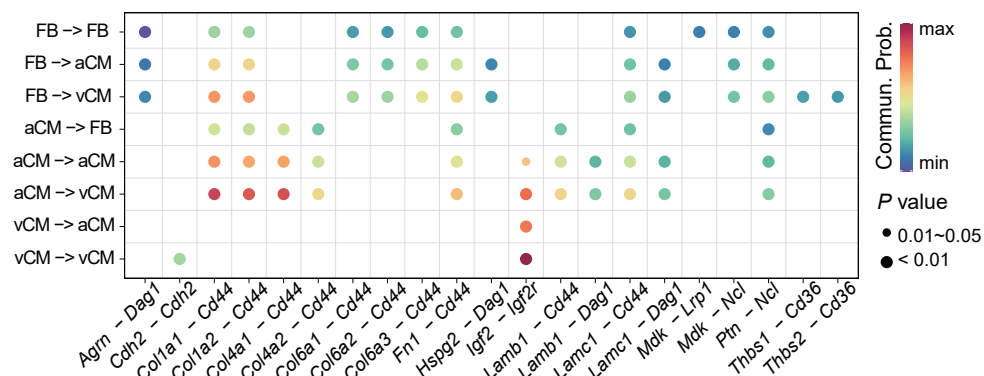

b

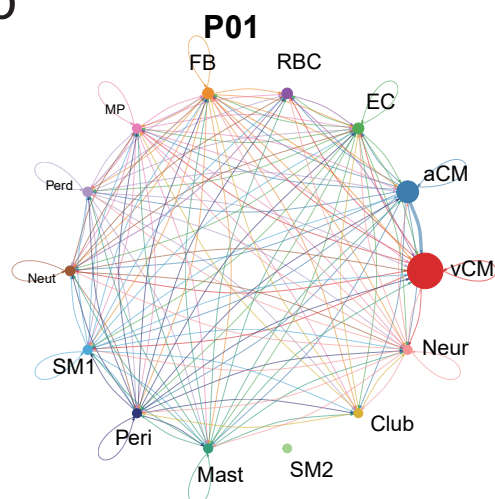

P01

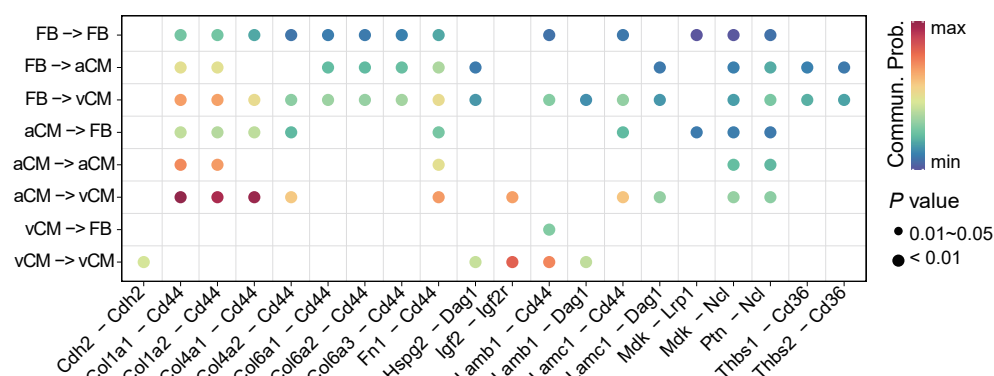

c

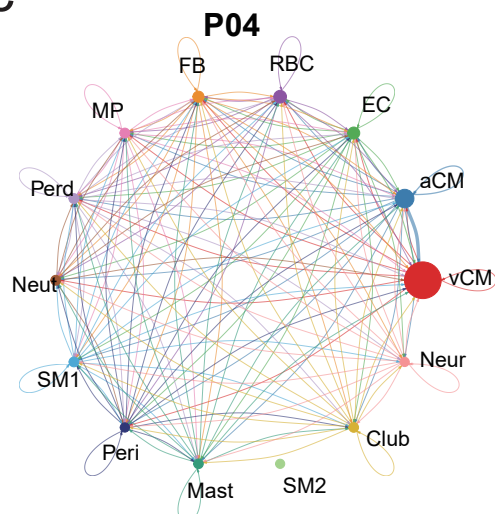

P04

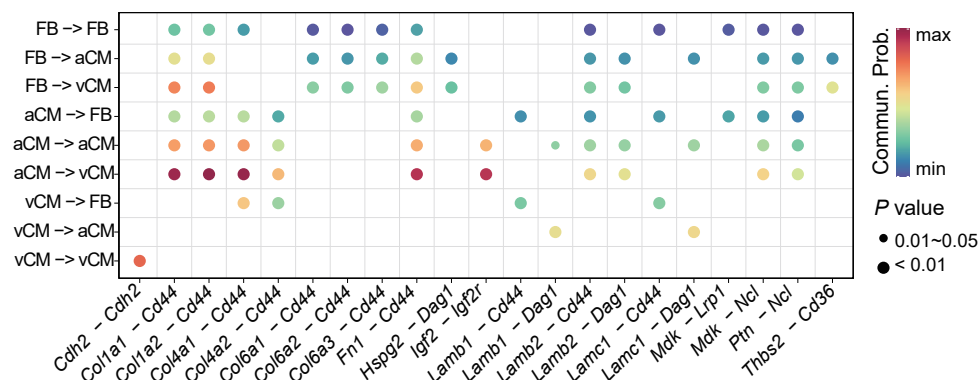

d

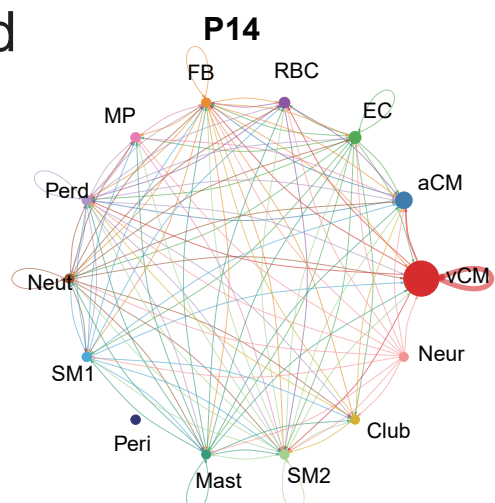

P14

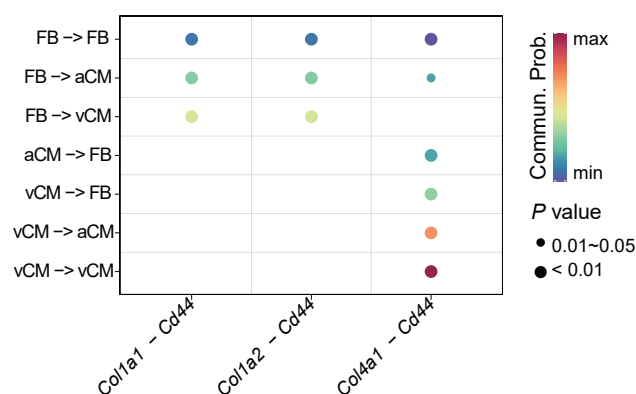

Supplement: giaf012_Supplement_Files [file giaf012_supplement_files.zip › SF3.pdf]

a

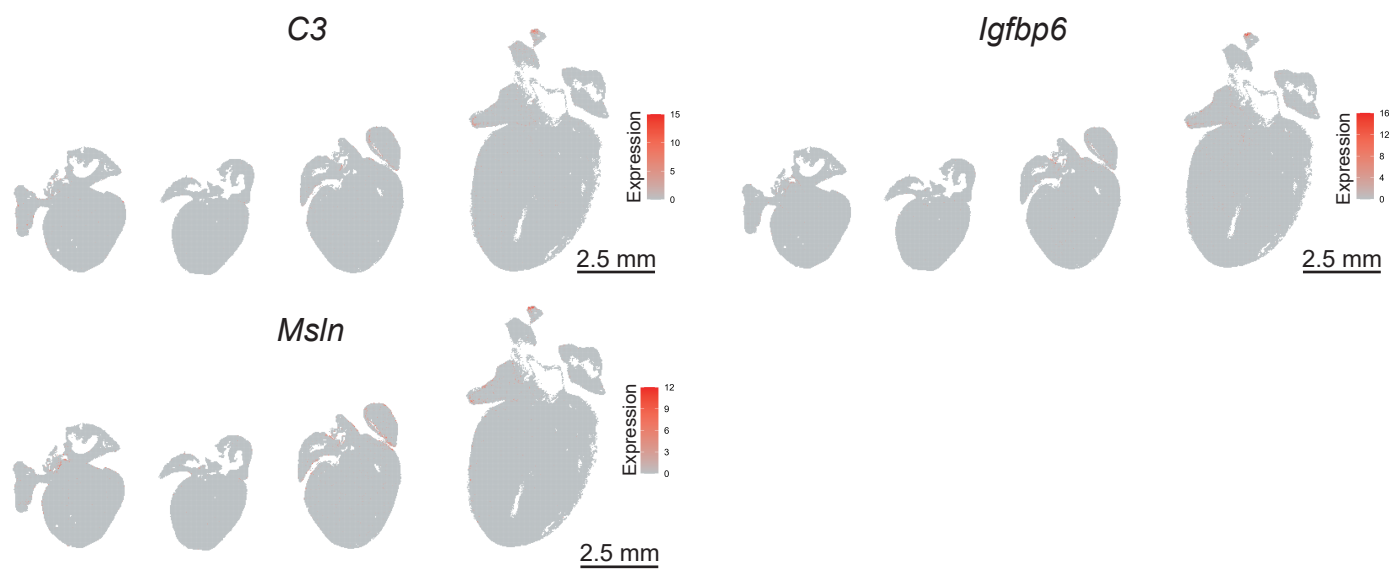

b

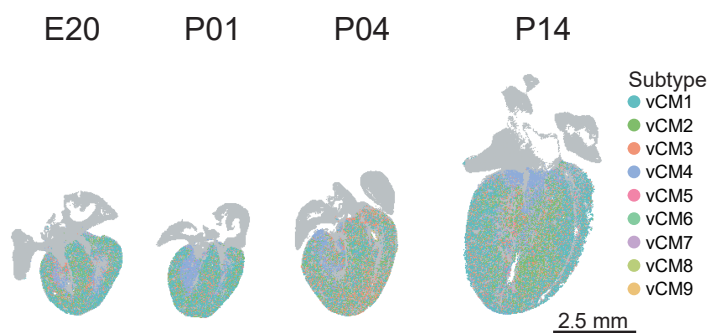

e

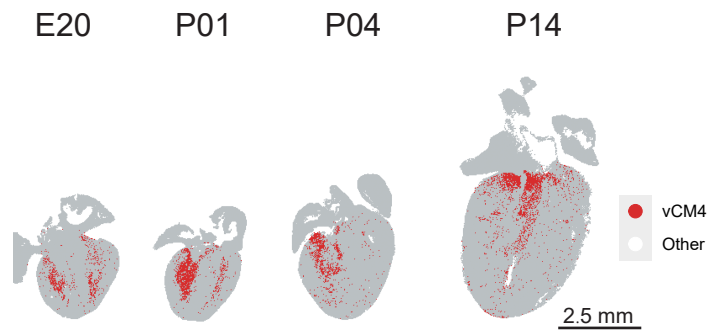

c

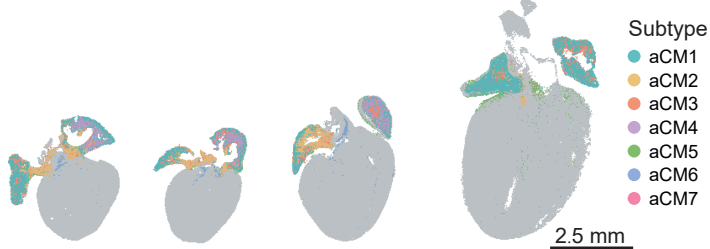

f

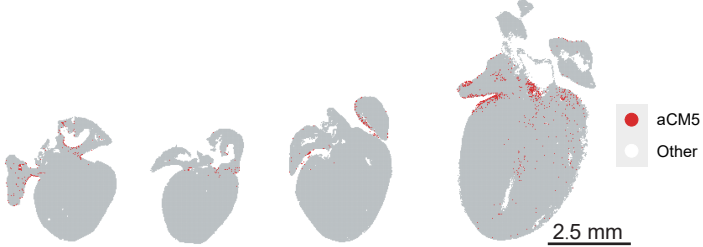

d

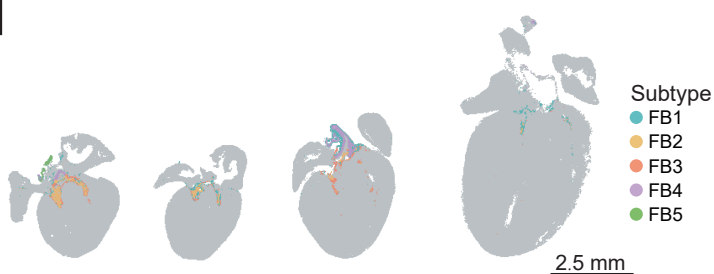

g

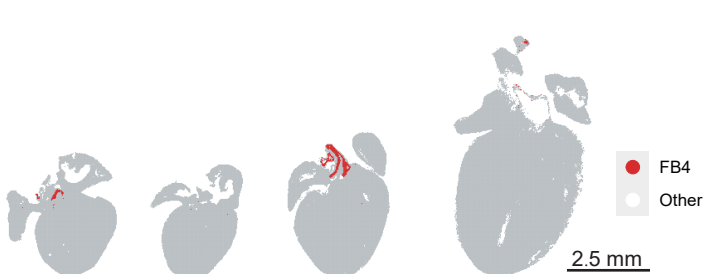

Supplement: giaf012_Supplement_Files [file giaf012_supplement_files.zip › SF4.pdf]

a

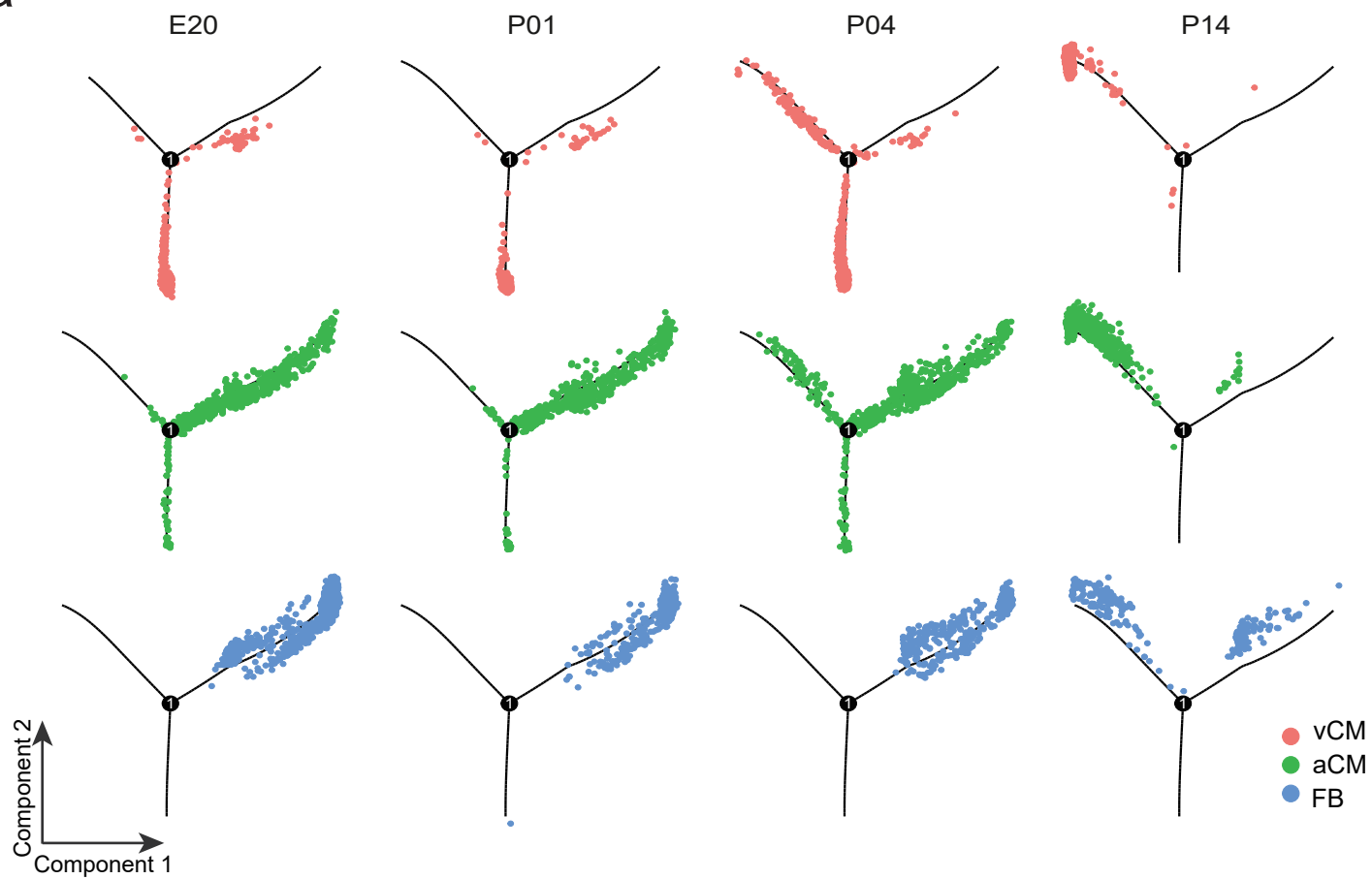

b

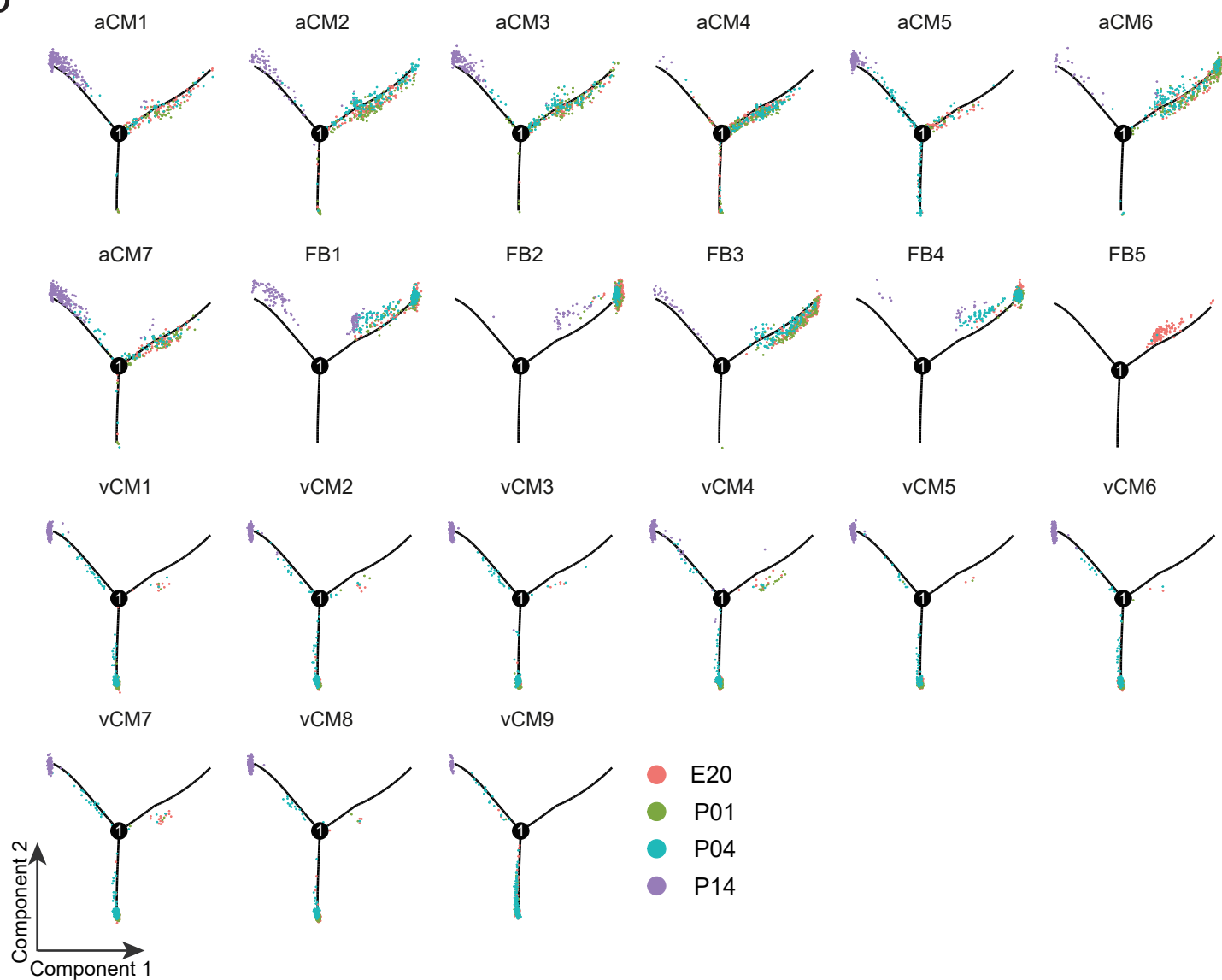

Supplement: giaf012_Supplement_Files [file giaf012_supplement_files.zip › SF5.pdf]

a

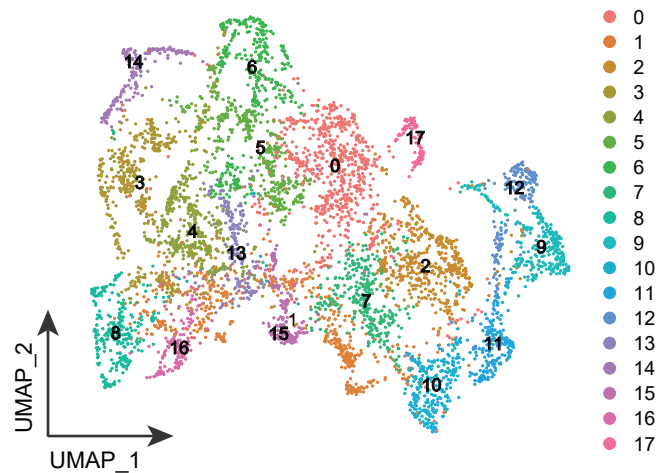

b

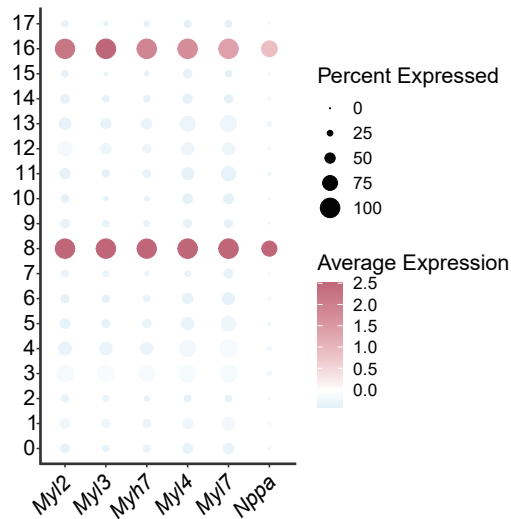

c

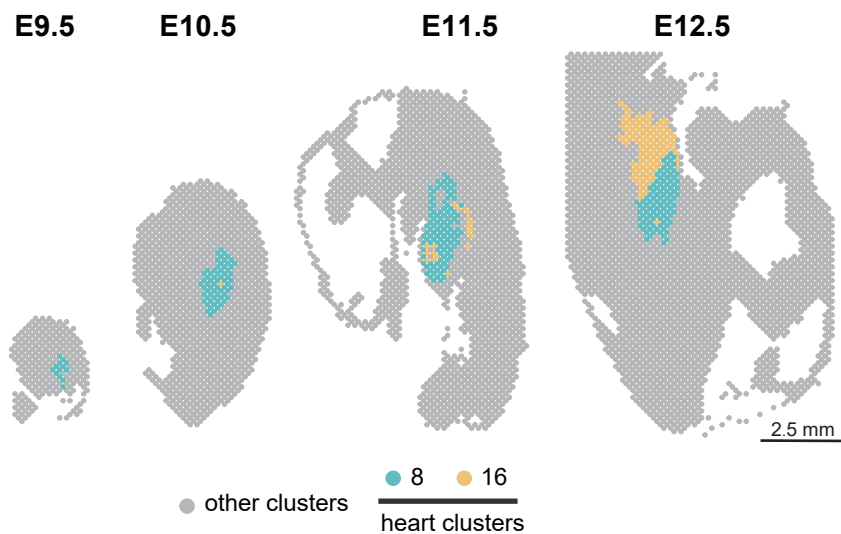

d

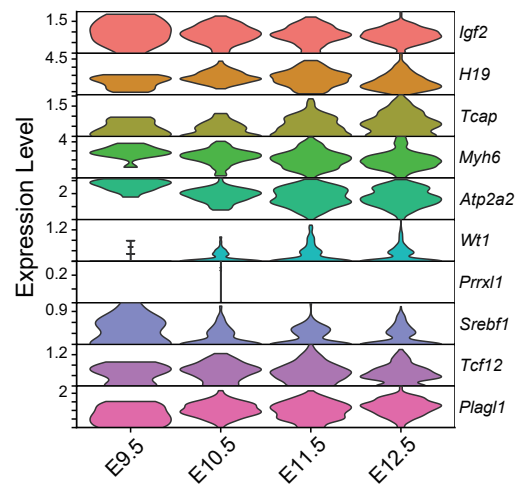

Supplement: giaf012_Supplement_Files [file giaf012_supplement_files.zip › SF6.pdf]

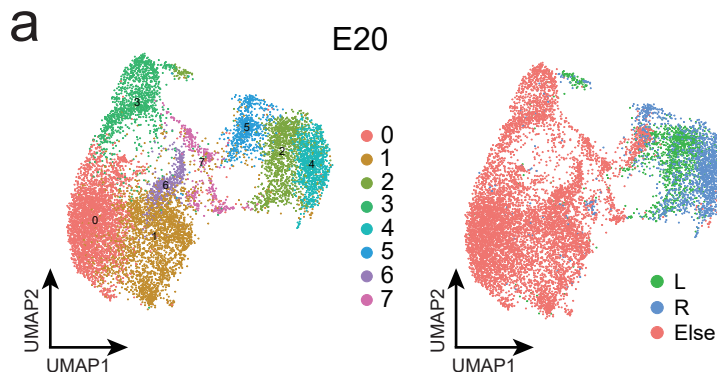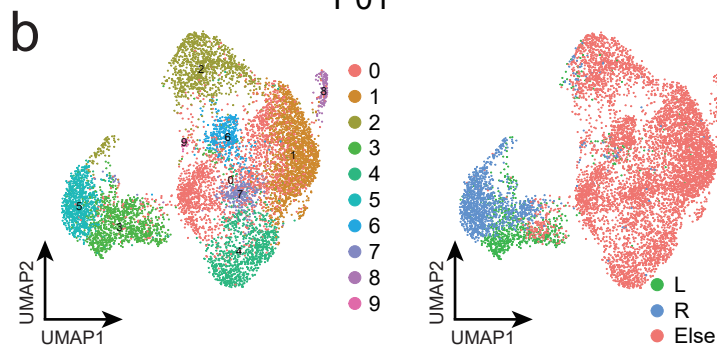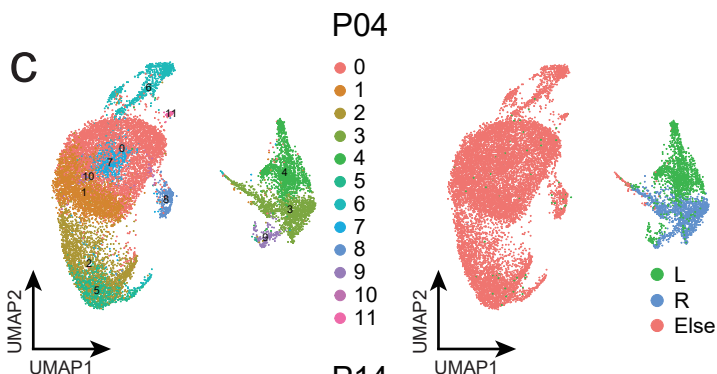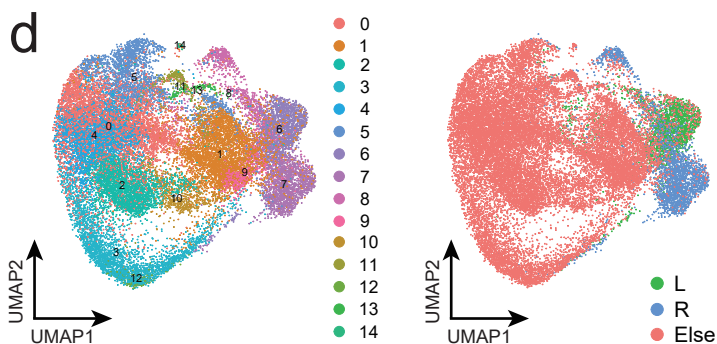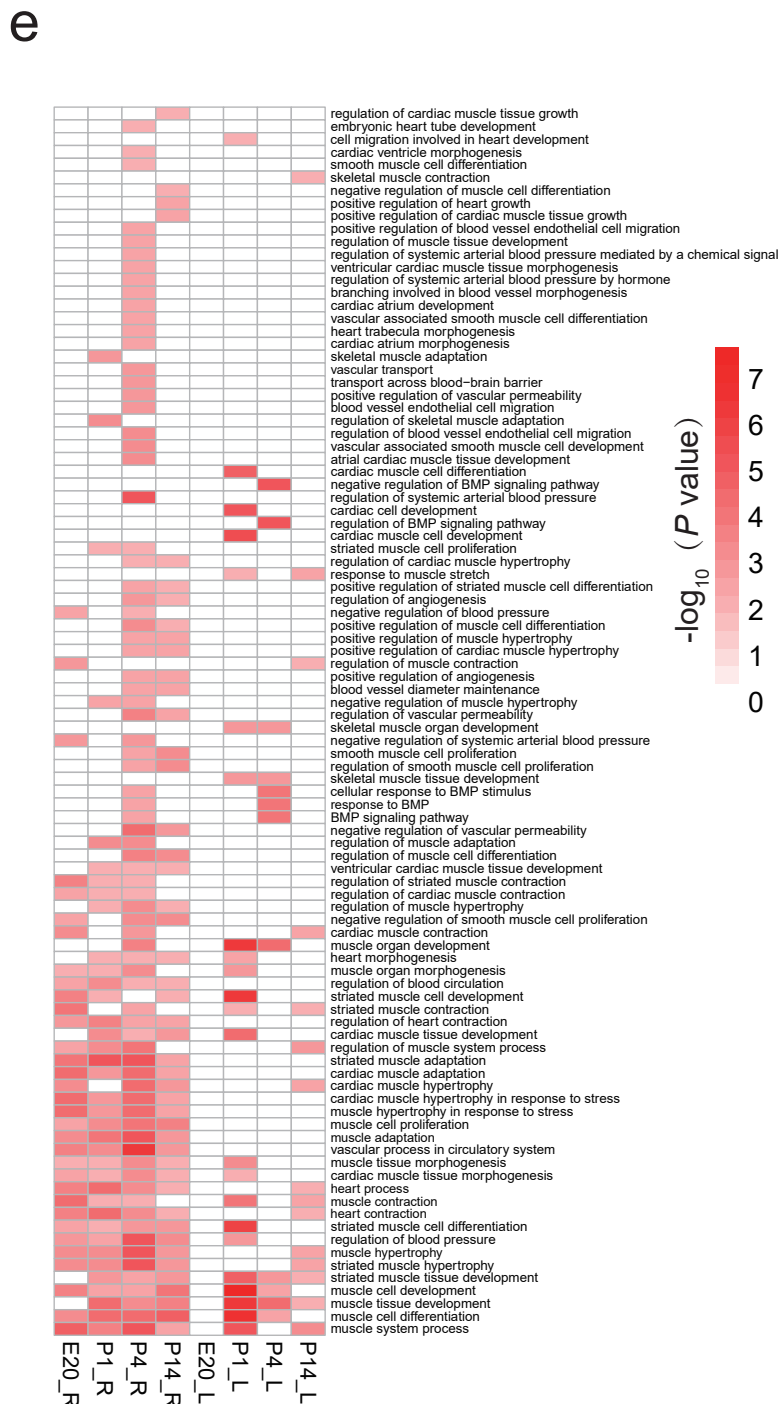

Supplement: giaf012_Supplement_Files [file giaf012_supplement_files.zip › SF7.pdf]

a

*Pitx2*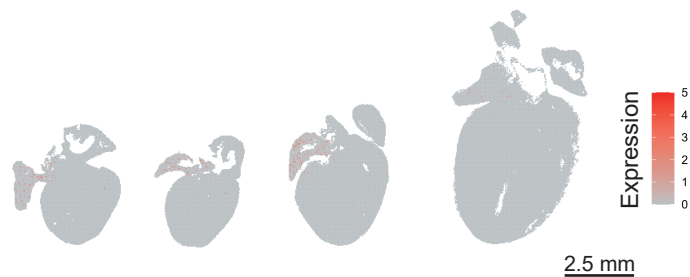

b

*Bmp10*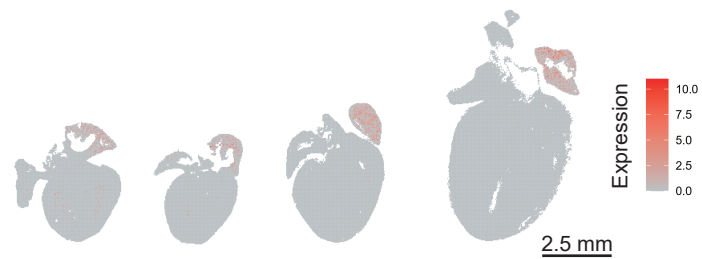

c

*Eng*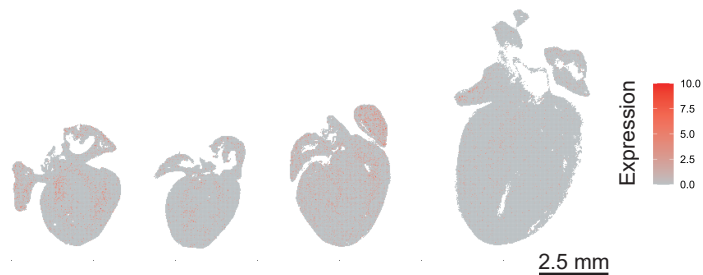

d

*Adamts8*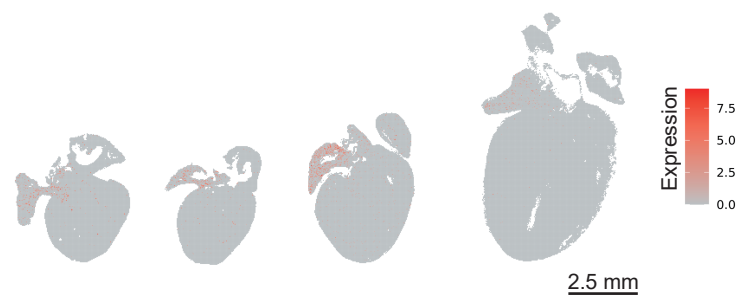

Supplement: giaf012_Supplement_Files [file giaf012_supplement_files.zip › SF8.pdf]
