# Supplementary material for: Exploring the cellular and molecular basis of murine cardiac development through spatiotemporal transcriptome sequencing
Source: Gigascience. 2025 Feb 17;14:giaf012. doi: 10.1093/gigascience/giaf012 (PMC11831923; doi:10.1093/gigascience/giaf012)
Supplement: giaf012_GIGA-D-24-00351_Original_Submission [file giaf012_giga-d-24-00351_original_submission.pdf]

GigaScience

Exploring the Cellular and Molecular Basis of Murine Cardiac Development through Spatiotemporal Transcriptome Sequencing

--Manuscript Draft--

**Manuscript Number:**	GIGA-D-24-00351
**Full Title:**	Exploring the Cellular and Molecular Basis of Murine Cardiac Development through Spatiotemporal Transcriptome Sequencing
**Article Type:**	Research
**Funding Information:**	
**Abstract:**	Spatial transcriptomics is a powerful tool that combines molecular data with spatial information, enabling a deeper understanding of tissue morphology and cellular interactions. In this study, we employed state-of-the-art spatial transcriptome sequencing technology to investigate the development of the mouse heart and establish a comprehensive spatiotemporal cell atlas of early murine cardiac development. Through the analysis of this atlas, we elucidated the spatial organization of cardiac cellular lineages and their interactions during development. Notably, we observed dynamic changes in gene expression within fibroblasts and cardiomyocytes. Furthermore, we identified critical genes including Igf2, H19, Tcap, as well as transcription factors of Tcf12 and Plagl1, that may be associated with the loss of myocardial regeneration ability during early heart development. Moreover, we successfully identified marker genes, such as Adamts8 and Bmp10, that can distinguish between the left and right atria. Our study provides novel insights into murine cardiac development and offers a valuable resource for future investigations in the field of heart research.
**Corresponding Author:**	Jingmin Kang<br>BGI-Shenzhen: BGI Group<br>Shenzhen, CHINA
**Corresponding Author Secondary Information:**	
**Corresponding Author's Institution:**	BGI-Shenzhen: BGI Group
**Corresponding Author's Secondary Institution:**	
**First Author:**	Jingmin Kang
**First Author Secondary Information:**	
**Order of Authors:**	Jingmin Kang
	Qing Song Li
	Jie Liu
	Lin Du
	Peng Liu
	Fuyan Liu
	Yue Wang
	Xunan Shen
	Xujiao Luo
	Ninghe Wang
	Renhua Wu
	Jizheng Wang
	lei Song

Powered by Editorial Manager® and ProduXion Manager® from Aries Systems Corporation

	Xin Liu
Order of Authors Secondary Information:	
Additional Information:	
Question	Response
Are you submitting this manuscript to a special series or article collection?	No
**Experimental design and statistics**<br><br>Full details of the experimental design and statistical methods used should be given in the Methods section, as detailed in our Minimum Standards Reporting Checklist. Information essential to interpreting the data presented should be made available in the figure legends.<br><br>Have you included all the information requested in your manuscript?	Yes
**Resources**<br><br>A description of all resources used, including antibodies, cell lines, animals and software tools, with enough information to allow them to be uniquely identified, should be included in the Methods section. Authors are strongly encouraged to cite Research Resource Identifiers (RRIDs) for antibodies, model organisms and tools, where possible.<br><br>Have you included the information requested as detailed in our Minimum Standards Reporting Checklist?	Yes
**Availability of data and materials**<br><br>All datasets and code on which the conclusions of the paper rely must be either included in your submission or deposited in publicly available repositories (where available and ethically appropriate), referencing such data using a unique identifier in the references and in	Yes

the “Availability of Data and Materials” section of your manuscript.<br><br>Have you have met the above requirement as detailed in our Minimum Standards Reporting Checklist?	

Powered by Editorial Manager® and ProduXion Manager® from Aries Systems Corporation

Click here to access/download;Manuscript;01 Manuscript.pdf

Exploring the Cellular and Molecular Basis of Murine Cardiac Development through Spatiotemporal Transcriptome Sequencing

Jingmin Kang[1,2,*], Qingsong Li[1,2,*], Jie Liu[3,*], Lin Du[1,4,*], Peng Liu[1], Fuyan Liu[1,2], Yue Wang[1,2,5], Xunan Shen[1,2], Xujiao Luo[1], Ninghe Wang[6], Renhua Wu[6], Jizheng Wang[7,#], Lei Song[3,7,8,#], Xin Liu[1,2,#]

[1] BGI Research, Beijing, China

[2] BGI Research, Shenzhen, China

[3] Cardiomyopathy Ward, Fuwai Hospital, National Center for Cardiovascular Disease, Chinese Academy of Medical Science and Peking Union Medical College, 167, Beilishilu, Xicheng District, Beijing 100037, China

[4] College of Life Sciences, University of Chinese Academy of Sciences, Beijing 100049, China

[5] State Key Laboratory of Quality Research in Chinese Medicine and Institute of Chinese Medical Sciences, University of Macau, Macao, China

[6] Clin Lab, BGI Genomics, Tianjin, China

[7] State Key Laboratory of Cardiovascular Disease, Fuwai Hospital, National Center for Cardiovascular Diseases, Chinese Academy of Medical Sciences and Peking Union Medical College, 167, Beilishi Road, Xicheng District, Beijing 100037, China

[8] National Clinical Research Center of Cardiovascular Diseases, Fuwai Hospital, National Center for Cardiovascular Diseases, Chinese Academy of Medical Sciences and Peking Union Medical College, 167, Beilishilu, Xicheng District, Beijing 100037, China

* These authors contributed equally.

# Correspondence should be addressed to Jizheng Wang (jzwang@hotmail.com), Lei Song (songlqd@126.com) and Xin Liu (liuxin@genomics.cn).

Abstract

Spatial transcriptomics is a powerful tool that combines molecular data with spatial information, enabling a deeper understanding of tissue morphology and cellular interactions. In this study, we employed state-of-the-art spatial transcriptome sequencing technology to investigate the development of the mouse heart and establish a comprehensive spatiotemporal cell atlas of early murine cardiac development. Through the analysis of this atlas, we elucidated the spatial organization of cardiac cellular lineages and their interactions during development. Notably, we observed dynamic changes in gene expression within fibroblasts and cardiomyocytes. Furthermore, we identified critical genes including *Igf2*, *H19*, *Tcap*, as well as transcription factors of *Tcf12* and *Plagl1*, that may be associated with the loss of myocardial regeneration ability during early heart development. Moreover, we successfully identified marker genes, such as *Adamts8* and *Bmp10*, that can distinguish between the left and right atria. Our study provides novel insights into murine cardiac development and offers a valuable resource for future investigations in the field of heart research.

Background

Single-cell sequencing has emerged as a crucial tool for investigating the cellular and molecular aspects of heart and cardiac diseases. This approach enables the identification of previously unknown cell types, as well as the characterization of gene expression patterns and regulatory networks in single cell level[1-4]. Through the use of single-cell sequencing, researchers have made significant progress in

understanding various cardiac diseases, including heart failure[5,6], arrhythmia[7], and cardiomyopathy[8,9]. These advancements have provided novel insights into disease mechanisms and potential therapeutic targets. However, despite these achivements, there is still a lack of comprehensive understanding regarding the cellular and molecular features of the heart during cardiac development and diseases. This knowledge gap is primarily due to technical limitations inherent in single-cell sequencing, especially the absence of spatial information and biases associated with different cell types[10]. Therefore, it is crucial to explore the spatial organization of heart cells and utilize this spatial information to comprehensively investigate the cellular basis of cardiac development and diseases.

Spatial transcriptomics is a rapidly advancing field that aims to integrate spatial information with transcriptomic data, allowing for the investigation of tissue organization and cellular interactions[11]. Recent advancements in spatial transcriptome technologies, such as *in situ* sequencing[12] and capture-based spatial transcriptome sequencing[13], have revolutionized the simultaneous visualization and quantification of gene expression *in situ*, eliminating the need for tissue dissociation. These innovative approaches have proven successful in studying various biological systems, including the brain[14], developing embryo[13], and disease contexts such as cancer[15] and cardiovascular disease. In the field of cardiac research, spatial transcriptomics has been employed to explore cardiac development in chicken[16], providing novel insights into the interplay between cellular differentiation and morphogenesis that underlie heart function and pathology. To comprehensively study organs like the heart, the application of spatial transcriptome technologies should be further expanded, which will enable a more comprehensive understanding of the spatial organization of cells within the heart and its implications for cardiac function and disease.

Studies on the mouse heart have demonstrated its regenerative capacity during the neonatal stage, which diminishes as it matures[17]. To gain a deeper understanding of

the mechanisms underlying neonatal heart regeneration and explore possible strategies to enhance regeneration, various approaches including genetic manipulation, tissue engineering, and stem cell therapy have been investigated. However, despite these efforts, a comprehensive understanding of these mechanisms is still lacking, highlighting the need for further research. In this study, we employed SpaTial Enhanced REsolution Omics-sequencing (Stereo-seq)[13] to construct a spatiotemporal cell atlas of developing mouse hearts. This atlas encompasses spatial transcriptome data from both regeneration-capable neonatal hearts and relatively mature hearts. By analyzing the dynamics of cellular and gene expression during heart development, we identified specific genes that may be associated with the loss of regeneration ability. Our findings provide new insights into murine cardiac development and serve as a valuable resource for future investigations in the field of heart research.

Results

Constructing a spatiotemporal atlas of mouse heart during early development

To create a comprehensive spatiotemporal transcriptomic atlas of early mouse heart development, we carefully selected four specific time points, including embryonic Day 20 (E20), postnatal Day 1 (P01), postnatal Day 4 (P04), and postnatal Day 14 (P14). Heart samples were obtained by freezing and embedding two mice at each time point. From each heart, three adjacent frozen sections were chosen from the middle region, and subjected to spatial transcriptome sequencing using the Stereo-seq technique (**Figure 1a**). In total, we obtained six spatiotemporal sections with three technical replicates for each time point, resulting in 22 high-quality sections after excluding any low-quality data. This dataset, consisting of high quality sections, represents a substantial resource for early mouse heart development (**Supplementary Figure 1a**). We then performed cell segmentation to obtain cell bins, based on a bin size of approximately 25 μm (bin 50), resulting in 330,857 cell bins in total (**Table**

S1). For these obtained cell bins, we performed gene expression-based cell clustering and utilized known marker genes to annotate the cell types. This analysis revealed the presence of 14 distinct cell types (**Figure 1b, Supplementary Figure 1b and Table S2**). By identifying differentially expressed genes, we further determined marker genes for each cell type (**Figure 1c**). To generate a single-cell transcriptomic atlas with spatial information, we mapped the annotated cell types to their respective spatial positions (**Figure 1d**). This atlas provides a visual representation of the location of each cell and the expression levels of individual genes *in situ*. Overall, we successfully constructed a comprehensive spatiotemporal transcriptomic atlas of mouse heart development.

Leveraging this constructed atlas, we conducted a detailed investigation into the cellular composition of the heart during early development. Our analysis revealed that cardiomyocytes were the predominant cell type, Cardiomyocytes constitute the primary cell type, wherein atrial cardiomyocytes (aCM) account for approximately 21% of the total cell population, and ventricular cardiomyocytes (vCM) represent approximately 57% of the overall cell proportion (**Figure 1e**). In terms of developmental dynamics, we observed an increase in the proportion of vCM over time, accompanied by a decrease in the proportion of aCM (**Figure 1e**). This suggests a major shift in the cellular composition of the heart during early development. Additionally, we identified a distinct population of fibroblasts (FB) located within the ventricle and atrium, characterized by the expression of *COL* family genes, including *Fbln5*. Interestingly, the number of these fibroblasts decreased as heart development progressed (**Figure 1e**). Furthermore, our analysis revealed a significant presence of endothelial cells, primarily situated in the middle region of the ventricle. These endothelial cells may play a crucial role in vascular development and maintenance within the heart. Lastly, we identified pericardial cells (Perd) located on the surface of the heart, which exhibited notable expression of *C3*, *Igfbp6*, and *Msln* genes (**Supplementary Figure 2a**). These pericardial cells likely contribute to the structural

integrity and protection of the heart. Collectively, our spatiotemporal transcriptomic atlas of heart development provides a comprehensive understanding of the cellular composition within the heart.

Investigating detailed cell subtypes in mouse heart and their dynamics

The spatiotemporal atlas provides a valuable subcellular-level transcriptomic dataset, offering spatial positional gene expression information of the heart. This resource enables us to delve into finer cell classification and characterization. Therefore, focusing on the three most abundant and prominent cell types, namely vCM, aCM, and FB, we conducted a comprehensive cell type annotation. By analyzing their gene expression characteristics, we successfully annotated vCM, aCM, and FB into 9, 7, and 5 distinct cell subtypes, respectively (**Figure 2a-c**). To gain further insights into these annotated subtypes, we examined their gene expression profiles and identified subtype-specific genes (**Figure 2d-f**). Leveraging these genes that exhibited specific expression patterns within each subtype, we conducted gene function enrichment analysis (**Figure 2g-i**). This analysis allowed us to uncover the functional roles and pathways associated with each cell subtype.

Based on the above cell clustering results, we further mapped these subtype cells to their physical locations in the heart **(Supplementary Figure 2b-d)**. We observed that certain subtypes exhibited obviously distinct spatial distribution characteristics, which showed consistency with their possible functions. For example, cells from one subtype of vCM (vCM4), were found to aggregate near the ventricular cavity (**Supplementary Figure 2e**), and specially expressed genes of these cells were enriched in functions related to muscle organ development, muscle system process, cardiac muscle contraction, myofibril assembly, etc.. (**Figure 2g, Table S3)** Both their spatial distribution and functional enrichment suggest vCM4 cells to be involved in the contraction and relaxation activities of the heart. Meanwhile, cells from the other subtype of aCM (aCM5), were observed to aggregate in the region of the tricuspid

and bicuspid valves (**Supplementary Figure 2f**). Enrichment of their specially expressed genes indicated their possible functions related to oxidative phosphorylation, energy derivation by oxidation of organic compounds, generation of precursor metabolites and energy, cellular respiration, respiratory electron transport chain, etc.. (**Figure 2h, Table S4**) We thus deduce that these cells may play a crucial role in providing energy for the opening and closing movements of the tricuspid and bicuspid valves, contributing to the regularity and effectiveness of cardiac pulsation.

Similarly for fibroblasts, we found cells from one subtype (FB4) to aggregate in the region of the aorta (**Supplementary Figure 2g**), with a higher proportion at E20, P01, and P04, and lower proportion at P14. We found the specially expressed genes of these cells to be enriched in functions related to cell-substrate adhesion and cardiac ventricle morphogenesis (**Figure 2i, Table S5**). This suggests FB4 cells to be associated with the construction of the cardiac cell scaffold, and this activity is more active in the early stages of heart development. While not all subtypes could be functionally annotated, this investigation allows us to gain a deeper understanding of the dynamic changes occurring within these cell subtypes throughout the developmental stages of the heart.

Cardiomyocytes mediated cell-cell interaction weakening during heart development

Cell-cell interactions play a vital role in ensuring the proper functioning of biological systems by facilitating coordination and communication between cells[18,19]. These interactions are crucial for various biological processes, including development. In addition to analyzing the cellular composition using our spatiotemporal atlas of heart development, we delved into the investigation of cell-cell interactions (**Table S6**), particularly focusing on how these interactions change throughout development. Initially, we calculated the overall number and strength of cell interactions at different

time points (**Figure 3a**). The result reflected that the number of cell-cell interactions did not significantly decrease until P14, while the strength of interactions gradually diminished over time. This suggests that during early development, the factors involved in cell-cell interactions remain intact, but the interactions weaken as development progresses. Further analysis of the pathways of interaction utilized by cells (**Figure S3a**) unveiled distinct patterns of activity at different time points. As time passes, the signals related to the construction of the cell skeleton, such as Fibronectin 1 (FN1), Laminin (LAMININ), and Collagen (COLLAGEN)[21], gradually weaken. Along with that, signals related to Insulin-like Growth Factor (IGF) cell growth[22] also gradually weaken and disappear after 14 days. This suggests that there is a certain correlation between cell growth and the construction of the cell skeleton. As the cell skeleton is completed, the cell morphology gradually becomes fixed, and probably the cells lose their regenerative ability.

In addition to examining the overall cell-cell interactions at the four stages of development, we conducted a detailed investigation into the interactions between different cell types (**Figure 3b** and **Supplementary Figure 4a-d**). Our analysis revealed that the interactions between aCM, vCM, and FB exhibited the highest strength. Further exploration of the input and output interactions of each cell type (**Figure 3c-d**) unveiled that vCM predominantly acted as the major signal-receiving cell type, while aCM served as the primary signal-output cell type, particularly during the relatively early stages (E20, P01and P04). However, at P14, in addition to the overall weakening of interactions, we observed a substantial reduction in the output strength of aCM, which was previously the main output cell type, despite vCM still receiving strong signals. This substantial reduction in interaction from aCM likely contributed significantly to the overall decrease and weakening of cell-cell interactions during cardiac maturation. Thus, we further analyzed the interaction factors present in different cell types at each of the four stages (**Figure 3e**) to elucidate the changes in cell interaction factors within aCM. Our findings indicated

that the stage-specific interaction factors neural cell adhesion molecule (NCAM) and cell adhesion molecule (CADM) at E20 were primarily present in neurons (Neur). Similarly, the stage-specific interaction factors Notch receptor (NOTCH), angiopoietin-like protein (ANGPTL), and platelet endothelial cell adhesion molecule 1 (PECAM1) at P01 were mainly observed in FB and pericytes. At P14, particularly in aCM and vCM, which were previously active in interactions, all cell interaction factors, except for COLLAGEN, were scarcely present. These results further support the notion that the decrease in cell-cell interactions during cardiac maturation can be attributed to the absence of these interaction factors. In summary, leveraging the spatiotemporal atlas of heart development, we conducted an extensive investigation into cell-cell interactions during cardiac development and identified cell-type-specific interaction factors. Notably, we discovered that a key characteristic of mouse cardiac maturation is the decrease in cell-cell interactions between aCM and vCM.

Cell trajectories revealed critical genes involved in regeneration ability loss

While human cardiac muscle cells lack regenerative capacity, mouse cardiac muscle cells possess the ability to regenerate during early development[23]. It is cruicial to understand the mechanisms underlying the loss of regenerative ability in mouse cardiac muscle cells during the developmental process. This understanding will unravel the molecular intricacies of cardiac muscle regeneration and aid future research on important heart diseases. Our heart spatiotemporal atlas of the heart encompasses four time points during early embryonic development. The first three time points correspond to the regenerative period of the mouse heart, while the final time point (P14) signifies the loss of regenerative ability[24]. To investigate the cellular and molecular changes associated with cardiac muscle regeneration, we conducted an in-depth analysis by comparing the data from the final time point with the preceding three time points. Pseudotime analysis using Monocle2 was conducted on vCM, aCM, and FB. Given that cardiomyocytes are deemed terminally differentiated cells, FB

was designated as the origin in the pseudotime trajectory (**Figure 4a** and **Supplementary Figure 5a**). This analysis revealed distinct temporal trends for these major cell types. At E20, aCM and FB exhibited high similarity compared to vCM. As time progressed, all three major cell types underwent further differentiation and by P14, the states of several cell types become consistent. The branching pattern observed among these subtypes (**Figure 4a**) indicated clear differentiation during heart development. Moreover, genes can be clustered into three cell state based on their expression changes along the trajectory (**Figure 4b, Table S7**). During the development of the heart and maturation of cardiac muscle cells, we observed notable changes in gene expression levels that correlated with the loss of regenerative capacity and the acquisition of contractile ability and cardiac function. Specifically, the expression levels of *Igf2* (Insulin Like Growth Factor 2 gene)[25,26], *H19* (a long noncoding RNA gene, H19 Imprinted Maternally Expressed Transcript)[27,28], and other genes associated with cell differentiation gradually decreased. Conversely, the expression levels of *Tcap* (Titin-Cap gene)[29], *Myh6* (Myosin Heavy Chain 6 gene)[30], *Atp2p2a2* (ATPase Sarcoplasmic, Endoplasmic Reticulum Ca^{2+} Transporting 2 gene)[31], and other genes gradually increased, indicating the acquisition of contractile ability and the initiation of cardiac function. These changes in gene expression profiles provide insights into the molecular processes underlying the transition from regenerative capacity to contractile ability during cardiac muscle development.

Transcription factors (TFs) are pivotal in regulating gene expression during various biological processes[32]. Thus we conducted a detailed investigation into the changes in transcription factors during cardiac development to identify TFs that may contribute to the loss of myocardial cell regeneration capacity (**Table S8**). Base on the regulon specificity score (RSS, **Figure 4c**), we categorized the identified TFs into two groups. The first group comprised TFs that exhibited higher activity levels in the early stages of cardiac development but gradually decreased in specificity as development progressed. In contrast, the second group consisted of TFs that displayed higher

activity levels during the later stages of cardiac maturation. Among TFs of the second group, we identified several important TFs that have been previously reported to be associated with cardiac development. These include *Wt1* (Wilms' tumor 1 gene), *Prrxl1* (Paired related homeobox 1 gene), and *Srebf1* (sterol regulatory element binding transcription factor 1). For instance, *Wt1* contains four zinc finger motifs at the C-terminus, which are crucial for DNA binding and gene activation[33]. In the context of cardiac development, *Wt1* primarily regulates processes such as epithelial-to-mesenchymal transition and angiogenesis, and it has been shown to play a critical role in early cardiac development[34]. Additionally, our analysis uncovered previously unreported TFs that may play a role in cardiac maturation, such as *Mlx* (Max-like protein X gene), which warrants further investigation. On the other hand, the TFs in the first group are more likely to be associated with the loss of myocardial cell regeneration capacity during this process. Among these, we identified *Tcf12* (transcription factor 12)[35], *Plagl1* (Pleomorphic adenoma gene-like 1), and other transcription factors that exhibited decreased activity levels with cardiac maturation.

In addition, as our analysis of differential gene expression identified *Igf2* to be possibly involved in heart development and regeneration ability loss, we further investigated whether TFs targeting the *Igf* genes exhibited expression changes associated with cardiac maturation. Speciafically, in our analysis of fibroblasts and myocardial cells, we identified six transcription factors targeting *Igf2*, including the aforementioned *Plagl1*. Notably, we observed sustained decrease in the transcription factor activity of *Plagl1* along fibroblasts and myocardial cells during cardiac development and maturation (**Figure 4d**). Furthermore, when examing its TF activity correlation with the expression of other genes (**Figure 4e**) we found that the TF *Plagl1* exhibited the strongest correlation with *Igf2*. This finding suggests that *Plagl1* may have been regulating *Igf2* during early heart development. These findings point to *Plagl1* as a potential TF involved in the differentiation and regeneration of myocardial cells. However, further comprehensive research is necessary to delve

deeper into these findings and investigate the functional roles of the identified candidate genes.

Identification of atrial asymmetry related genes

By utilizing the spatiotemporal transcriptomic atlas, we can leverage the spatial information of gene expression for molecular-level investigations. The left-right asymmetry of the heart is crucial for its functional performance[36], making it essential to understand the development of the left and right ventricles and atria. To achieve this, we first divided the left and right atrial regions based on their positions for all the Stereo-seq sections. We then validated these divisions by performing HE staining on adjacent sections (**Figure 5a-d**). This approach ensured the accuracy of our spatially defined regions. In comparison, when using the Uniform Manifold Approximation and Projection (UMAP) plot of all cells of each chip for clustering and analysis without incorporating spatial positional information, we counted the number of cells in clusters that predominantly distribute in the left/right atrium and calculated the percentage of these cells compared to the total cells in the left/right atrium. Then we found that the proportion of left atrial cells that could be directly differentiated to be lower (69%, 70%, 97%, and 91%) compared to the right atrial cells (93%, 94%, 90%, and 70%) at the four time points (**Figure 5e**, **Supplementary Figure 6a-d**, and **Table S9**). These findings highlight the importance of considering spatial information for accurate identification and analysis of left and right atrial cells.

Subsequently, following the successful distinction between left and right atrial cells, we proceeded to investigate the disparities in gene expression between these two regions. By comparing the cells of the left and right atria at four time points, we identified a set of genes that were differentially expressed (**Figure 5f**). This set comprised genes that were upregulated in the left atrium (**Figure 5g**) and genes that were upregulated in the right atrium (**Figure 5h**) at the four designated time points. Specifically, in the left atrium, we observed 17, 20, 27, and 26 upregulated genes at

E20, P01, P04, and P14, respectively. Conversely, the right atrium exhibited 23, 63, 67, and 22 upregulated genes at the corresponding time points. To gain further insights into the functional implications of these gene expression differences, we conducted a functional enrichment analysis of the identified genes **(Supplementary Figure 6e**). Notably, we found that the upregulated genes in the right atrium were significantly enriched in gene ontology (GO) terms related to heart development. In contrast, the upregulated genes in the left atrium did not exhibit substantial enrichment in any heart development-related GO terms.

We then conducted an investigation into specific genes that may be associated with atrial asymmetry. Within one of the previously mentioned enriched GO terms (GO:0003228, atrial cardiac muscle tissue development), we identified four genes, including *Pitx2*, *Bmp10*, *Eng* and *Adamts8*. *Pitx2*, a transcription factor known for its involvement in regulating left-right asymmetry development of the heart and other organs, has been established as a gene specific to the left atrium[37]. Our analysis revealed significant upregulation of *Pitx2* in the left atrium at the P01 and P04 time point (**Supplementary Figure 7a**). *Bmp10*, known as a regulatory gene of *Pitx2*, exhibited specific expression in the right atrium throughout all stages (**Supplementary Figure 7b**). Furthermore, *Eng* displayed significantly higher expression in the right atrium at the P04 stage(**Supplementary Figure 7c**). In addition, we consistently observed high expression of *Adamts8* in the left atrium throughout the entire developmental stage(**Supplementary Figure 7d**). These findings shed light on the potential involvement of these genes in atrial asymmetry and provide valuable insights into the molecular mechanisms underlying the development of the left and right atria. Further research is warranted to explore the functional roles of these genes and their contributions to atrial cardiac muscle tissue development.

Discussion

The heart plays a crucial role in our physiology, and conducting single-cell and spatial transcriptomic studies on this organ can significantly enhance our understanding of cellular and molecular changes occurring during heart development and related diseases. Such studies provide essential insights into the molecular mechanisms underlying heart-related conditions and lay the groundwork for the identification of novel targets for the treatment of heart disease[1,6,10,38,39]. In our study, we constructed a comprehensive spatiotemporal transcriptomic atlas of early mouse heart development. We examined the changes occurring at the cellular and gene levels throughout the developmental process, including alterations in cell type composition, intercellular communication, shifts in cardiac cell types during development, and the identification of candidate genes crucial for cardiac development. Our findings largely align with previous single-cell studies conducted in mice, validating the reliability of our spatiotemporal atlas. For instance, we identified the transcription factor *Wt1* as potentially playing crucial role in mouse heart development, which has already been reported in a previous mouse single-cell study[39]. However, our spatiotemporal atlas allowed us to systematically describe the cellular and gene-level changes that occur during early heart development, providing a more comprehensive understanding of the process. Moreover, we identified novel genes that may be associated with heart development. Utilizing the spatial information available in our atlas, we compared the cell types present in the left and right atria and identified genes with specific expression patterns throughout development. This analysis deepened our understanding of heart asymmetry and shed light on the molecular underpinnings of this phenomenon. Notably, our study focused on the unique aspects of mouse heart development in the early stages, distinguishing it from previously published spatiotemporal atlases of chicken heart development[16]. We specifically explored cell type changes and potential genes related to regeneration, which are distinctive to the early developmental stage of the mouse heart.

Cardiac regeneration in mice has been a prominent focus of heart research, with previous studies identifying molecules and genes associated with early cardiac regeneration in mice[40-43]. However, the mechanisms underlying myocardial cell regeneration specific to early mouse hearts, which are absent in later stages and other mammals, remain poorly understood. In our study, we conducted a comparative analysis of spatiotemporal transcriptomic data from early and late stages of mouse hearts, leading us to identify *Igf2* and the transcription factor *Plagl1*, which regulates *Igf2*, as potential contributors to early mouse cardiac regeneration. Previous studies have suggested the involvement of *Igf2* in the regeneration of the heart and other tissues in mice[44-46], while other studies, including a recent preprint article, have described the role of *Plagl1* in retinal regeneration in mice[47,48]. Notably, *Plagl1* has been shown to have inhibitory effects on cell proliferation and act as a tumor suppressor in humans[49,50]. Additionaly, *Plagl1* is an imprinted gene, exhibiting specific expression of the paternal allele in multiple tissues and being implicated in the pathogenesis of congenital heart disease[51]. Given that *Plagl1* regulates numerous downstream genes, the regulatory pathway from *Plagl1* to *Igf2* and its impact on early myocardial cell regeneration remain unclear. Our study highlights the significance of investigating the *Plagl1*-*Igf2* regulation pathway for further research on myocardial cell regeneration in mice and even humans, laying the foundation for subsequent mechanistic studies.

Our study constructed a spatiotemporal transcriptomic atlas of early mouse heart development and proposed cellular and molecular changes related to mouse heart development based on the analysis of the atlas. However, establishing an atlas that covers more time points, conducting further research on heart regeneration and heart asymmetry-related studies, and performing functional validation and mechanistic analysis are potential future directions based on our study.

Methods

Experimental animal mice and heart sample preparation

The Institutional Animal Care and Use Committee of BGI thoroughly reviewed and granted approval for the animal experimental protocol. All procedures pertaining to mouse experiments in this study strictly adhered to the ethical regulations and guidelines outlined in the Animal Experimentation protocols of BGI, in addition to compliance with the Guidelines for the Care and Use of Laboratory Animals in China (License Number BGI-IRB A21030-T1). All mice were housed in standard SPF conditions with temperatures of 65–75 °F (~18–23 °C) and with 40–60% humidity. In this study, male C57BL/6 mice at embryonic day 20 (E20), postnatal day 1 (P1), postnatal day 4 (P4), and postnatal day 14 (P14) were used; 2 mice were used for each experiment.

Anesthetize mice with chloral hydrate, then extract the hearts and place them in pre-cooled 1x PBS. Thoroughly clean the heart surfaces, ensuring the removal of blood. Employ gauze to absorb any excess 1x PBS from the heart surfaces. Subsequently, position the hearts in a dish on ice, awaiting subsequent processing steps, such as embedding.

Stereo-seq sample and library preparation

Dissect the hearts of mice at four time points from the thoracic cavity, remove the pericardium using fine forceps, rinse the surface and intracardiac blood in cold PBS, blot dry with gauze, and finally embed the hearts in Tissue-Tek O.C.T. Compound (Sakura, 4583) and rapidly freeze them on dry ice.

The stereo-seq libraries were prepared using Stereo-seq[13]. Briefly, Longitudinally section the OCT-embedded heart into 10 mm thick slices using a Leica CM1950 cryostat. These slices were then adhered to the Stereo-seq chip. The chip was completely immersed in methanol at -20 degrees Celsius for 30 minutes. Afterward, the tissue was fixed in methanol at -20°C for 30 minutes. Subsequently, the tissue

sections affixed to the chip underwent permeabilization (6 minutes for E20 and P01, 7 minutes for P04, 9 minutes for P14). Probes on the chip, equipped with coordinate tags, captured the polyA RNA released in situ from the tissue sections. Following this step, the captured polyA RNA underwent in situ reverse transcription, resulting in the synthesis of cDNA with coordinate tags. Subsequently, after tissue digestion to eliminate any residual sliced tissue from the chip, cDNA with coordinate tags was liberated using release enzymes and recovered utilizing magnetic beads. Following cDNA amplification, 20ng of cDNA were employed for fragmentation and additional amplification to finalize library construction. Ultimately, sequencing was executed using the MGI DNBSEQ sequencing platform.

Hematoxylin and Eosin Staining (HE)

The HE staining was performed following standard protocols[52]. 10 μm OCT frozen sections were mounted on glass slides and fixed in 4% paraformaldehyde in 1x PBS at room temperature for 10 minutes. Subsequently, the sections were incubated in Hematoxylin for 7 minutes, washed in Nuclease-free water, incubated in Eosin for 2 minutes, and washed again in Nuclease-free water. After air-drying at room temperature, bright-field images were captured using a Motic fluorescence microscope.

Binning data of spatial stereo-seq data

The Stereo-seq transcriptomic data in this study underwent processing using the Stereo-seq Analysis Workflow (SAW) software suite (https://github.com/BGIResearch/SAW). This suite facilitated the mapping of sequencing reads onto tissue sections, allowing for the quantitative assessment of gene expression levels at each spatial position (Spot). Typically, the “bin_size” parameter is configured to group nanochannels within a specific range into a bin unit. Through a statistical analysis of cell and bin sizes across all tissues, we selected an

individual bin size of 50 (~25 μm) as the fundamental unit for downstream analysis of the transcriptomic data generated by Stereo-seq in mouse hearts[13]. This ensured that each unit contained a sufficient number of genes to represent its molecular characteristics. Moreover, in the data quality control phase, we excluded bin 50 units with low gene counts. By examining the gene count distribution curve for each bin 50, we observed that low-quality bin 50 units tended to cluster into a small peak, and we determined the filtering threshold as the minimum value on the right side of this peak.

Cell clustering and annotation

The downstream analysis of the mouse heart transcriptomic data primarily involved the Seurat software package (version 4.1.1)[53,54]. Initially, we utilized the "SCTransform" function for each spatial section data normalization, scaling, and integration across distinct datasets from various time points. Then e integrated the Seurat objects of each chip for downstream analysis. Subsequently, PCA reduction and UMAP embedding were applied for dimensionality reduction. The "FindNeighbors" function (utilizing the top 30 principal components) performed cell clustering, followed by the "FindClusters" function for graph-based clustering. Marker genes for different clusters were identified using the "FindAllMarkers" function in Seurat with parameters set to (min.pct=0.1, logfc.threshold=0.25), and a filter based on pvalue.adj < 0.05 was applied. Cell clusters were determined using established cell type-specific markers for each cluster. For a more nuanced categorization of cell types, bins associated with a particular cell type, or groups comprising related cell types, underwent additional clustering and annotation. The subcluster annotation process for a specific cell type mirrors the description provided earlier for general cell types.

Cell chat (Ligand-receptor analysis)

We utilized the CellChat package (version 1.5.0)[55] for the inference, analysis, and visualization of the cell-cell communication network in the mouse heart transcriptomic dataset. Initially, we created a new CellChat object for each time point using the CellChatDB.mouse database within the Seurat framework. This database comprises 2021 validated molecular interactions, encompassing 60% secreted autocrine/paracrine signaling interactions, 21% extracellular matrix-receptor interactions, and 19% cell-cell contact interactions. After preprocessing the expression data by identifying over-expressed genes and interactions, we employed the 'computeCommunProb' and 'filterCommunication' functions to infer the cellular communication network and compute communication probabilities. The 'computeCommunProbPathway' function calculated communication probabilities at the signaling pathway level by summarizing probabilities associated with ligands-receptors interactions in each pathway. For an aggregated view of the cell-cell communication network, the 'aggregateNet' function was used to count links or summarize communication probabilities for each communication pair. Subsequently, the three CellChat objects for each time point were merged using the 'mergeCellChat' function for downstream analysis.

Cell differentiation inference

To facilitate subsequent trajectory analysis and transcription factor analysis, we randomly selected 500 cells from each subtype of the three cell types (FB, aCM, vCM) using the 'subset' function with the 'downsample' parameter of the SeuratObject [Satija R] package. To analyze the differentiation trajectory of mouse heart cells and investigate their pseudotime relationships, we employed the R package Monocle 2 (version 2.22.0)[56]. Initially, we used the "importCDS" function (object, import_all = F) to convert the raw counts into the CellDataSet format. Subsequently, the "estimateSizeFactors" and "estimateDispersions" functions were employed to precompute crucial parameters related to the data. The "differenceGeneTest" function

was used to select potential ordering genes (qval < 0.01), providing information for ordering cells along the pseudotime trajectory. Dimensionality reduction and clustering analysis were performed using the "reduceDimension" function (reduction_method = "DDRTree"), with "fullModelFormulaStr" set for cell type and time point. Trajectory inference was executed using the default parameters through the "orderCells" function.

Differential gene expression (DEG) analysis and GO enrichment

Differential expression analysis of the same cell type between two time points was conducted using the 'FindMarkers' function in Seurat packages. We filtered significant differentially expressed genes (DEGs) with an adjusted p-value < 0.05 and an absolute average log2 fold change > 0.5. Specifically for the identification of the left and right atria, differential analysis was performed using data from the corresponding spatial sections. The 'FindMarkers' function in the Seurat package was employed, focusing on highly variable genes based on criteria such as p-value < 0.05 and logFC > 0.5. For a deeper understanding of the functional implications of these genes, we conducted Gene Ontology (GO) enrichment analysis in the Biological Process category. This was achieved using the clusterProfiler software package (version 4.7.1)[57], with gene annotations sourced from the org.Mm.eg.db[58] annotation database for mouse loci.

Transcription factor regulation activity prediction

We utilized pySCENIC[59] for transcription factor analysis across three cell types (FB, aCM, and vCM). Initially, we downloaded the relevant cisTarget database for mice from https://resources.aertslab.org/cistarget/databases/mus_musculus/mm10. This database was constructed using the 2022 SCENIC+ motif collection, defining the search space as 500bp upstream of the transcription start site (TSS) and 100bp downstream around the TSS of the gene where the motif is scored.

We inferred potential transcription factors (TFs) and computed the transcription factor activity for each cell using the default workflow of pySCENIC (https://github.com/aertslab/pySCENIC). Subsequently, the SCENIC package (v1.3.1) [59] in R was employed to calculate the regulon specificity score of the transcription factors at different time points. The transcription factor activity was integrated with the gene expression matrix, and the correlation coefficient between the transcription factors and target genes was determined using the cor.test function from the stats (v4.3.0)[60] package.

Acknowledgements

We sincerely thank the support provided by the China National GenBank, BGI Research (CNGB). This work was supported by the CAMS Innovation Fund for Medical Sciences (2023-I2M-1-001), the National Natural Science Foundation of China (82170371 and 82300396), and the National High Level Hospital Clinical Research Funding (2023-GSP-QN-3).

Author contributions

X.L., L.S. and J.W. conceived and leaded the study. J.K., J.L., X.L., N.W. and R.W. carried out the experiments. Q.L., L.D., P.L., X.S. and F.L. analyzed the data. X.L. and J.K. wrote the manuscript. Q.L., L.D., J.L. and F.L. revised the manuscript. All authors read and approved the final manuscript.

Competing interests

The authors declare no conflict of interests.

Data availability

All raw data generated by this study can be found in NCBI-SRA with the accession number PRJNA1148773. Processed data have been deposited in the China National

GeneBank Database (CNGBdb) in the China National GeneBank Sequence Archive (CNSA) with the accession number STT0000062.

References

1 Skelly, D. A. *et al.* Single-Cell Transcriptional Profiling Reveals Cellular Diversity and Intercommunication in the Mouse Heart. *Cell Rep* **22**, 600-610, doi:10.1016/j.celrep.2017.12.072 (2018).

2 DeLaughter, D. M. *et al.* Single-Cell Resolution of Temporal Gene Expression during Heart Development. *Dev Cell* **39**, 480-490, doi:10.1016/j.devcel.2016.10.001 (2016).

3 Cui, Y. *et al.* Single-Cell Transcriptome Analysis Maps the Developmental Track of the Human Heart. *Cell Rep* **26**, 1934-1950 e1935, doi:10.1016/j.celrep.2019.01.079 (2019).

4 Tabula Muris, C. *et al.* Single-cell transcriptomics of 20 mouse organs creates a Tabula Muris. *Nature* **562**, 367-372, doi:10.1038/s41586-018-0590-4 (2018).

5 Martini, E. *et al.* Single-Cell Sequencing of Mouse Heart Immune Infiltrate in Pressure Overload-Driven Heart Failure Reveals Extent of Immune Activation. *Circulation* **140**, 2089-2107, doi:10.1161/CIRCULATIONAHA.119.041694 (2019).

6 Koenig, A. L. *et al.* Single-cell transcriptomics reveals cell-type-specific diversification in human heart failure. *Nat Cardiovasc Res* **1**, 263-280, doi:10.1038/s44161-022-00028-6 (2022).

7 Yuan, P. *et al.* Single-Cell RNA Sequencing Uncovers Paracrine Functions of the Epicardial-Derived Cells in Arrhythmogenic Cardiomyopathy. *Circulation* **143**, 2169-2187, doi:10.1161/CIRCULATIONAHA.120.052928 (2021).

8 Wang, L. *et al.* Single-cell reconstruction of the adult human heart during heart failure and recovery reveals the cellular landscape underlying cardiac function. *Nat Cell Biol* **22**, 108-119, doi:10.1038/s41556-019-0446-7 (2020).

9 Chaffin, M. *et al.* Single-nucleus profiling of human dilated and hypertrophic cardiomyopathy. *Nature* **608**, 174-180, doi:10.1038/s41586-022-04817-8 (2022).

10 Yamada, S. & Nomura, S. Review of Single-Cell RNA Sequencing in the Heart. *Int J Mol Sci* **21**, doi:10.3390/ijms21218345 (2020).

11 Vickovic, S. *et al.* High-definition spatial transcriptomics for in situ tissue profiling. *Nat Methods* **16**, 987-990, doi:10.1038/s41592-019-0548-y (2019).

12 Ke, R. *et al.* In situ sequencing for RNA analysis in preserved tissue and cells. *Nat Methods* **10**, 857-860, doi:10.1038/nmeth.2563 (2013).

13 Chen, A. *et al.* Spatiotemporal transcriptomic atlas of mouse organogenesis using DNA nanoball-patterned arrays. *Cell* **185**, 1777-1792 e1721, doi:10.1016/j.cell.2022.04.003 (2022).

14 Wei, X. *et al.* Single-cell Stereo-seq reveals induced progenitor cells involved in axolotl brain regeneration. *Science* **377**, eabp9444, doi:10.1126/science.abp9444 (2022).

15 Wu, L. *et al.* An invasive zone in human liver cancer identified by Stereo-seq promotes hepatocyte-tumor cell crosstalk, local immunosuppression and tumor progression. *Cell Res*, doi:10.1038/s41422-023-00831-1 (2023).

16 Mantri, M. *et al.* Spatiotemporal single-cell RNA sequencing of developing chicken hearts identifies interplay between cellular differentiation and morphogenesis. *Nat Commun* **12**, 1771, doi:10.1038/s41467-021-21892-z (2021).

17 Lam, N. T. & Sadek, H. A. Neonatal Heart Regeneration: Comprehensive Literature Review. *Circulation* **138**, 412-423, doi:10.1161/CIRCULATIONAHA.118.033648 (2018).

18 Sheikh, F., Ross, R. S. & Chen, J. Cell-cell connection to cardiac disease. *Trends in cardiovascular medicine* **19**, 182-190 (2009).

19 Belardi, B., Son, S., Felce, J. H., Dustin, M. L. & Fletcher, D. A. Cell–cell interfaces as specialized compartments directing cell function. *Nature Reviews Molecular Cell Biology* **21**, 750-764 (2020).

20 Tirziu, D., Giordano, F. J. & Simons, M. Cell communications in the heart. *Circulation* **122**, 928-937 (2010).

21 Lukjanenko, L. *et al.* Loss of fibronectin from the aged stem cell niche affects the regenerative capacity of skeletal muscle in mice. *Nature medicine* **22**, 897-905 (2016).

22 Li, P. *et al.* IGF signaling directs ventricular cardiomyocyte proliferation during embryonic heart development. *Development* **138**, 1795-1805 (2011).

23 Fine, B. & Vunjak-Novakovic, G. Heart regeneration in mouse and human: A bioengineering perspective. *Curr Opin Physiol* **14**, 56-63, doi:10.1016/j.cophys.2020.01.004 (2020).

24 Misra, A. *et al.* Characterizing neonatal heart maturation, regeneration, and scar resolution using spatial transcriptomics. *Journal of Cardiovascular Development and Disease* **9**, 1 (2021).

25 Minuto, F., Palermo, C., Arvigo, M. & Barreca, A. M. The IGF system and bone. *J Endocrinol Invest* **28**, 8-10 (2005).

26 Kang, H. *et al.* Insulin-like growth factor 2 promotes osteogenic cell differentiation in the parthenogenetic murine embryonic stem cells. *Tissue Eng Part A* **18**, 331-341, doi:10.1089/ten.TEA.2011.0074 (2012).

27 Milligan, L. *et al.* H19 gene expression is up-regulated exclusively by stabilization of the RNA during muscle cell differentiation. *Oncogene* **19**, 5810-5816, doi:10.1038/sj.onc.1203965 (2000).

28 Poirier, F. *et al.* The murine H19 gene is activated during embryonic stem cell differentiation in vitro and at the time of implantation in the developing embryo. *Development* **113**, 1105-1114, doi:10.1242/dev.113.4.1105 (1991).

29 Hayashi, T. *et al.* Tcap gene mutations in hypertrophic cardiomyopathy and dilated cardiomyopathy. *J Am Coll Cardiol* **44**, 2192-2201, doi:10.1016/j.jacc.2004.08.058 (2004).

30 van Gorp, P. R. *et al.* Sbk2, a Newly Discovered Atrium-Enriched Regulator of Sarcomere Integrity. *Circulation Research* **131**, 24-41 (2022).

31 Skogestad, J. *et al.* Disruption of Phosphodiesterase 3A Binding to SERCA2 Increases SERCA2 Activity and Reduces Mortality in Mice With Chronic Heart Failure. *Circulation* **147**, 1221-1236, doi:10.1161/circulationaha.121.054168 (2023).

32 Weidemüller, P., Kholmatov, M., Petsalaki, E. & Zaugg, J. B. Transcription factors: Bridge between cell signaling and gene regulation. *Proteomics* **21**, 2000034 (2021).

33 Duim SN, MJ Goumans and BPT Kruithof. 2016. WT1 in cardiac development and disease. In:

Wilms Tumor. van den Heuvel-Eibrink MM, ed. Brisbane, AU: Codon Publications. Crossref.

34 Moore, A. W., McInnes, L., Kreidberg, J., Hastie, N. D. & Schedl, A. YAC complementation shows a requirement for Wt1 in the development of epicardium, adrenal gland and throughout nephrogenesis. *Development* **126**, 1845-1857, doi:10.1242/dev.126.9.1845 (1999).

35 Wang, S. *et al.* Tcf12 is required to sustain myogenic genes synergism with MyoD by remodelling the chromatin landscape. *Communications Biology* **5**, 1201, doi:10.1038/s42003-022-04176-0 (2022).

36 Desgrange, A., Le Garrec, J.-F. & Meilhac, S. M. Left-right asymmetry in heart development and disease: forming the right loop. *Development* **145**, dev162776 (2018).

37 Logan, M., Pagán-Westphal, S. M., Smith, D. M., Paganessi, L. & Tabin, C. J. The transcription factor Pitx2 mediates situs-specific morphogenesis in response to left-right asymmetric signals. *Cell* **94**, 307-317 (1998).

38 Li, W. *et al.* Single-cell RNA-seq of heart reveals intercellular communication drivers of myocardial fibrosis in diabetic cardiomyopathy. *Elife* **12**, doi:10.7554/eLife.80479 (2023).

39 Feng, W. *et al.* Single-cell transcriptomic analysis identifies murine heart molecular features at embryonic and neonatal stages. *Nat Commun* **13**, 7960, doi:10.1038/s41467-022-35691-7 (2022).

40 Tan, Y., Duan, X., Wang, B., Liu, X. & Zhan, Z. Murine neonatal cardiac B cells promote cardiomyocyte proliferation and heart regeneration. *NPJ Regen Med* **8**, 7, doi:10.1038/s41536-023-00282-7 (2023).

41 Nakada, Y. *et al.* Hypoxia induces heart regeneration in adult mice. *Nature* **541**, 222-227, doi:10.1038/nature20173 (2017).

42 Han, C. *et al.* Acute inflammation stimulates a regenerative response in the neonatal mouse heart. *Cell Res* **25**, 1137-1151, doi:10.1038/cr.2015.110 (2015).

43 Bassat, E. *et al.* The extracellular matrix protein agrin promotes heart regeneration in mice. *Nature* **547**, 179-184, doi:10.1038/nature22978 (2017).

44 Shen, H. *et al.* Mononuclear diploid cardiomyocytes support neonatal mouse heart regeneration in response to paracrine IGF2 signaling. *Elife* **9**, doi:10.7554/eLife.53071 (2020).

45 Bella, P. *et al.* Blockade of IGF2R improves muscle regeneration and ameliorates Duchenne muscular dystrophy. *EMBO Mol Med* **12**, e11019, doi:10.15252/emmm.201911019 (2020).

46 Near, S. L., Whalen, L. R., Miller, J. A. & Ishii, D. N. Insulin-like growth factor II stimulates motor nerve regeneration. *Proc Natl Acad Sci U S A* **89**, 11716-11720, doi:10.1073/pnas.89.24.11716 (1992).

47 Garcia-Garcia, D., Locker, M. & Perron, M. Update on Muller glia regenerative potential for retinal repair. *Curr Opin Genet Dev* **64**, 52-59, doi:10.1016/j.gde.2020.05.025 (2020).

48 Touahri, Y. et al. Plagl1 is part of the mammalian retinal injury response and a critical regulator of Müller glial cell quiescence. bioRxiv, 2021

49 Liang, X. *et al.* PLAGL1 is associated with prognosis and cell proliferation in pancreatic adenocarcinoma. *BMC Gastroenterol* **23**, 2, doi:10.1186/s12876-022-02609-y (2023).

50 Sievers, P. *et al.* Recurrent fusions in PLAGL1 define a distinct subset of pediatric-type supratentorial neuroepithelial tumors. *Acta Neuropathol* **142**, 827-839, doi:10.1007/s00401-021-02356-6 (2021).

51 Zhao, X. *et al.* Imprinting aberrations of SNRPN, ZAC1 and INPP5F genes involved in the pathogenesis of congenital heart disease with extracardiac malformations. *J Cell Mol Med* **24**, 9898-9907, doi:10.1111/jcmm.15584 (2020).

52 Asp, M. *et al.* A spatiotemporal organ-wide gene expression and cell atlas of the developing human heart. *Cell* **179**, 1647-1660. e1619 (2019).

53 Hao, Y. *et al.* Integrated analysis of multimodal single-cell data. *Cell* **184**, 3573-3587. e3529 (2021).

54 Stuart, T. *et al.* Comprehensive integration of single-cell data. *Cell* **177**, 1888-1902. e1821 (2019).

55 Jin, S. CellChat: Inference and analysis of cell-cell communication from single-cell transcriptomics data. *R package version* **1** (2022).

56 Qiu, X. *et al.* Single-cell mRNA quantification and differential analysis with Census. *Nature methods* **14**, 309-315 (2017).

57 Wu, T. *et al.* clusterProfiler 4.0: A universal enrichment tool for interpreting omics data. *The innovation* **2** (2021).

58 Carlson, M., Falcon, S., Pages, H. & Li, N. org. Mm. eg. db: Genome wide annotation for Mouse. *R package version* **3**, 10.18129 (2019).

59 Aibar, S. *et al.* SCENIC: single-cell regulatory network inference and clustering. *Nature methods* **14**, 1083-1086 (2017).

60 R Core Team, R. R: A language and environment for statistical computing. (2013).

Figure legend

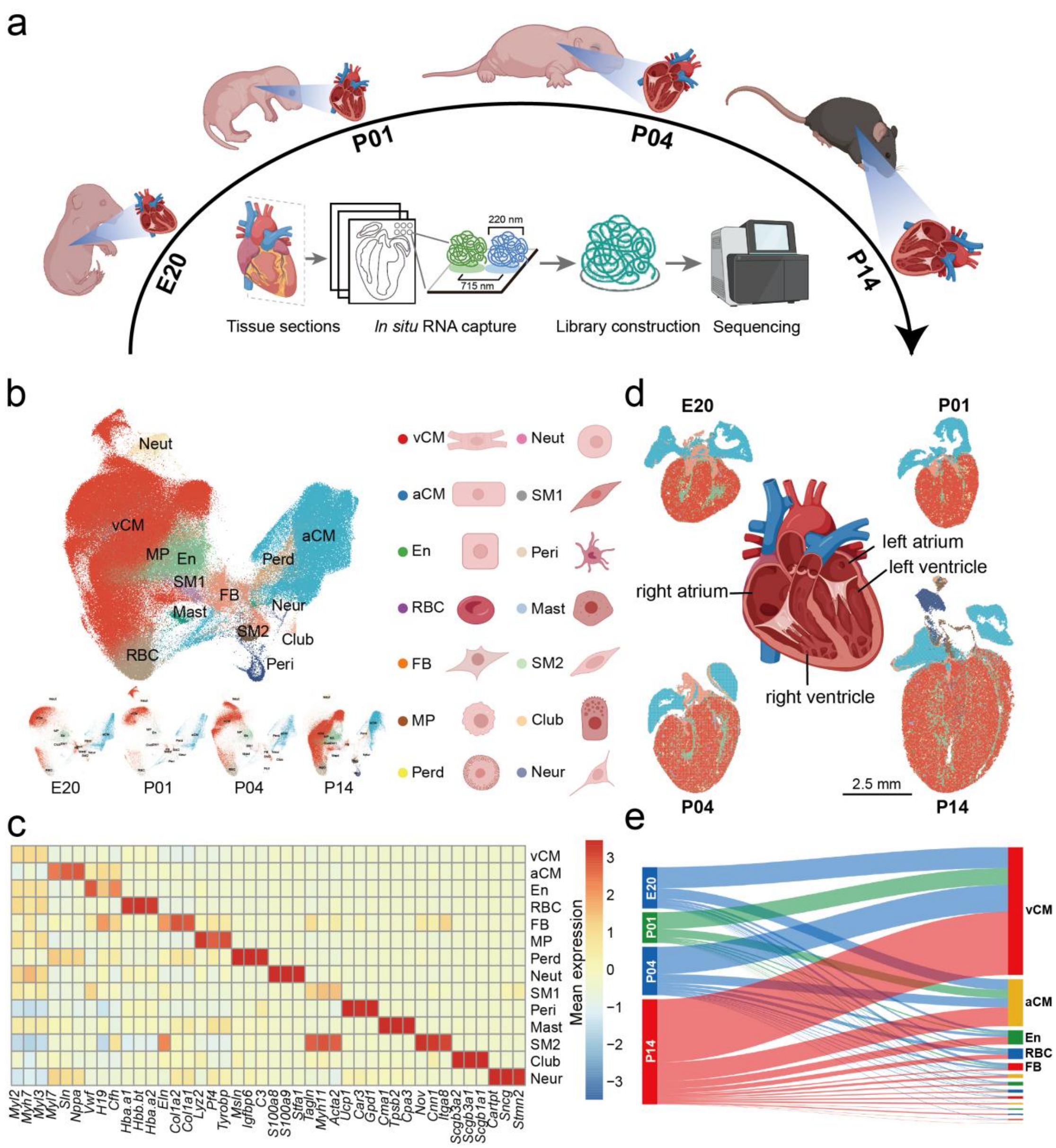

Figure 1 Spatial and temporal atlas of mouse heart development.

a. Mouse heart sampling and stereo-seq protocol. **b.** Uniform manifold approximation and projection (UMAP) of stereo-seq clustering and annotation of 22 spatial mouse chips. **c.** Heatmap of the expression of the top 3 marker genes for each cell type. **d.** Spatial and temporal distribution of mouse heart cells. **e.** Proportional changes of different cell types at four time points.

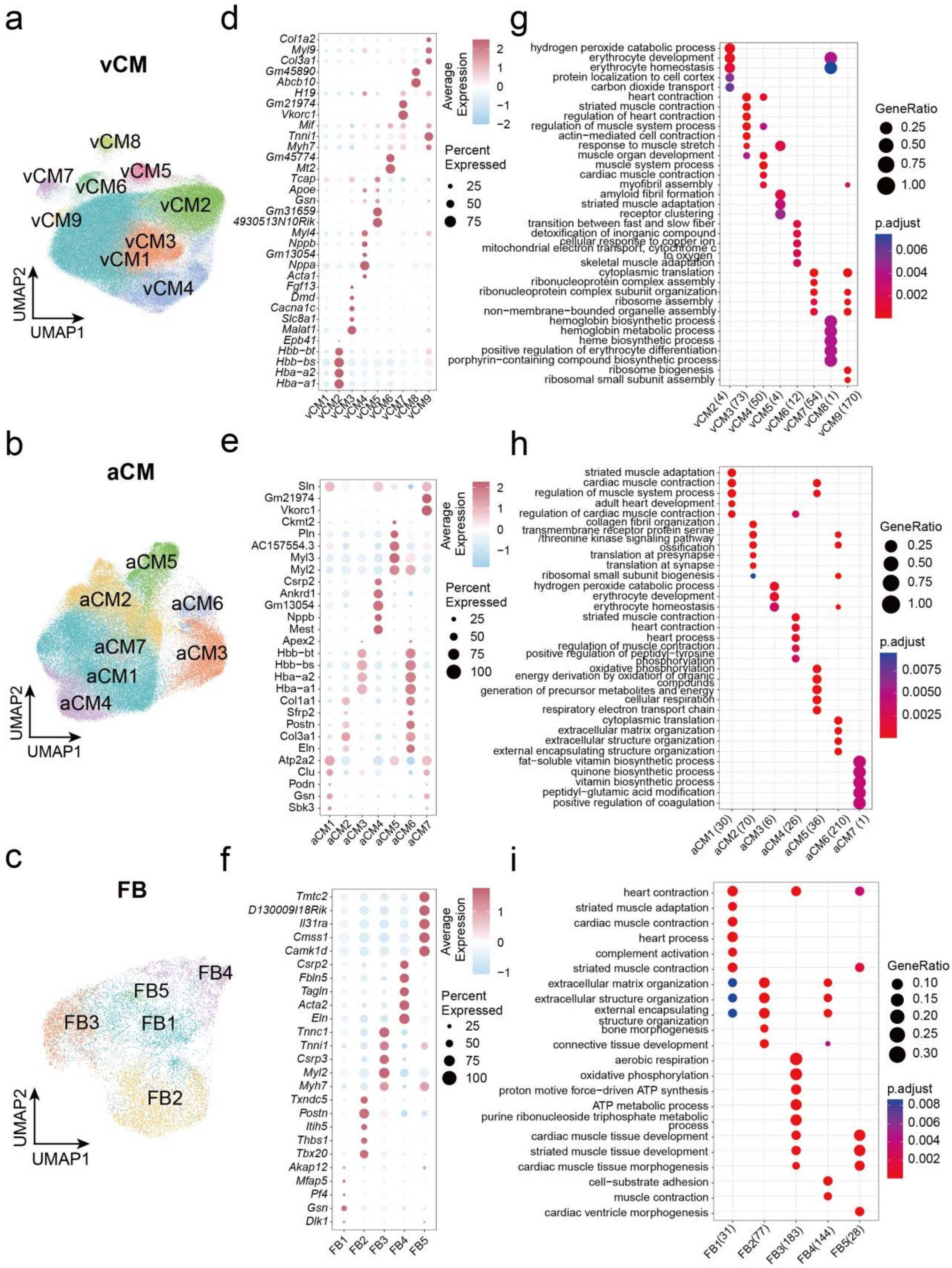

Figure 2 Classification of cell subtypes.

a-c. Subtype clustering of vCM, aCM, and FB. **d-f.** Expression of the top five marker genes in vCM, aCM, and FB. **g-i.** Enrichment of the top five GO functions in vCM, aCM, and FB.

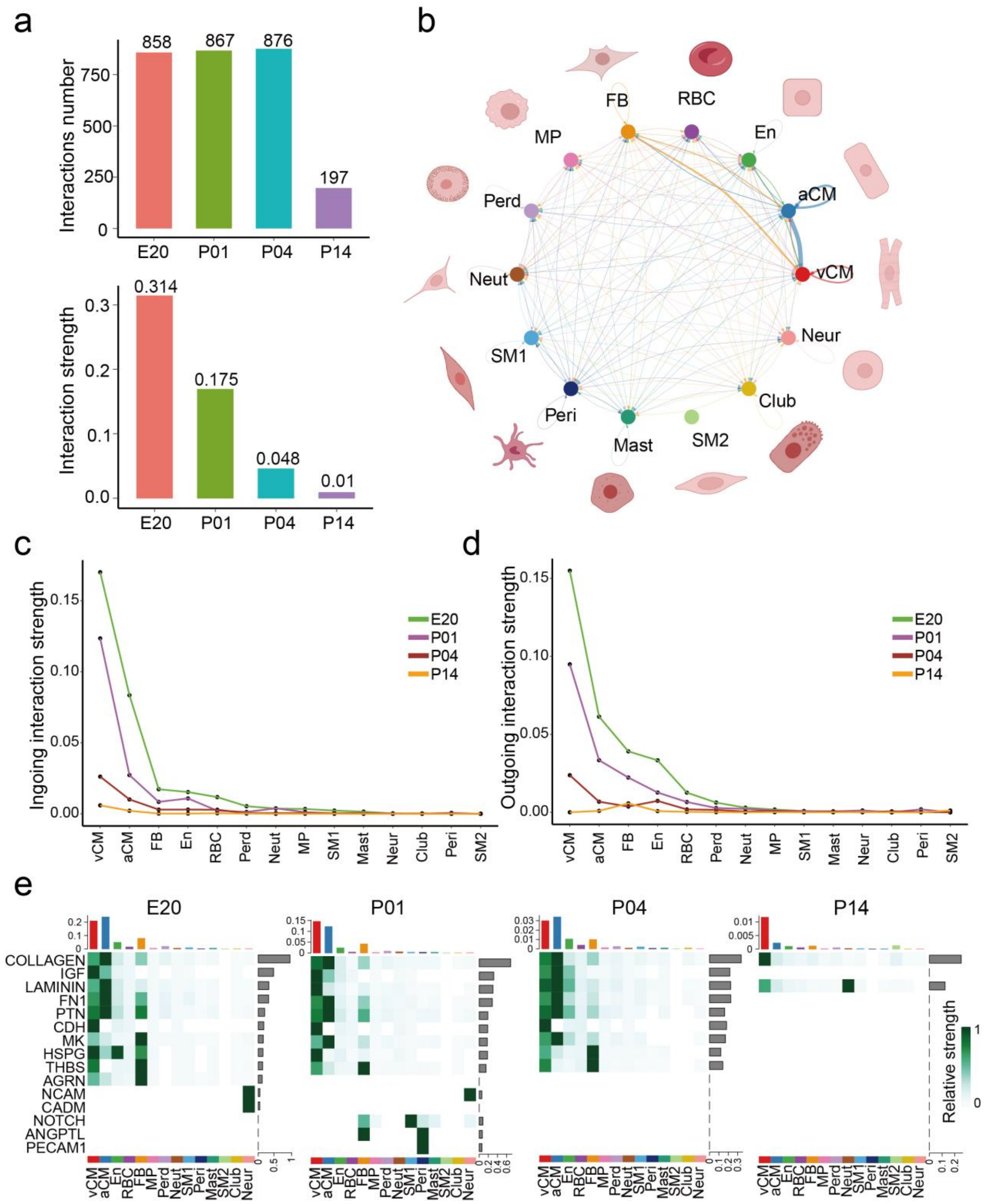

Figure 3 Cell-cell interactions of different cell types.

a. Cell interaction strength and interaction count at four time points. **b.** Cell interaction strength among cells at E20. **c.** Ingoing interaction strength of different cell types at four time points. **d.** Outgoing interaction strength of different cell types at four time points. **e.** Signal pathway strength of different cell types at four time points.

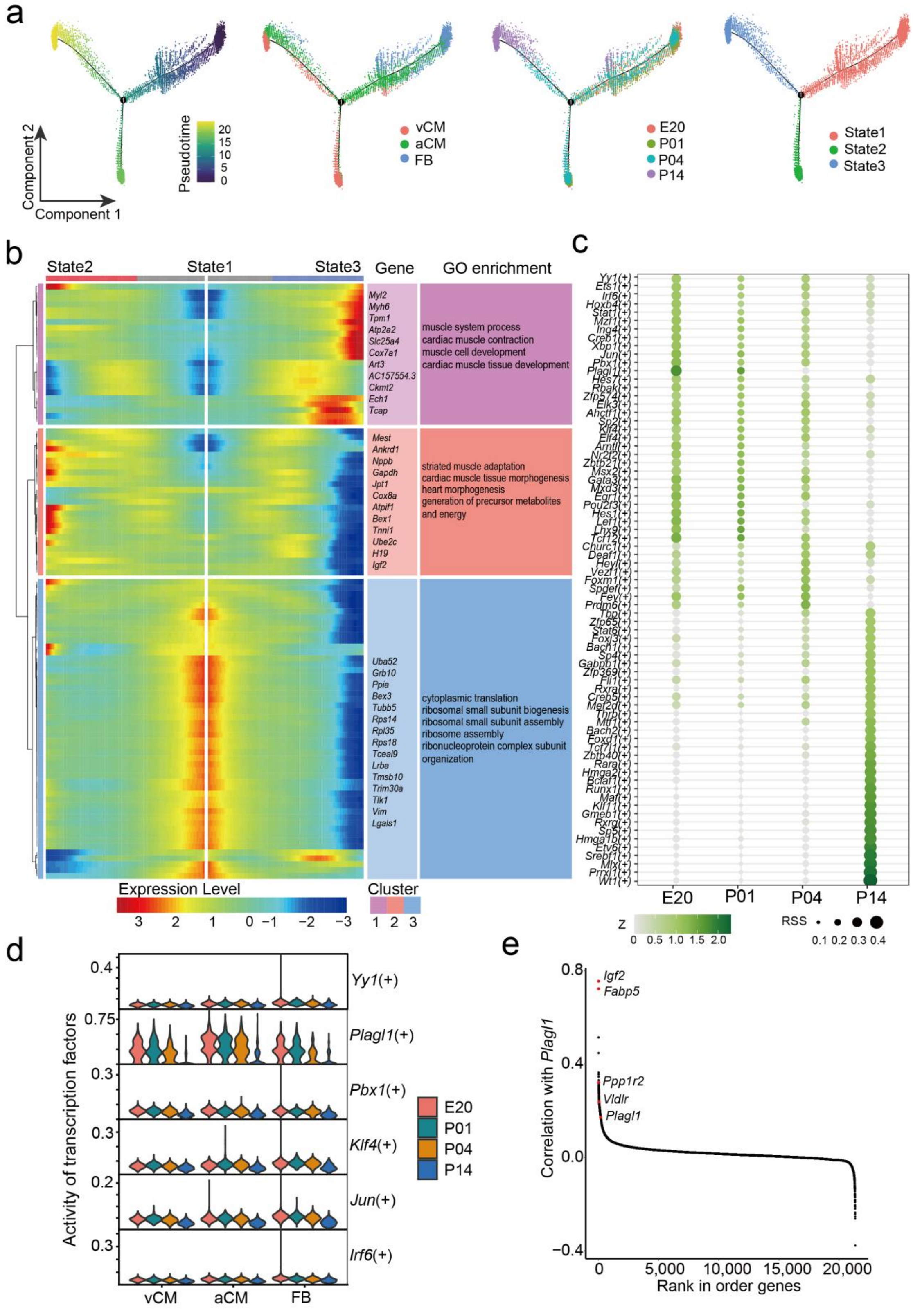

Figure 4 Trajectory analysis and transcription factor analysis.

a. Trajectory analysis of three cell types (aCM, vCM, FB). **b.** Heatmap of gene

expression changes in three cell states at branch node 1, and GO enrichment results of different clustered genes. **c.** Analysis of transcription factor activity at four time points. **d.** Changes over time in the expression of transcription factors targeting the gene *Igf2* in three different cell types. **e.** Correlation between *Plagl1* transcription factor activity and gene expression. The red dots represent target genes.

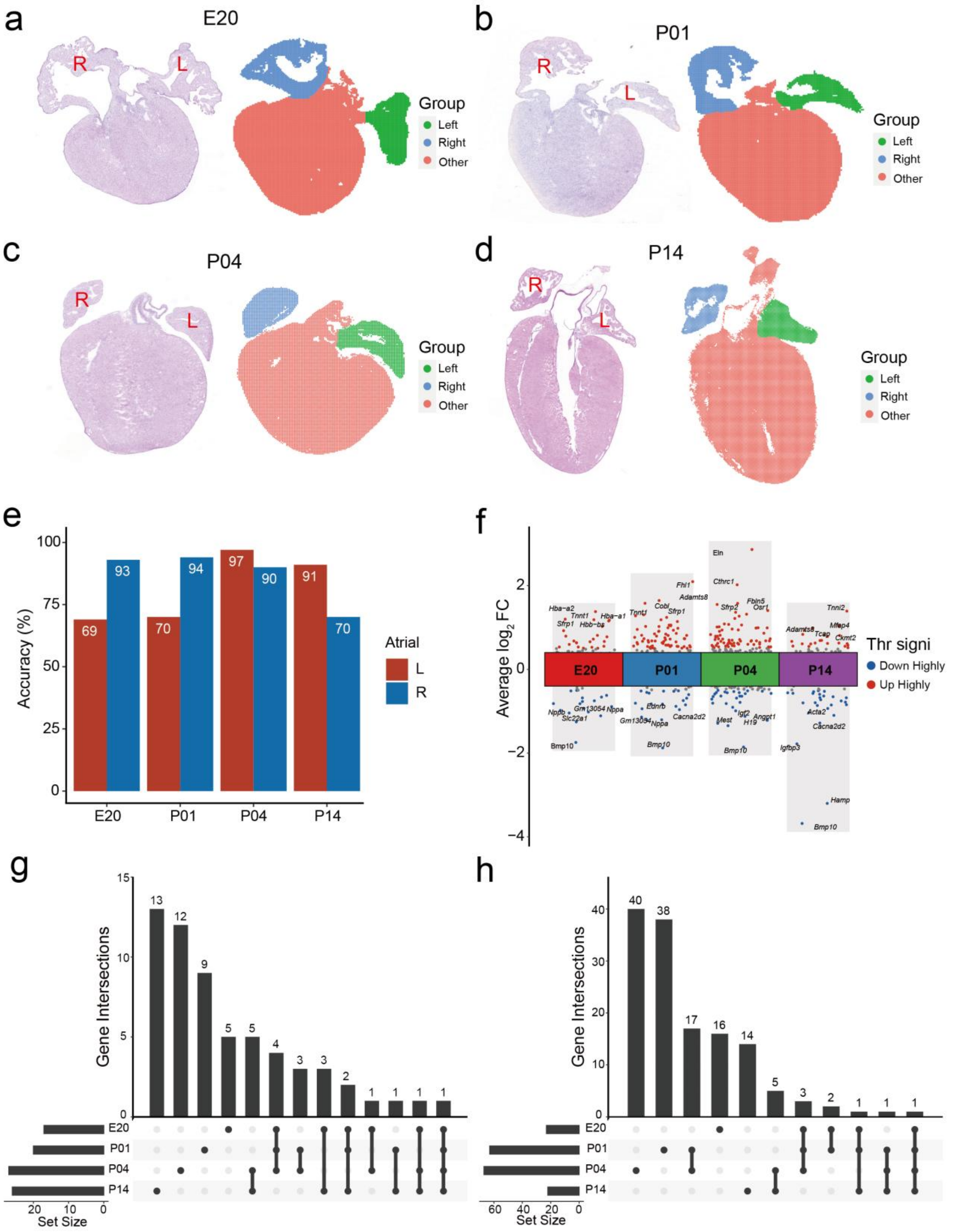

Figure 5 Identification of left and right atria.

a-d. HE sections and left and right atrium lasso at four time points. **e.** Accuracy of clustering to the left and right atria. **f.** Annotate left and right atrial differentially expressed genes. **g**. Intersection of highly variable genes in the left atrium at four time points. **h**. Intersection of highly variable genes in the right atrium at four time points.

Click here to access/download
Supplementary Material
02 Supplementary data.pdf

Supplementary Table

Click here to access/download
Supplementary Material
03 Supplementary Tables.xlsx

Personal Cover Click here to access/download;Personal Cover;04 CoverLetter.docx

Dear Editor,

This paper has passed the presub discussion by editors.

I am writing to submit our manuscript titled ***Exploring the Cellular and Molecular Basis of Murine Cardiac Development through Spatiotemporal Transcriptome Sequencing*** for consideration for publication in ***GigaScience***. I believe that our study offers a novel perspective and deeper understanding of mouse cardiac development, particularly in elucidating the spatial heterogeneity of different cell types.

While single-cell sequencing has become instrumental in understanding cardiac diseases, much remains unknown about the spatial distribution and interactions of cells. Therefore, we employed state-of-the-art spatial transcriptomic sequencing to conduct a comprehensive investigation into mouse cardiac development, successfully establishing a detailed spatiotemporal cell atlas of early mouse cardiac development. Through meticulous analysis of this atlas, we not only revealed the spatial organization of cardiac cell lineages but also explored their interactions during development.

Furthermore, we identified a series of key genes potentially associated with the loss of cardiac regenerative capacity during early cardiac development, such as *Igf2*, *H19*, and *Tcap*, as well as the transcription factors Tcf12 and Plagl1. We also successfully pinpointed some marker genes capable of distinguishing between the left and right atria, such as *Adamts8* and *Bmp10*.

I firmly believe that this study provides valuable new resources and insights for the field of mouse cardiac development. I am confident that these findings will attract a wide readership and have a profound scientific impact on the cardiovascular field, and thus is suitable for publication in ***GigaScience***.

All raw data generated by this study can be found in the NCBI Sequence Read Archive (SRA) under the accession number PRJNA1148773. The reviewer link for project PRJNA1148773 is available at https://db.cngb.org/stomics/project/STT0000062_48e0955a/reviewer_link/. Processed data have been deposited in the China National GeneBank Database (CNGBdb) within the China National GeneBank Sequence Archive (CNSA) under the accession number STT0000062. The reviewer link for project STT0000062 is available at https://dataview.ncbi.nlm.nih.gov/object/PRJNA1148773?reviewer=3mjhmcroqijmbco6og724gfmbm/.

We confirm that this manuscript has not been simultaneously submitted to any other journal and is not under consideration elsewhere for publication. All authors have read and approved the final version of the manuscript, and there is no conflict of interest to disclose.

Thank you for considering our manuscript for publication. We appreciate your time and

look forward to hearing from you soon.

Sincerely,

Jingmin Kang
BGI Research Beijing Institute
kangjingmin@genomics.cn
