# Supplementary material for: Exploring the cellular and molecular basis of murine cardiac development through spatiotemporal transcriptome sequencing
Source: Gigascience. 2025 Feb 17;14:giaf012. doi: 10.1093/gigascience/giaf012 (PMC11831923; doi:10.1093/gigascience/giaf012)
Supplement: giaf012_GIGA-D-24-00351_Revision_1 [file giaf012_giga-d-24-00351_revision_1.pdf]

GigaScience

Exploring the Cellular and Molecular Basis of Murine Cardiac Development through Spatiotemporal Transcriptome Sequencing

--Manuscript Draft--

**Manuscript Number:**	GIGA-D-24-00351R1
**Full Title:**	Exploring the Cellular and Molecular Basis of Murine Cardiac Development through Spatiotemporal Transcriptome Sequencing
**Article Type:**	Research
**Funding Information:**	
**Abstract:**	Spatial transcriptomics is a powerful tool that combines molecular data with spatial information, enabling a deeper understanding of tissue morphology and cellular interactions. In this study, we employed state-of-the-art spatial transcriptome sequencing technology to investigate the development of the mouse heart and establish a comprehensive spatiotemporal cell atlas of early murine cardiac development. Through the analysis of this atlas, we elucidated the spatial organization of cardiac cellular lineages and their interactions during development. Notably, we observed dynamic changes in gene expression within fibroblasts and cardiomyocytes. Furthermore, we identified critical genes including Igf2, H19, Tcap, as well as transcription factors of Tcf12 and Plagl1, that may be associated with the loss of myocardial regeneration ability during early heart development. Moreover, we successfully identified marker genes, such as Adamts8 and Bmp10, that can distinguish between the left and right atria. Our study provides novel insights into murine cardiac development and offers a valuable resource for future investigations in the field of heart research.
**Corresponding Author:**	Jingmin Kang<br>BGI-Shenzhen: BGI Group<br>Shenzhen, CHINA
**Corresponding Author Secondary Information:**	
**Corresponding Author's Institution:**	BGI-Shenzhen: BGI Group
**Corresponding Author's Secondary Institution:**	
**First Author:**	Jingmin Kang
**First Author Secondary Information:**	
**Order of Authors:**	Jingmin Kang
	Qing Song Li
	Jie Liu
	Lin Du
	Peng Liu
	Fuyan Liu
	Yue Wang
	Xunan Shen
	Xujiao Luo
	Ninghe Wang
	Renhua Wu
	Jizheng Wang
	lei Song

Powered by Editorial Manager® and ProduXion Manager® from Aries Systems Corporation

	Xin Liu
**Order of Authors Secondary Information:**	
**Response to Reviewers:**	Reviewer1:<br>The manuscript presents a well-executed investigation into the dynamic changes occurring in the murine heart during four developmental stages, utilizing state-of-the-art Stereo-seq technology. The study reveals shifts in cardiac cell populations, such as the increase in certain cardiomyocyte subtypes and the decrease in others, and explores molecular mechanisms that play a role in the loss of regenerative capacity, heart asymmetry, and weakened intercellular interactions. The spatial transcriptomics data provided are of significant value, and the authors adeptly highlight the key findings. However, there are a number of major and minor issues that require attention to strengthen the manuscript.<br><br>Major Concerns:<br>1. Inclusion of Earlier Embryonic Stages:<br>While E20 is included, this stage is close to the postnatal period and may miss important transitions during earlier embryonic development. Incorporating time points such as E9.5, E12.5, E15.5, and E17.5 would provide a more complete picture of the heart's developmental trajectory and would greatly enhance the study's ability to capture critical phases of embryogenesis. The author may provide additional information using other public spatial transcriptomics data sets from earlier stages, such as those in Chen et al. 2020.<br>Response:<br>We appreciate your insightful suggestions and agree that incorporating transitions during earlier embryonic development would be beneficial. We have collected spatial transcriptomics data from early hearts at E9.5, E10.5, E11.5, and E12.5 stages as reported by Chen et al. (DOI: 10.1126/science.abg5159) and have conducted the following additional analyses:<br>(1) Since Chen et al. did not isolate cardiac cells specifically, we clustered the entire mouse embryo section data (Supplementary figure 6a), annotated cardiomyocytes using vCM markers (Myh12, Myl3, Myh7) and aCM markers (Myh14, Myl7, Nppa), and found that cardiac cells were located in clusters 8 and 16 (Supplementary figure 6b). However, cardiomyocytes in the mouse embryo are still undifferentiated at this stage, making it impossible to distinguish between aCM and vCM.<br>(2) We further counted the number of cardiomyocytes at four time points, which were 13 (E9.5), 66 (E10.5), 130 (E11.5), and 222 (E12.5), respectively, and demonstrated their spatial distribution (Supplementary figure 6c). Our results are largely consistent with the cardiomyocyte annotation by Chen et al.<br>(3) Due to the small number of early embryonic cardiomyocytes and the inability to differentiate specific cell subtypes, we did not include them in trajectory analysis. Instead, we presented key genes and transcription factors involved in cardiomyocyte differentiation (Supplementary figure 6d). We observed high expression of regeneration-related genes Igf2, H19, and transcription factor Plagl1, while transcription factors Wt1, Prrxl1, Srebf1 were lowly expressed. Genes related to myocardial contraction such as Tcap, Myh6, Atp2a2 showed an upward trend, consistent with the trends of cardiomyocyte differentiation before and after birth in mice (figure 4b-c).<br><br>2. Consistency in Cell Typing Across Figures:<br>Inconsistencies are noted in the cell subtyping presented in different figures. For instance, while Figure 2 provides detailed subtyping of ventricular and atrial cardiomyocytes (vCM, aCM) and fibroblasts (FB), this level of resolution is absent in Figures 3 and 4. Consistent cell subtyping across all figures is essential for accurately conveying the developmental trajectories and interactions of different cell types during heart development.<br>Response:<br>Thank you very much for the valuable suggestions from the reviewer! To make the logic of the article tighter, we have reorganized the order of Figures 2 and 3. The new figure sequence is as follows:<br>Figure 1: Spatial and temporal atlas of mouse heart development.<br>Figure 2: Cell-cell interactions of different cell types. We completed analyses of cell-cell interactions at four time points.<br>Figure 3: Classification of cell subtypes.

Powered by Editorial Manager® and ProduXion Manager® from Aries Systems Corporation

Figure 4: Trajectory analysis and transcription factor analysis.
Figure 5: Identification of left and right atria.
We first performed cell annotation on the cardiac spatial transcriptomics data (Fig. 1), then analyzed cell-cell interactions among major cell categories (Fig. 2). Noting that fibroblasts (FB), atrial cardiomyocytes (aCM), and ventricular cardiomyocytes (vCM) are the main cell types in the cardiac interaction network, we conducted subcategory annotations for these three cell types (Fig. 3). We then extracted 500 cells from each subtype to infer cell differentiation trajectories (Fig. 4). Finally, we identified specific genes for the left and right atria (Fig. 5).
We believe this new organization of figures is more logical and will assist readers in better understanding the content of each section and following the progress of the research more smoothly. Once again, thank you for your valuable feedback on the structure of the figures.

3. Experimental Validation of Key Findings:
The manuscript provides computational insights into myocardial regenerative loss and heart asymmetry, but these findings would be significantly strengthened by experimental validation. Validation of the computational predictions, especially the proposed key markers and transcription factors, would elevate the manuscript's overall impact.
Response：
Thank you very much for your valuable suggestions. We have validated the key genes potentially involved in the loss of cardiac regenerative capacity during heart development using qPCR, specifically focusing on Igf2 and Plagl1, a transcription factor that may regulate Igf2 during early heart development. Our observations show that Igf2 remains highly expressed until the heart loses its regenerative capacity, after which its expression level drops sharply. The expression trend of Plagl1 is very similar to that of Igf2, consistent with our spatial transcriptomics data. This further increases the reliability of our conclusions, which we have added to Figure 4f and Figure 4g.
Additionally, due to the characteristic of spatial location information (an increasing number of studies have begun to use spatial transcriptomics experiments as an alternative to traditional in situ hybridization experiments), we can visually observe the expression locations of left atrial and right atrial specific genes (Figure S7). Among the four marker genes that we have enriched, Pitx2 has been reported as a marker gene for the left atriuma, which is consistent with our conclusions. This consistency further increases the reliability of our results.
In summary, the integration of qPCR validation for critical genes like Igf2 and Plagl1, along with the spatial visualization of atrial-specific gene expression provided by spatial transcriptomics, significantly enhances the robustness and credibility of our findings regarding the mechanisms underlying the loss of cardiac regenerative capacity and atrial differentiation.

a. Logan, M., Pagán-Westphal, S. M., Smith, D. M., Paganessi, L. & Tabin, C. J. The transcription factor Pitx2 mediates situs-specific morphogenesis in response to left-right asymmetric signals. Cell 94, 307-317 (1998).

Minor Concerns:
1. Detail in Figure Legends:
The legends for several figures, particularly Figures 3 and 4, lack sufficient detail. For example, Figure 3 does not provide enough information regarding the parameters used in the cell-cell interaction analysis, and Figure 4 is missing specifics about the criteria used for trajectory analysis. Providing more comprehensive figure legends will make it easier for readers to interpret the data without having to repeatedly reference the main text.
Response：
We have rewritten the captions for Figures 1 through 5, paying special attention to Figures 3 and 4. In these captions, we have included more detailed explanations, such as the meanings of the axes and legends, parameters used, significance markers, abbreviations, and symbols. Furthermore, we checked and ensured that consistent terminology and formatting were used across all captions to better assist readers in understanding the data. We believe these improvements will make the captions clearer and easier for readers to comprehend.

2. Figure Readability:
The text in multiple figures is difficult to read due to low resolution and small font sizes. Enhancing the resolution and adjusting the font size and formatting would improve the readability of the figures and ensure that labels and annotations are clear.
Response：
We greatly appreciate your valuable suggestions. In response, we have saved the figures in high-resolution PDF format and provided PNG files with a resolution of 500 dpi. Moreover, we have adjusted the font sizes and formatting to enhance readability. We carefully reviewed all text within the figures, including axis labels, legends, and titles, to ensure they are clear and legible. We thank you for your attention to these details, as it has significantly improved the overall quality and clarity of the data.

3. Clarification of Figure 5's Contribution:
Figure 5 appears to add little to the overall analysis and could benefit from further explanation. It would be helpful to include a more intuitive visualization that highlights key spatial transcriptomic insights, providing a clearer understanding of the data. Improving the spatial representation would make this figure more informative and relevant.
Response：
We have added the results of unsupervised clustering for the left and right atria. This addition is intended to better illustrate the differences between the clustering outcomes and the actual conditions of the left and right atria. We believe this will enable readers to visually observe the advantages of spatial transcriptomics data with spatial location information in annotating the left and right atria.

Reviewer2:
Kang et al. utilized the highest-resolution spatial transcriptomics sequencing technology (Stereo-Seq) to map the spatial transcriptome atlas of the mouse heart across four developmental stages (E20, P01, P4, P14). This study revealed changes in cells and genes within the heart and, based on high-resolution spatial data, further subclustered vCMs, aCMs, and FBs. Subsequently, it explored changes in interactions among heart cells, possible reasons for the loss of cardiac regeneration ability, and cellular asymmetry, highlighting the importance of spatial location information. Overall, the authors ingeniously emphasized the critical role of position-specific information unique to spatial transcriptomics, providing a valuable data resource for cardiac spatial transcriptomics. However, before publication, further modifications are needed.

Major suggestions:
1. Figure 2 describes the sub-cellular types of vCM, aCM, and FB, but Figure 3 discusses the cell-cell interactions of all cardiac cells. Figure 4 then utilizes the results of sub-cellular clustering obtained from Figure 2 for pseudo-time analysis. The authors should adjust the order of Figure 2 and Figure 3 to make the article more logical.
Response:
Thank you very much for the valuable suggestions from the reviewer! To make the logic of the article tighter, we have reorganized the order of Figures 2 and 3. The new figure sequence is as follows:
Figure 1: Spatial and temporal atlas of mouse heart development.
Figure 2: Cell-cell interactions of different cell types. We completed analyses of cell-cell interactions at four time points.
Figure 3: Classification of cell subtypes.
Figure 4: Trajectory analysis and transcription factor analysis.
Figure 5: Identification of left and right atria.
We first performed cell annotation on the cardiac spatial transcriptomics data (Fig. 1), then analyzed cell-cell interactions among major cell categories (Fig. 2). Noting that fibroblasts (FB), atrial cardiomyocytes (aCM), and ventricular cardiomyocytes (vCM) are the main cell types in the cardiac interaction network, we conducted subcategory annotations for these three cell types (Fig. 3). We then extracted 500 cells from each subtype to infer cell differentiation trajectories (Fig. 4). Finally, we identified specific genes for the left and right atria (Fig. 5).
We believe this new organization of figures is more logical and will assist readers in better understanding the content of each section and following the progress of the research more smoothly. Once again, thank you for your valuable feedback on the structure of the figures.

Powered by Editorial Manager® and ProduXion Manager® from Aries Systems Corporation

2. The description of the specific source of the results in Figure 5e is not very clear. The authors need to provide a clearer description of how this result was calculated, such as adding some figures to help readers understand more intuitively.
Response:
Thank you very much for the suggestion. We have added the results of unsupervised clustering for the left and right atria in Figure 5a-d. This allows readers to visually observe the differences in annotation of the left and right atria with and without spatial location information, thereby providing a more intuitive understanding of the meaning of Figure 5e. Additionally, we have detailed in Supplementary Table 9 how we calculated the annotation accuracy presented in Figure 5e. We believe this will significantly enhance readers' comprehension of Figure 5e.

3. The manuscript identified potential key genes and transcription factors associated with the loss of myocardial regenerative capacity. These findings require experimental validation to enhance their reliability. The authors should at least demonstrate through qPCR that genes such as Igf2 and transcription factors like PLAGL1 indeed significantly decrease at P14.
Response:
Thank you very much for your valuable suggestions. We have validated the key genes potentially involved in the loss of cardiac regenerative capacity during heart development using qPCR, specifically focusing on Igf2 and Plagl1, a transcription factor that may regulate Igf2 during early heart development. Our observations show that Igf2 remains highly expressed until the heart loses its regenerative capacity, after which its expression level drops sharply. The expression trend of Plagl1 is very similar to that of Igf2, consistent with our spatial transcriptomics data. This further increases the reliability of our conclusions, which we have added to Figure 4f and Figure 4g.

Minor suggestions:
1. Several figures are illegible due to low resolution or color scheme issues. The authors need to enhance the resolution of the images to improve readability.
Response:
We greatly appreciate your valuable suggestions. In response, we have saved the figures in high-resolution PDF format and provided PNG files with a resolution of 500 dpi. Moreover, we have adjusted the font sizes and formatting to enhance readability. We carefully reviewed all text within the figures, including axis labels, legends, and titles, to ensure they are clear and legible. We thank you for your attention to these details, as it has significantly improved the overall quality and clarity of the data.

2. I suggest that the author carefully review the language of the article. When assessing the clarity of your text, note that the picture you have in your mind and the picture evoked by your writing in your readers' minds should be identical. If you are curious about whether your revised text is clear, try this exercise with a respected colleague who is not familiar with the details of this study. Note that clear text is a prerequisite for any fulsome scientific assessment.
Response:
Thank you very much for your suggestion. We understand that clear text is crucial for any comprehensive scientific assessment, and we have carefully revised the language throughout the manuscript. We believe the text is now sufficiently clear for others to understand.

3. I suggest that the author check the text to avoid basic grammatical errors, such as "(E20, POland P04)" in line 21.
Response:
We have carefully reviewed the text and corrected any grammatical errors. We have ensured grammatical accuracy and clarity to improve the quality of the article. If there are other specific suggestions for changes, please feel free to point them out.

4. Add a separator for the numbers over 1,000. Check all numbers including those in the tables/figures, such as "This database 5 comprises 2021 validated molecular interactions,…". Please check to see if there are other places with similar problems.
Response:
We have carefully reviewed all numbers in the document and have added appropriate separators to all numbers greater than 1000 as suggested by you. We have thoroughly checked the numbers in the document to ensure that we have not missed any similar

Powered by Editorial Manager® and ProduXion Manager® from Aries Systems Corporation

	problems. Thank you very much for your attention to detail, which will help improve the quality and readability of your manuscript.<br><br>5. The following sentence requires a space between the number and the unit: "This database was constructed using the 2022 SCENIC+ motif collection, defining the search space as 500bp upstream of the transcription start site (TSS) and 100bp downstream around the TSS of the gene where the motif is scored."<br>Response:<br>Thank the reviewers for their careful review and valuable comments! We will follow your suggestion to add appropriate Spaces between numbers and units to improve the normativity and readability of the text. We did a careful review of the document to ensure that all similar formatting issues had been corrected.
**Additional Information:**	
**Question**	**Response**
Are you submitting this manuscript to a special series or article collection?	No
**Experimental design and statistics**<br><br>Full details of the experimental design and statistical methods used should be given in the Methods section, as detailed in our Minimum Standards Reporting Checklist. Information essential to interpreting the data presented should be made available in the figure legends.<br><br>Have you included all the information requested in your manuscript?	Yes
**Resources**<br><br>A description of all resources used, including antibodies, cell lines, animals and software tools, with enough information to allow them to be uniquely identified, should be included in the Methods section. Authors are strongly encouraged to cite Research Resource Identifiers (RRIDs) for antibodies, model organisms and tools, where possible.<br><br>Have you included the information requested as detailed in our Minimum Standards Reporting Checklist?	Yes
**Availability of data and materials**	Yes

All datasets and code on which the conclusions of the paper rely must be either included in your submission or deposited in publicly available repositories (where available and ethically appropriate), referencing such data using a unique identifier in the references and in the "Availability of Data and Materials" section of your manuscript.<br><br>Have you have met the above requirement as detailed in our Minimum Standards Reporting Checklist?	

Manuscript

Click here to access/download;Manuscript;Manuscript_20250123.docx

Exploring the Cellular and Molecular Basis of Murine Cardiac Development through Spatiotemporal Transcriptome Sequencing

Jingmin Kang[1,2,*], Qingsong Li[1,2,*], Jie Liu[3,*], Lin Du[1,4, ‡], Peng Liu[1], Fuyan Liu[1,2], Yue Wang[1,2,5], Xunan Shen[1,2], Xujiao Luo[1], Ninghe Wang[6], Renhua Wu[6], Lei Song[3,7,8], Jizheng Wang[7,#], Xin Liu[1,2,#]

[1] BGI Research, Beijing, China

[2] BGI Research, Shenzhen, China

[3] Cardiomyopathy Ward, Fuwai Hospital, National Center for Cardiovascular Disease, Chinese Academy of Medical Science and Peking Union Medical College, 167, Beilishilu, Xicheng District, Beijing 100037, China

[4] College of Life Sciences, University of Chinese Academy of Sciences, Beijing 100049, China

[5] State Key Laboratory of Quality Research in Chinese Medicine and Institute of Chinese Medical Sciences, University of Macau, Macao, China

[6] Clin Lab, BGI Genomics, Tianjin, China

[7] State Key Laboratory of Cardiovascular Disease, Fuwai Hospital, National Center for Cardiovascular Diseases, Chinese Academy of Medical Sciences and Peking Union Medical College, 167, Beilishi Road, Xicheng District, Beijing 100037, China

[8] National Clinical Research Center of Cardiovascular Diseases, Fuwai Hospital, National Center for Cardiovascular Diseases, Chinese Academy of Medical Sciences and Peking Union Medical College, 167, Beilishilu, Xicheng District, Beijing 100037, China

* These authors contributed equally.

‡ Senior author

# Correspondence should be addressed to Jizheng Wang (jzwang@hotmail.com) and Xin Liu (liuxin@genomics.cn).

Jingmin Kang [0009-0006-5901-5268]; Lei Song [0000-0001-5958-9321]; Jizheng Wang [0000-0002-2961-524X]; Xin Liu [0000-0003-3256-2940]

Abstract

Spatial transcriptomics is a powerful tool that combines molecular data with spatial information, enabling a deeper understanding of tissue morphology and cellular interactions. In this study, we employed state-of-the-art spatial transcriptome sequencing technology to investigate the development of the mouse heart and establish a comprehensive spatiotemporal cell atlas of early murine cardiac development. Through the analysis of this atlas, we elucidated the spatial organization of cardiac cellular lineages and their interactions during development. Notably, we observed dynamic changes in gene expression within fibroblasts and cardiomyocytes. Furthermore, we identified critical genes including *Igf2*, *H19*, *Tcap*, as well as transcription factors of *Tcf12* and *Plagl1*, that may be associated with the loss of myocardial regeneration ability during early heart development. Moreover, we successfully identified marker genes, such as *Adamts8* and *Bmp10*, that can distinguish between the left and right atria. Our study provides novel insights into murine cardiac development and offers a valuable resource for future investigations in the field of heart research.

Background

Single-cell sequencing has emerged as a crucial tool for investigating the cellular and molecular aspects of heart and cardiac diseases. This approach enables the identification of previously unknown cell types, as well as the characterization of gene expression patterns and regulatory networks in single cell level[1-4]. Through the use of single-cell sequencing, researchers have made significant progress in understanding various cardiac diseases, including heart failure[5,6], arrhythmia[7], and cardiomyopathy[8,9]. These advancements have provided novel insights into disease mechanisms and potential therapeutic targets. However, despite these achivements, there is still a lack of comprehensive understanding regarding the cellular and molecular features of the heart during cardiac development and diseases. This knowledge gap is primarily due to technical limitations inherent in single-cell sequencing, especially the absence of spatial information and biases associated with different cell types[10]. Therefore, it is crucial to explore the spatial organization of heart cells and utilize this spatial information to comprehensively investigate the cellular basis of cardiac development and diseases.

Spatial transcriptomics is a rapidly advancing field that aims to integrate spatial information with transcriptomic data, allowing for the investigation of tissue organization and cellular interactions[11]. Recent advancements in spatial transcriptome technologies, such as *in situ* sequencing[12] and capture-based spatial transcriptome sequencing[13], have revolutionized the simultaneous visualization and quantification of gene expression *in situ*, eliminating the need for tissue dissociation. These innovative approaches have proven successful in studying various biological systems, including the brain[14], developing embryo[13], and disease contexts such as cancer[15] and cardiovascular disease. In the field of cardiac research, spatial transcriptomics has been employed to explore cardiac development in chicken[16], providing novel insights into the interplay between cellular differentiation and morphogenesis that underlie

heart function and pathology. To comprehensively study organs like the heart, the application of spatial transcriptome technologies should be further expanded, which will enable a more comprehensive understanding of the spatial organization of cells within the heart and its implications for cardiac function and disease.

Studies on the mouse heart have demonstrated its regenerative capacity during the neonatal stage, which diminishes as it matures[17]. To gain a deeper understanding of the mechanisms underlying neonatal heart regeneration and explore possible strategies to enhance regeneration, various approaches including genetic manipulation, tissue engineering, and stem cell therapy have been investigated. However, despite these efforts, a comprehensive understanding of these mechanisms is still lacking, highlighting the need for further research. In this study, we employed SpaTial Enhanced REsolution Omics-sequencing (Stereo-seq)[13] to construct a spatiotemporal cell atlas of developing mouse hearts. This atlas encompasses spatial transcriptome data from both regeneration-capable neonatal hearts and relatively mature hearts. By analyzing the dynamics of cellular and gene expression during heart development, we identified specific genes that may be associated with the loss of regeneration ability. Our findings provide new insights into murine cardiac development and serve as a valuable resource for future investigations in the field of heart research.

Results

Constructing a spatiotemporal atlas of mouse heart during early development

To create a comprehensive spatiotemporal transcriptomic atlas of early mouse heart development, we carefully selected four specific time points, including embryonic Day 20 (E20), postnatal Day 1 (P01), postnatal Day 4 (P04), and postnatal Day 14 (P14). Heart samples were obtained by freezing and embedding two mice at each time point. From each heart, three adjacent frozen sections were chosen from the middle

region, and subjected to spatial transcriptome sequencing using the Stereo-seq technique (**Figure 1a**). In total, we obtained six spatiotemporal sections with three technical replicates for each time point, resulting in 22 high-quality sections after excluding any low-quality data. This dataset, consisting of high quality sections, represents a substantial resource for early mouse heart development (**Supplementary Figure 1a**). We then performed cell segmentation to obtain cell bins, based on a bin size of approximately 25 μm (bin 50), resulting in 330,857 cell bins in total (**Table S1**). For these obtained cell bins, we performed gene expression-based cell clustering and utilized known marker genes to annotate the cell types. This analysis revealed the presence of 14 distinct cell types (**Figure 1b, Supplementary Figure 1b and Table S2**). By identifying differentially expressed genes, we further determined marker genes for each cell type (**Figure 1c**). To generate a single-cell transcriptomic atlas with spatial information, we mapped the annotated cell types to their respective spatial positions (**Figure 1d**). This atlas provides a visual representation of the location of each cell and the expression levels of individual genes *in situ*. Overall, we successfully constructed a comprehensive spatiotemporal transcriptomic atlas of mouse heart development.

Leveraging this constructed atlas, we conducted a detailed investigation into the cellular composition of the heart during early development. Our analysis revealed that cardiomyocytes were the predominant cell type, Cardiomyocytes constitute the primary cell type, wherein atrial cardiomyocytes (aCM) account for approximately 21% of the total cell population, and ventricular cardiomyocytes (vCM) represent approximately 57% of the overall cell proportion (**Figure 1e**). In terms of developmental dynamics, we observed an increase in the proportion of vCM over time, accompanied by a decrease in the proportion of aCM (**Figure 1e**). This suggests a major shift in the cellular composition of the heart during early development. Additionally, we identified a distinct population of fibroblasts (FB) located within the ventricle and atrium, characterized by the expression of *COL* family genes, including

Fbln5. Interestingly, the number of these fibroblasts decreased as heart development progressed (**Figure 1e**). Furthermore, our analysis revealed a significant presence of endothelial cells, primarily situated in the middle region of the ventricle. These endothelial cells may play a crucial role in vascular development and maintenance within the heart. Lastly, we identified pericardial cells (Perd) located on the surface of the heart, which exhibited notable expression of *C3*, *Igfbp6*, and *Msln* genes (**Supplementary Figure 4a**). These pericardial cells likely contribute to the structural integrity and protection of the heart. Collectively, our spatiotemporal transcriptomic atlas of heart development provides a comprehensive understanding of the cellular composition within the heart.

Cardiomyocytes mediated cell-cell interaction weakening during heart development

Cell-cell interactions play a vital role in ensuring the proper functioning of biological systems by facilitating coordination and communication between cells[18,19]. These interactions are crucial for various biological processes, including development. In addition to analyzing the cellular composition using our spatiotemporal atlas of heart development, we delved into the investigation of cell-cell interactions (**Table S3**), particularly focusing on how these interactions change throughout development. Initially, we calculated the overall number and strength of cell interactions at different time points (**Figure 2a**). The result reflected that the number of cell-cell interactions did not significantly decrease until P14, while the strength of interactions gradually diminished over time. This suggests that during early development, the factors involved in cell-cell interactions remain intact, but the interactions weaken as development progresses. Further analysis of the pathways of interaction utilized by cells (**Supplementary Figure S2a**) unveiled distinct patterns of activity at different time points. As time passes, the signals related to the construction of the cell skeleton, such as Fibronectin 1 (FN1), Laminin (LAMININ), and Collagen (COLLAGEN)[20],

gradually weaken. Along with that, signals related to Insulin-like Growth Factor (IGF) cell growth[21] also gradually weaken and disappear after 14 days. This suggests that there is a certain correlation between cell growth and the construction of the cell skeleton. As the cell skeleton is completed, the cell morphology gradually becomes fixed, and probably the cells lose their regenerative ability.

In addition to examining the overall cell-cell interactions at the four stages of development, we conducted a detailed investigation into the interactions between different cell types (**Figure 2b** and **Supplementary Figure 3a-d**). Our analysis revealed that the interactions between aCM, vCM, and FB exhibited the highest strength. Further exploration of the input and output interactions of each cell type (**Figure 2c-d**) unveiled that vCM predominantly acted as the major signal-receiving cell type, while aCM served as the primary signal-output cell type, particularly during the relatively early stages (E20, P01and P04). However, at P14, in addition to the overall weakening of interactions, we observed a substantial reduction in the output strength of aCM, which was previously the main output cell type, despite vCM still receiving strong signals. This substantial reduction in interaction from aCM likely contributed significantly to the overall decrease and weakening of cell-cell interactions during cardiac maturation. Thus, we further analyzed the interaction factors present in different cell types at each of the four stages (**Figure 2e**) to elucidate the changes in cell interaction factors within aCM. Our findings indicated that the stage-specific interaction factors neural cell adhesion molecule (NCAM) and cell adhesion molecule (CADM) at E20 were primarily present in neurons (Neur). Similarly, the stage-specific interaction factors Notch receptor (NOTCH), angiopoietin-like protein (ANGPTL), and platelet endothelial cell adhesion molecule 1 (PECAM1) at P01 were mainly observed in FB and pericytes. At P14, particularly in aCM and vCM, which were previously active in interactions, all cell interaction factors, except for COLLAGEN, were scarcely present. These results further support the notion that the decrease in cell-cell interactions during cardiac maturation can be

attributed to the absence of these interaction factors. In summary, leveraging the spatiotemporal atlas of heart development, we conducted an extensive investigation into cell-cell interactions during cardiac development and identified cell-type-specific interaction factors. Notably, we discovered that a key characteristic of mouse cardiac maturation is the decrease in cell-cell interactions among FB, aCM and vCM.

Investigating detailed cell subtypes in mouse heart and their dynamics

The spatiotemporal atlas provides a valuable subcellular-level transcriptomic dataset, offering spatial positional gene expression information of the heart. This resource enables us to delve into finer cell classification and characterization. Therefore, focusing on the three most abundant and prominent cell types, namely vCM, aCM, and FB, we conducted a comprehensive cell type annotation. By analyzing their gene expression characteristics, we successfully annotated vCM, aCM, and FB into 9, 7, and 5 distinct cell subtypes, respectively (**Figure 3a-c**). To gain further insights into these annotated subtypes, we examined their gene expression profiles and identified subtype-specific genes (**Figure 3d-f**). Leveraging these genes that exhibited specific expression patterns within each subtype, we conducted gene function enrichment analysis (**Figure 3g-i**). This analysis allowed us to uncover the functional roles and pathways associated with each cell subtype.

Based on the above cell clustering results, we further mapped these subtype cells to their physical locations in the heart **(Supplementary Figure 4b-d)**. We observed that certain subtypes exhibited obviously distinct spatial distribution characteristics, which showed consistency with their possible functions. For example, cells from one subtype of vCM (vCM4), were found to aggregate near the ventricular cavity (**Supplementary Figure 4e**), and specially expressed genes of these cells were enriched in functions related to muscle organ development, muscle system process,

cardiac muscle contraction, myofibril assembly, etc.. (**Figure 3g, Table S4)** Both their spatial distribution and functional enrichment suggest vCM4 cells to be involved in the contraction and relaxation activities of the heart. Meanwhile, cells from the other subtype of aCM (aCM5), were observed to aggregate in the region of the tricuspid and bicuspid valves (**Supplementary Figure 4f**). Enrichment of their specially expressed genes indicated their possible functions related to oxidative phosphorylation, energy derivation by oxidation of organic compounds, generation of precursor metabolites and energy, cellular respiration, respiratory electron transport chain, etc.. (**Figure 3h, Table S5**) We thus deduce that these cells may play a crucial role in providing energy for the opening and closing movements of the tricuspid and bicuspid valves, contributing to the regularity and effectiveness of cardiac pulsation.

Similarly for fibroblasts, we found cells from one subtype (FB4) to aggregate in the region of the aorta (**Supplementary Figure 4g**), with a higher proportion at E20, P01, and P04, and lower proportion at P14. We found the specially expressed genes of these cells to be enriched in functions related to cell-substrate adhesion and cardiac ventricle morphogenesis (**Figure 3i, Table S6**). This suggests FB4 cells to be associated with the construction of the cardiac cell scaffold, and this activity is more active in the early stages of heart development. While not all subtypes could be functionally annotated, this investigation allows us to gain a deeper understanding of the dynamic changes occurring within these cell subtypes throughout the developmental stages of the heart.

Cell trajectories revealed critical genes involved in regeneration ability loss

While human cardiac muscle cells lack regenerative capacity, mouse cardiac muscle cells possess the ability to regenerate during early development[22]. It is cruicial to understand the mechanisms underlying the loss of regenerative ability in mouse cardiac muscle cells during the developmental process. This understanding will unravel the molecular intricacies of cardiac muscle regeneration and aid future

research on important heart diseases. Our heart spatiotemporal atlas of the heart encompasses four time points during early embryonic development. The first three time points correspond to the regenerative period of the mouse heart, while the final time point (P14) signifies the loss of regenerative ability[23]. To investigate the cellular and molecular changes associated with cardiac muscle regeneration, we conducted an in-depth analysis by comparing the data from the final time point with the preceding three time points. Pseudotime analysis using Monocle2 was conducted on the subtypes of vCM, aCM, and FB. Given that cardiomyocytes are deemed terminally differentiated cells, FB was designated as the origin in the pseudotime trajectory (**Figure 4a** and **Supplementary Figure 5a-b**). This analysis revealed distinct temporal trends for these major cell types. At E20, aCM and FB exhibited high similarity compared to vCM. As time progressed, all three major cell types underwent further differentiation and by P14, the states of several cell types become consistent. The branching pattern observed among these subtypes (**Figure 4a**) indicated clear differentiation during heart development. Moreover, genes can be clustered into three cell state based on their expression changes along the trajectory (**Figure 4b, Table S7**). During the development of the heart and maturation of cardiac muscle cells, we observed notable changes in gene expression levels that correlated with the loss of regenerative capacity and the acquisition of contractile ability and cardiac function. Specifically, the expression levels of *Igf2* (Insulin Like Growth Factor 2 gene)[24,25], *H19* (a long noncoding RNA gene, H19 Imprinted Maternally Expressed Transcript)[26,27], and other genes associated with cell differentiation gradually decreased. Conversely, the expression levels of *Tcap* (Titin-Cap gene)[28], *Myh6* (Myosin Heavy Chain 6 gene)[29], *Atp2a2* (ATPase Sarcoplasmic, Endoplasmic Reticulum Ca^{2+} Transporting 2 gene)[30], and other genes gradually increased, indicating the acquisition of contractile ability and the initiation of cardiac function. These changes in gene expression profiles provide insights into the molecular processes underlying the transition from regenerative capacity to contractile ability

during cardiac muscle development.

Transcription factors (TFs) are pivotal in regulating gene expression during various biological processes[31]. Thus we conducted a detailed investigation into the changes in transcription factors during cardiac development to identify TFs that may contribute to the loss of myocardial cell regeneration capacity (**Table S8**). Base on the regulon specificity score (RSS, **Figure 4c**), we categorized the identified TFs into two groups. The first group comprised TFs that exhibited higher activity levels in the early stages of cardiac development but gradually decreased in specificity as development progressed. In contrast, the second group consisted of TFs that displayed higher activity levels during the later stages of cardiac maturation. Among TFs of the second group, we identified several important TFs that have been previously reported to be associated with cardiac development. These include *Wt1* (Wilms' tumor 1 gene), *Prrxl1* (Paired related homeobox 1 gene), and *Srebf1* (sterol regulatory element binding transcription factor 1). For instance, *Wt1* contains four zinc finger motifs at the C-terminus, which are crucial for DNA binding and gene activation[32]. In the context of cardiac development, *Wt1* primarily regulates processes such as epithelial-to-mesenchymal transition and angiogenesis, and it has been shown to play a critical role in early cardiac development[33]. Additionally, our analysis uncovered previously unreported TFs that may play a role in cardiac maturation, such as *Mlx* (Max-like protein X gene), which warrants further investigation. On the other hand, the TFs in the first group are more likely to be associated with the loss of myocardial cell regeneration capacity during this process. Among these, we identified *Tcf12* (transcription factor 12)[34], *Plagl1* (Pleomorphic adenoma gene-like 1), and other transcription factors that exhibited decreased activity levels with cardiac maturation.

In addition, as our analysis of differential gene expression identified *Igf2* to be possibly involved in heart development and regeneration ability loss, we further investigated whether TFs targeting the *Igf* genes exhibited expression changes

associated with cardiac maturation. Speciafically, in our analysis of fibroblasts and myocardial cells, we identified six transcription factors targeting *Igf2*, including the aforementioned *Plagl1*. Notably, we observed sustained decrease in the transcription factor activity of *Plagl1* along fibroblasts and myocardial cells during cardiac development and maturation (**Figure 4d**). Furthermore, when examing its TF activity correlation with the expression of other genes (**Figure 4e**) we found that the TF *Plagl1* exhibited the strongest correlation with *Igf2*. We further validated the temporal expression changes of Igf2 and Plagl1 through Quantitative Real-time polymerase chain reaction (qPCR) (**Figure 4f-g**). We found that both genes exhibited a significant decrease in expression on postnatal day 14, which is consistent with our spatial data.

Besides, We investigated the characteristics of early mouse cardiac cells using spatial transcriptomics data from early mouse embryos[35]. Firstly, we performed clustering on the spatial transcriptomics data of early embryos (E9.5, E10.5, E11.5 and E12.5) and identified cluster 8 and 16 as undifferentiated cardiac cells using marker genes specific to myocardial cells (**Supplementary Figure 6a-c**). Furthermore, we identified the expression of key genes and transcription factors in early cardiac cells. *Igf2*, *H19*, *Tcf12*, and *Plagl1* were highly expressed, while *Wt1*, *Prrxl1*, and *Srebf1* were expressed at lower levels, consistent with the developmental changes observed in cardiac cells (**Supplementary Figure 6d**).

This finding suggests that *Plagl1* may have been regulating *Igf2* during early heart development. These findings point to *Plagl1* as a potential TF involved in the differentiation and regeneration of myocardial cells. However, further comprehensive research is necessary to delve deeper into these findings and investigate the functional roles of the identified candidate genes.

Identification of atrial asymmetry related genes

By utilizing the spatiotemporal transcriptomic atlas, we can leverage the spatial information of gene expression for molecular-level investigations. The left-right

asymmetry of the heart is crucial for its functional performance[36], making it essential to understand the development of the left and right ventricles and atria. To achieve this, we first divided the left and right atrial regions based on their positions for all the Stereo-seq sections. We then validated these divisions by performing HE staining on adjacent sections (**Figure 5a-d**). This approach ensured the accuracy of our spatially defined regions. In contrast, when clustering and analyzing without incorporating spatial positional information, using only the Uniform Manifold Approximation and Projection (UMAP) plot of all cells from each chip, there would be a certain degree of error. We mapped the results of unsupervised clustering of the left and right atria performed solely based on the UMAP plots onto our spatial atlas (Figures 5a-d, Supplementary Figures 7a-d), and counted the number of cells predominantly distributed in the left or right atrium, calculating the percentage of these cells compared to the total cells in the left or right atrium. We found that the proportion of left atrial cells that could be directly differentiated to be lower (69%, 70%, 97%, and 88%) compared to the right atrial cells (93%, 94%, 90%, and 92%) at the four time points (**Figure 5e**, **Table S9**). These findings highlight the importance of considering spatial information for accurate identification and analysis of left and right atrial cells.

Subsequently, following the successful distinction between left and right atrial cells, we proceeded to investigate the disparities in gene expression between these two regions. By comparing the cells of the left and right atria at four time points, we identified a set of genes that were differentially expressed (**Figure 5f**). This set comprised genes that were upregulated in the left atrium (**Figure 5g**) and genes that were upregulated in the right atrium (**Figure 5h**) at the four designated time points. Specifically, in the left atrium, we observed 17, 20, 27, and 26 upregulated genes at E20, P01, P04, and P14, respectively. Conversely, the right atrium exhibited 23, 63, 67, and 22 upregulated genes at the corresponding time points. To gain further insights into the functional implications of these gene expression differences, we conducted a functional enrichment analysis of the identified genes **(Supplementary**

Figure 7e). Notably, we found that the upregulated genes in the right atrium were significantly enriched in gene ontology (GO) terms related to heart development. In contrast, the upregulated genes in the left atrium did not exhibit substantial enrichment in any heart development-related GO terms.

We then conducted an investigation into specific genes that may be associated with atrial asymmetry. Within one of the previously mentioned enriched GO terms (GO:0003228, atrial cardiac muscle tissue development), we identified four genes, including *Pitx2*, *Bmp10*, *Eng* and *Adamts8*. *Pitx2*, a transcription factor known for its involvement in regulating left-right asymmetry development of the heart and other organs, has been established as a gene specific to the left atrium[37]. Our analysis revealed significant upregulation of *Pitx2* in the left atrium at the P01 and P04 time point (**Supplementary Figure 8a**). *Bmp10*, known as a regulatory gene of *Pitx2*, exhibited specific expression in the right atrium throughout all stages (**Supplementary Figure 8b**). Furthermore, *Eng* displayed significantly higher expression in the right atrium at the P04 stage(**Supplementary Figure 8c**). In addition, we consistently observed high expression of *Adamts8* in the left atrium throughout the entire developmental stage(**Supplementary Figure 8d**). These findings shed light on the potential involvement of these genes in atrial asymmetry and provide valuable insights into the molecular mechanisms underlying the development of the left and right atria. Further research is warranted to explore the functional roles of these genes and their contributions to atrial cardiac muscle tissue development.

Discussion

The heart plays a crucial role in our physiology, and conducting single-cell and spatial transcriptomic studies on this organ can significantly enhance our understanding of cellular and molecular changes occurring during heart development and related diseases. Such studies provide essential insights into the molecular mechanisms underlying heart-related conditions and lay the groundwork for the identification of novel targets for the treatment of heart disease[1,6,10,38,39]. In our study, we constructed a comprehensive spatiotemporal transcriptomic atlas of early mouse heart development. We examined the changes occurring at the cellular and gene levels throughout the developmental process, including alterations in cell type composition, intercellular communication, shifts in cardiac cell types during development, and the identification of candidate genes crucial for cardiac development. Our findings largely align with previous single-cell studies conducted in mice, validating the reliability of our spatiotemporal atlas. For instance, we identified the transcription factor *Wt1* as potentially playing crucial role in mouse heart development, which has already been reported in a previous mouse single-cell study[39]. However, our spatiotemporal atlas allowed us to systematically describe the cellular and gene-level changes that occur during early heart development, providing a more comprehensive understanding of the process. Moreover, we identified novel genes that may be associated with heart development. Utilizing the spatial information available in our atlas, we compared the cell types present in the left and right atria and identified genes with specific expression patterns throughout development. This analysis deepened our understanding of heart asymmetry and shed light on the molecular underpinnings of this phenomenon. Notably, our study focused on the unique aspects of mouse heart development in the early stages, distinguishing it from previously published spatiotemporal atlases of chicken heart development[16]. We specifically explored cell type changes and potential genes related to regeneration, which are distinctive to the early developmental stage of the mouse heart.

Cardiac regeneration in mice has been a prominent focus of heart research, with previous studies identifying molecules and genes associated with early cardiac regeneration in mice[40-43]. However, the mechanisms underlying myocardial cell regeneration specific to early mouse hearts, which are absent in later stages and other mammals, remain poorly understood. In our study, we conducted a comparative analysis of spatiotemporal transcriptomic data from early and late stages of mouse hearts, leading us to identify *Igf2* and the transcription factor *Plagl1*, which regulates *Igf2*, as potential contributors to early mouse cardiac regeneration. Previous studies have suggested the involvement of *Igf2* in the regeneration of the heart and other tissues in mice[44-46], while other studies, including a recent preprint article, have described the role of *Plagl1* in retinal regeneration in mice[47,48]. Notably, *Plagl1* has been shown to have inhibitory effects on cell proliferation and act as a tumor suppressor in humans[49,50]. Additionaly, *Plagl1* is an imprinted gene, exhibiting specific expression of the paternal allele in multiple tissues and being implicated in the pathogenesis of congenital heart disease[51]. Given that *Plagl1* regulates numerous downstream genes, the regulatory pathway from *Plagl1* to *Igf2* and its impact on early myocardial cell regeneration remain unclear. Our study highlights the significance of investigating the *Plagl1*-*Igf2* regulation pathway for further research on myocardial cell regeneration in mice and even humans, laying the foundation for subsequent mechanistic studies.

Our study constructed a spatiotemporal transcriptomic atlas of early mouse heart development and proposed cellular and molecular changes related to mouse heart development based on the analysis of the atlas. However, establishing an atlas that covers more time points, conducting further research on heart regeneration and heart asymmetry-related studies, and performing functional validation and mechanistic analysis are potential future directions based on our study.

Methods

Experimental animal mice and heart sample preparation

The Institutional Animal Care and Use Committee of BGI thoroughly reviewed and granted approval for the animal experimental protocol. All procedures pertaining to mouse experiments in this study strictly adhered to the ethical regulations and guidelines outlined in the Animal Experimentation protocols of BGI, in addition to compliance with the Guidelines for the Care and Use of Laboratory Animals in China (License Number BGI-IRB A21030-T1). All mice were housed in standard SPF conditions with temperatures of 65–75 °F (~18–23 °C) and with 40–60% humidity. In this study, male C57BL/6 mice at embryonic day 20 (E20), postnatal day 1 (P1), postnatal day 4 (P4), and postnatal day 14 (P14) were used; 2 mice were used for each experiment.

Anesthetize mice with chloral hydrate, then extract the hearts and place them in pre-cooled 1x PBS. Thoroughly clean the heart surfaces, ensuring the removal of blood. Employ gauze to absorb any excess 1x PBS from the heart surfaces. Subsequently, position the hearts in a dish on ice, awaiting subsequent processing steps, such as embedding.

Stereo-seq sample and library preparation

Dissect the hearts of mice at four time points from the thoracic cavity, remove the pericardium using fine forceps, rinse the surface and intracardiac blood in cold PBS, blot dry with gauze, and finally embed the hearts in Tissue-Tek O.C.T. Compound (Sakura, 4583) and rapidly freeze them on dry ice.

The stereo-seq libraries were prepared using Stereo-seq[13]. Briefly, Longitudinally section the OCT-embedded heart into 10 mm thick slices using a Leica CM1950 cryostat. These slices were then adhered to the Stereo-seq chip. The chip was completely immersed in methanol at -20 degrees Celsius for 30 minutes. Afterward,

t

h

e

permeabilization (6 minutes for E20 and P01, 7 minutes for P04, 9 minutes for P14). Probes on the chip, equipped with coordinate tags, captured the polyA RNA released in situ from the tissue sections. Following this step, the captured polyA RNA underwent in situ reverse transcription, resulting in the synthesis of cDNA with coordinate tags. Subsequently, after tissue digestion to eliminate any residual sliced tissue from the chip, cDNA with coordinate tags was liberated using release enzymes and recovered utilizing magnetic beads. Following cDNA amplification, 20ng of cDNA were employed for fragmentation and additional amplification to finalize library construction. Ultimately, sequencing was executed using the MGI DNBSEQ sequencing platform.

Hematoxylin and Eosin Staining (HE)

The HE staining was performed following standard protocols[52]. 10 μm OCT frozen sections were mounted on glass slides and fixed in 4% paraformaldehyde in 1x PBS at room temperature for 10 minutes. Subsequently, the sections were incubated in Hematoxylin for 7 minutes, washed in Nuclease-free water, incubated in Eosin for 2 minutes, and washed again in Nuclease-free water. After air-drying at room temperature, bright-field images were captured using a Motic fluorescence microscope.

Binning data of spatial stereo-seq data

The Stereo-seq transcriptomic data in this study underwent processing using the Stereo-seq Analysis Workflow (SAW) software suite (RRID:SCR_025001)[53]. This suite facilitated the mapping of sequencing reads onto tissue sections, allowing for the quantitative assessment of gene expression levels at each spatial position (Spot). Typically, the “bin_size” parameter is configured to group nanochannels within a specific range into a bin unit. Through a statistical analysis of cell and bin sizes across all tissues, we selected an individual bin size of 50 (~25 μm) as the fundamental unit

for downstream analysis of the transcriptomic data generated by Stereo-seq in mouse hearts[13]. This ensured that each unit contained a sufficient number of genes to represent its molecular characteristics. Moreover, in the data quality control phase, we excluded bin 50 units with low gene counts. By examining the gene count distribution curve for each bin 50, we observed that low-quality bin 50 units tended to cluster into a small peak, and we determined the filtering threshold as the minimum value on the right side of this peak.

Cell clustering and annotation

The downstream analysis of the mouse heart transcriptomic data primarily involved the Seurat software package (version 4.1.1)[54,55]. Initially, we utilized the "SCTransform" function for each spatial section data normalization, scaling, and integration across distinct datasets from various time points. Then e integrated the Seurat objects of each chip for downstream analysis. Subsequently, PCA reduction and UMAP embedding were applied for dimensionality reduction. The "FindNeighbors" function (utilizing the top 30 principal components) performed cell clustering, followed by the "FindClusters" function for graph-based clustering. Marker genes for different clusters were identified using the "FindAllMarkers" function in Seurat with parameters set to (min.pct=0.1, logfc.threshold=0.25), and a filter based on pvalue.adj < 0.05 was applied. Cell clusters were determined using established cell type-specific markers for each cluster. For a more nuanced categorization of cell types, bins associated with a particular cell type, or groups comprising related cell types, underwent additional clustering and annotation. The subcluster annotation process for a specific cell type mirrors the description provided earlier for general cell types.

Cell chat (Ligand-receptor analysis)

We utilized the CellChat package (version 1.5.0)[56] for the inference, analysis, and visualization of the cell-cell communication network in the mouse heart transcriptomic dataset. Initially, we created a new CellChat object for each time point using the CellChatDB.mouse database within the Seurat framework. This database comprises 2021 validated molecular interactions, encompassing 60% secreted autocrine/paracrine signaling interactions, 21% extracellular matrix-receptor interactions, and 19% cell-cell contact interactions. After preprocessing the expression data by identifying over-expressed genes and interactions, we employed the 'computeCommunProb' and 'filterCommunication' functions to infer the cellular communication network and compute communication probabilities. The 'computeCommunProbPathway' function calculated communication probabilities at the signaling pathway level by summarizing probabilities associated with ligands-receptors interactions in each pathway. For an aggregated view of the cell-cell communication network, the 'aggregateNet' function was used to count links or summarize communication probabilities for each communication pair. Subsequently, the three CellChat objects for each time point were merged using the 'mergeCellChat' function for downstream analysis.

Cell differentiation inference

To facilitate subsequent trajectory analysis and transcription factor analysis, we randomly selected 500 cells from each subtype of the three cell types (FB, aCM, vCM) using the 'subset' function with the 'downsample' parameter of the SeuratObject [Satija R] package. To analyze the differentiation trajectory of mouse heart cells and investigate their pseudotime relationships, we employed the R package Monocle 2 (version 2.22.0)[57]. Initially, we used the "importCDS" function (object, import_all = F) to convert the raw counts into the CellDataSet format. Subsequently, the "estimateSizeFactors" and "estimateDispersions" functions were employed to precompute crucial parameters related to the data. The "differenceGeneTest" function

was used to select potential ordering genes (qval < 0.01), providing information for ordering cells along the pseudotime trajectory. Dimensionality reduction and clustering analysis were performed using the "reduceDimension" function (reduction_method = "DDRTree"), with "fullModelFormulaStr" set for cell type and time point. Trajectory inference was executed using the default parameters through the "orderCells" function.

Differential gene expression (DEG) analysis and GO enrichment

Differential expression analysis of the same cell type between two time points was conducted using the 'FindMarkers' function in Seurat packages. We filtered significant differentially expressed genes (DEGs) with an adjusted p-value < 0.05 and an absolute average log2 fold change > 0.5. Specifically for the identification of the left and right atria, differential analysis was performed using data from the corresponding spatial sections. The 'FindMarkers' function in the Seurat package was employed, focusing on highly variable genes based on criteria such as p-value < 0.05 and logFC > 0.5. For a deeper understanding of the functional implications of these genes, we conducted Gene Ontology (GO) enrichment analysis in the Biological Process category. This was achieved using the clusterProfiler software package (version 4.7.1)[58], with gene annotations sourced from the org.Mm.eg.db[59] annotation database for mouse loci.

Transcription factor regulation activity prediction

We utilized pySCENIC[60] for transcription factor analysis across three cell types (FB, aCM, and vCM). Initially, we downloaded the relevant cisTarget database for mice with version mm10[61]. This database was constructed using the 2022 SCENIC+ motif collection, defining the search space as 500bp upstream of the transcription start site (TSS) and 100bp downstream around the TSS of the gene where the motif is scored.

We inferred potential transcription factors (TFs) and computed the transcription factor

activity for each cell using the default workflow of pySCENIC[62] (RRID:SCR_025802). Subsequently, the SCENIC package (v1.3.1) [60] in R was employed to calculate the regulon specificity score of the transcription factors at different time points. The transcription factor activity was integrated with the gene expression matrix, and the correlation coefficient between the transcription factors and target genes was determined using the cor.test function from the stats (v4.3.0)[63] package.

Acknowledgements

We sincerely thank the support provided by the China National GenBank, BGI Research (CNGB). This work was supported by the CAMS Innovation Fund for Medical Sciences (2023-I2M-1-001), the National Natural Science Foundation of China (82170371 and 82300396), and the National High Level Hospital Clinical Research Funding (2023-GSP-QN-3).

Author contributions

X.L. and J.W. conceived and leaded the study. J.K., J.L., X.L., N.W. and R.W. carried out the experiments. Q.L., L.D., P.L., X.S. and F.L. analyzed the data. X.L. and J.K. wrote the manuscript. Q.L., L.D., J.L., L.S. and F.L. revised the manuscript. All authors read and approved the final manuscript.

Competing interests

The authors declare no conflict of interests.

Data availability

All raw data generated by this study can be found in NCBI-SRA with the accession number PRJNA1148773. Processed data have been deposited in the China National GeneBank Database (CNGBdb) in the China National GeneBank Sequence Archive

(CNSA) with the accession number STT0000062. All spatial transcriptomic data of mouse embryo samples (E9.5, E10.5, E11.5 and E12.5) are available at NCBI Gene Expression Omnibus with accession number GSE178636. All supporting data and materials are available in the GigaScience repository, GigaDB [64].

References

1 Skelly, D. A. *et al.* Single-Cell Transcriptional Profiling Reveals Cellular Diversity and Intercommunication in the Mouse Heart. *Cell Rep* **22**, 600-610, doi:10.1016/j.celrep.2017.12.072 (2018).

2 DeLaughter, D. M. *et al.* Single-Cell Resolution of Temporal Gene Expression during Heart Development. *Dev Cell* **39**, 480-490, doi:10.1016/j.devcel.2016.10.001 (2016).

3 Cui, Y. *et al.* Single-Cell Transcriptome Analysis Maps the Developmental Track of the Human Heart. *Cell Rep* **26**, 1934-1950 e1935, doi:10.1016/j.celrep.2019.01.079 (2019).

4 Tabula Muris, C. *et al.* Single-cell transcriptomics of 20 mouse organs creates a Tabula Muris. *Nature* **562**, 367-372, doi:10.1038/s41586-018-0590-4 (2018).

5 Martini, E. *et al.* Single-Cell Sequencing of Mouse Heart Immune Infiltrate in Pressure Overload-Driven Heart Failure Reveals Extent of Immune Activation. *Circulation* **140**, 2089-2107, doi:10.1161/CIRCULATIONAHA.119.041694 (2019).

6 Koenig, A. L. *et al.* Single-cell transcriptomics reveals cell-type-specific diversification in human heart failure. *Nat Cardiovasc Res* **1**, 263-280, doi:10.1038/s44161-022-00028-6 (2022).

7 Yuan, P. *et al.* Single-Cell RNA Sequencing Uncovers Paracrine Functions of the Epicardial-Derived Cells in Arrhythmogenic Cardiomyopathy. *Circulation* **143**, 2169-2187, doi:10.1161/CIRCULATIONAHA.120.052928 (2021).

8 Wang, L. *et al.* Single-cell reconstruction of the adult human heart during heart failure and recovery reveals the cellular landscape underlying cardiac function. *Nat Cell Biol* **22**, 108-119, doi:10.1038/s41556-019-0446-7 (2020).

9 Chaffin, M. *et al.* Single-nucleus profiling of human dilated and hypertrophic cardiomyopathy. *Nature* **608**, 174-180, doi:10.1038/s41586-022-04817-8 (2022).

10 Yamada, S. & Nomura, S. Review of Single-Cell RNA Sequencing in the Heart. *Int J Mol Sci* **21**, doi:10.3390/ijms21218345 (2020).

11 Vickovic, S. *et al.* High-definition spatial transcriptomics for in situ tissue profiling. *Nat Methods* **16**, 987-990, doi:10.1038/s41592-019-0548-y (2019).

12 Ke, R. *et al.* In situ sequencing for RNA analysis in preserved tissue and cells. *Nat Methods* **10**, 857-860, doi:10.1038/nmeth.2563 (2013).

13 Chen, A. *et al.* Spatiotemporal transcriptomic atlas of mouse organogenesis using DNA nanoball-patterned arrays. *Cell* **185**, 1777-1792 e1721, doi:10.1016/j.cell.2022.04.003 (2022).

14 Wei, X. *et al.* Single-cell Stereo-seq reveals induced progenitor cells involved in axolotl

brain regeneration. *Science* **377**, eabp9444, doi:10.1126/science.abp9444 (2022).

15 Wu, L. *et al.* An invasive zone in human liver cancer identified by Stereo-seq promotes hepatocyte-tumor cell crosstalk, local immunosuppression and tumor progression. *Cell Res*, doi:10.1038/s41422-023-00831-1 (2023).

16 Mantri, M. *et al.* Spatiotemporal single-cell RNA sequencing of developing chicken hearts identifies interplay between cellular differentiation and morphogenesis. *Nat Commun* **12**, 1771, doi:10.1038/s41467-021-21892-z (2021).

17 Lam, N. T. & Sadek, H. A. Neonatal Heart Regeneration: Comprehensive Literature Review. *Circulation* **138**, 412-423, doi:10.1161/CIRCULATIONAHA.118.033648 (2018).

18 Sheikh, F., Ross, R. S. & Chen, J. Cell-cell connection to cardiac disease. *Trends in cardiovascular medicine* **19**, 182-190 (2009).

19 Belardi, B., Son, S., Felce, J. H., Dustin, M. L. & Fletcher, D. A. Cell–cell interfaces as specialized compartments directing cell function. *Nature Reviews Molecular Cell Biology* **21**, 750-764 (2020).

20 Lukjanenko, L. *et al.* Loss of fibronectin from the aged stem cell niche affects the regenerative capacity of skeletal muscle in mice. *Nature medicine* **22**, 897-905 (2016).

21 Li, P. *et al.* IGF signaling directs ventricular cardiomyocyte proliferation during embryonic heart development. *Development* **138**, 1795-1805 (2011).

22 Fine, B. & Vunjak-Novakovic, G. Heart regeneration in mouse and human: A bioengineering perspective. *Curr Opin Physiol* **14**, 56-63, doi:10.1016/j.cophys.2020.01.004 (2020).

23 Misra, A. *et al.* Characterizing neonatal heart maturation, regeneration, and scar resolution using spatial transcriptomics. *Journal of Cardiovascular Development and Disease* **9**, 1 (2021).

24 Minuto, F., Palermo, C., Arvigo, M. & Barreca, A. M. The IGF system and bone. *J Endocrinol Invest* **28**, 8-10 (2005).

25 Kang, H. *et al.* Insulin-like growth factor 2 promotes osteogenic cell differentiation in the parthenogenetic murine embryonic stem cells. *Tissue Eng Part A* **18**, 331-341, doi:10.1089/ten.TEA.2011.0074 (2012).

26 Milligan, L. *et al.* H19 gene expression is up-regulated exclusively by stabilization of the RNA during muscle cell differentiation. *Oncogene* **19**, 5810-5816, doi:10.1038/sj.onc.1203965 (2000).

27 Poirier, F. *et al.* The murine H19 gene is activated during embryonic stem cell differentiation in vitro and at the time of implantation in the developing embryo. *Development* **113**, 1105-1114, doi:10.1242/dev.113.4.1105 (1991).

28 Hayashi, T. *et al.* Tcap gene mutations in hypertrophic cardiomyopathy and dilated cardiomyopathy. *J Am Coll Cardiol* **44**, 2192-2201, doi:10.1016/j.jacc.2004.08.058 (2004).

29 van Gorp, P. R. *et al.* Sbk2, a Newly Discovered Atrium-Enriched Regulator of Sarcomere Integrity. *Circulation Research* **131**, 24-41 (2022).

30 Skogestad, J. *et al.* Disruption of Phosphodiesterase 3A Binding to SERCA2 Increases SERCA2 Activity and Reduces Mortality in Mice With Chronic Heart Failure. *Circulation* **147**, 1221-1236, doi:10.1161/circulationaha.121.054168 (2023).

31 Weidemüller, P., Kholmatov, M., Petsalaki, E. & Zaugg, J. B. Transcription factors: Bridge between cell signaling and gene regulation. *Proteomics* **21**, 2000034 (2021).

32 Duim, S. N., Goumans, M. J. & Kruithof, B. P. T. in *Wilms Tumor* (ed M. M. van den Heuvel-Eibrink) (2016).

33 Moore, A. W., McInnes, L., Kreidberg, J., Hastie, N. D. & Schedl, A. YAC complementation shows a requirement for Wt1 in the development of epicardium, adrenal gland and throughout nephrogenesis. *Development* **126**, 1845-1857, doi:10.1242/dev.126.9.1845 (1999).

34 Wang, S. *et al.* Tcf12 is required to sustain myogenic genes synergism with MyoD by remodelling the chromatin landscape. *Communications Biology* **5**, 1201, doi:10.1038/s42003-022-04176-0 (2022).

35 Chen, Y., Zhou, S., Li, M., Zhao, F. & Qi, J. STEEL enables high-resolution delineation of spatiotemporal transcriptomic data. *Briefings in Bioinformatics* **24**, doi:10.1093/bib/bbad068 (2023).

36 Desgrange, A., Le Garrec, J.-F. & Meilhac, S. M. Left-right asymmetry in heart development and disease: forming the right loop. *Development* **145**, dev162776 (2018).

37 Logan, M., Pagán-Westphal, S. M., Smith, D. M., Paganessi, L. & Tabin, C. J. The transcription factor Pitx2 mediates situs-specific morphogenesis in response to left-right asymmetric signals. *Cell* **94**, 307-317 (1998).

38 Li, W. *et al.* Single-cell RNA-seq of heart reveals intercellular communication drivers of myocardial fibrosis in diabetic cardiomyopathy. *Elife* **12**, doi:10.7554/eLife.80479 (2023).

39 Feng, W. *et al.* Single-cell transcriptomic analysis identifies murine heart molecular features at embryonic and neonatal stages. *Nat Commun* **13**, 7960, doi:10.1038/s41467-022-35691-7 (2022).

40 Tan, Y., Duan, X., Wang, B., Liu, X. & Zhan, Z. Murine neonatal cardiac B cells promote cardiomyocyte proliferation and heart regeneration. *NPJ Regen Med* **8**, 7, doi:10.1038/s41536-023-00282-7 (2023).

41 Nakada, Y. *et al.* Hypoxia induces heart regeneration in adult mice. *Nature* **541**, 222-227, doi:10.1038/nature20173 (2017).

42 Han, C. *et al.* Acute inflammation stimulates a regenerative response in the neonatal mouse heart. *Cell Res* **25**, 1137-1151, doi:10.1038/cr.2015.110 (2015).

43 Bassat, E. *et al.* The extracellular matrix protein agrin promotes heart regeneration in mice. *Nature* **547**, 179-184, doi:10.1038/nature22978 (2017).

44 Shen, H. *et al.* Mononuclear diploid cardiomyocytes support neonatal mouse heart regeneration in response to paracrine IGF2 signaling. *Elife* **9**, doi:10.7554/eLife.53071 (2020).

45 Bella, P. *et al.* Blockade of IGF2R improves muscle regeneration and ameliorates Duchenne muscular dystrophy. *EMBO Mol Med* **12**, e11019, doi:10.15252/emmm.201911019 (2020).

46 Near, S. L., Whalen, L. R., Miller, J. A. & Ishii, D. N. Insulin-like growth factor II stimulates motor nerve regeneration. *Proc Natl Acad Sci U S A* **89**, 11716-11720, doi:10.1073/pnas.89.24.11716 (1992).

47 Garcia-Garcia, D., Locker, M. & Perron, M. Update on Muller glia regenerative potential for retinal repair. *Curr Opin Genet Dev* **64**, 52-59, doi:10.1016/j.gde.2020.05.025 (2020).

48 Touahri, Y. *et al.* Plagl1 is part of the mammalian retinal injury response and a critical regulator of Müller glial cell quiescence *bioRxiv*, 2021. doi:10.1101/2021.04.12.439550

49 Liang, X. *et al.* PLAGL1 is associated with prognosis and cell proliferation in pancreatic adenocarcinoma. *BMC Gastroenterol* **23**, 2, doi:10.1186/s12876-022-02609-y (2023).

50 Sievers, P. *et al.* Recurrent fusions in PLAGL1 define a distinct subset of pediatric-type supratentorial neuroepithelial tumors. *Acta Neuropathol* **142**, 827-839, doi:10.1007/s00401-021-02356-6 (2021).

51 Zhao, X. *et al.* Imprinting aberrations of SNRPN, ZAC1 and INPP5F genes involved in the pathogenesis of congenital heart disease with extracardiac malformations. *J Cell Mol Med* **24**, 9898-9907, doi:10.1111/jcmm.15584 (2020).

52 Asp, M. *et al.* A spatiotemporal organ-wide gene expression and cell atlas of the developing human heart. *Cell* **179**, 1647-1660. e1619 (2019).

53 Gong, C. *et al.* SAW: an efficient and accurate data analysis workflow for Stereo-seq spatial transcriptomics. *GigaByte* **2024**, gigabyte111, doi:10.46471/gigabyte.111 (2024).

54 Hao, Y. *et al.* Integrated analysis of multimodal single-cell data. *Cell* **184**, 3573-3587. e3529 (2021).

55 Stuart, T. *et al.* Comprehensive integration of single-cell data. *Cell* **177**, 1888-1902. e1821 (2019).

56 Jin, S. *et al.* CellChat: Inference and analysis of cell-cell communication from single-cell transcriptomics data. *R package version* **1** (2022). DOI: 10.1038/s41596-024-01045-4.

57 Qiu, X. *et al.* Single-cell mRNA quantification and differential analysis with Census. *Nature methods* **14**, 309-315 (2017).

58 Wu, T. *et al.* clusterProfiler 4.0: A universal enrichment tool for interpreting omics data. *The innovation* **2** (2021).

59 Carlson, M., Falcon, S., Pages, H. & Li, N. org. Mm. eg. db: Genome wide annotation for Mouse. *R package version* **3**, 10.18129 (2019). DOI: 10.18129/B9.bioc.org.Mm.eg.db.

60 Aibar, S. *et al.* SCENIC: single-cell regulatory network inference and clustering. *Nature methods* **14**, 1083-1086 (2017).

61 Lab, S. A. cisTarget databases. https://resources.aertslab.org/cistarget/databases/mus_musculus/mm10. (2022).

62 aertslab. pySCENIC. GitHub. https://github.com/aertslab/pySCENIC. (2021).

63 R Core Team, R. R: A language and environment for statistical computing. (2013).

64. Kang J, Li Q, Liu J, et al. Supporting data for "Exploring the Cellular and Molecular Basis of Murine Cardiac Development through Spatiotemporal Transcriptome Sequencing" GigaScience Database. 2025. https://doi.org/10.5524/102645

Figure legend

Figure 1 Spatial and temporal atlas of mouse heart development.

a. Mouse heart sampling and stereo-seq protocol. **b.** Uniform manifold approximation and projection (UMAP) of stereo-seq clustering and annotation of 22 spatial mouse chips. **c.** Heatmap of the expression of the top 3 marker genes for each cell type. **d.** Spatial and temporal distribution of mouse heart cells. **e.** Proportional changes of different cell types at four time points.

Figure 2 Cell-cell interactions of different cell types.

a. Cell interaction strength and interaction count at four time points. **b.** Cell interaction strength among cells at E20. The direction of the arrow indicates the direction of the signal, and the thickness of the line segment indicates the signal strength. **c.** Ingoing interaction strength of different cell types at four time points. **d.** Outgoing interaction strength of different cell types at four time points. **e.** Signal pathway strength of different cell types at four time points. The shade represents the relative strength of the interaction between the two cell types.

Figure 3 Classification of cell subtypes.

a-c. Subtype clustering of vCM, aCM, and FB. **d-f.** Expression of the top five marker genes in vCM, aCM, and FB. **g-i.** Enrichment of the top five GO functions in vCM, aCM, and FB.

Figure 4 Trajectory analysis and transcription factor analysis.

a. Trajectory analysis of three cell types (aCM, vCM, FB). The distribution of pseudo-time, cell types, time points, and cell states along the trajectory from left to right. **b.** Heatmap of gene expression changes in three cell states at branch node 1, and GO enrichment results of different clustered genes. The left cluster tree divides genes into

three groups according to the trend of gene expression. **c.** Transcription factor activity changes among three cell types (aCM, vCM, FB) at four time points. **d.** Changes in the activity of transcription factors targeting the gene *Igf2* in three different cell types over time. **e.** Correlation between *Plagl1* transcription factor activity and gene expression. The red dots represent target genes of *Plagl1*. f. Relative quantification of *Plagl1* at four time points using qPCR. g. Relative quantification of *Igf2* at four time points using qPCR.

Figure 5 Identification of left and right atria.

a-d. The spatial representation of HE sections, the actual regions of the left and right atria, and the clustering results of mouse heart at four time points. **e.** Accuracy of clustering results of the left and right atria relative to the actual distribution. **f.** Differential expression of genes comparing the left atrium to the right atrium at four time points. **g**. Intersection of highly variable genes in the left atrium at four time points. **h**. Intersection of highly variable genes in the right atrium at four time points.

Click here to access/download;Figure;Figure1.pdf

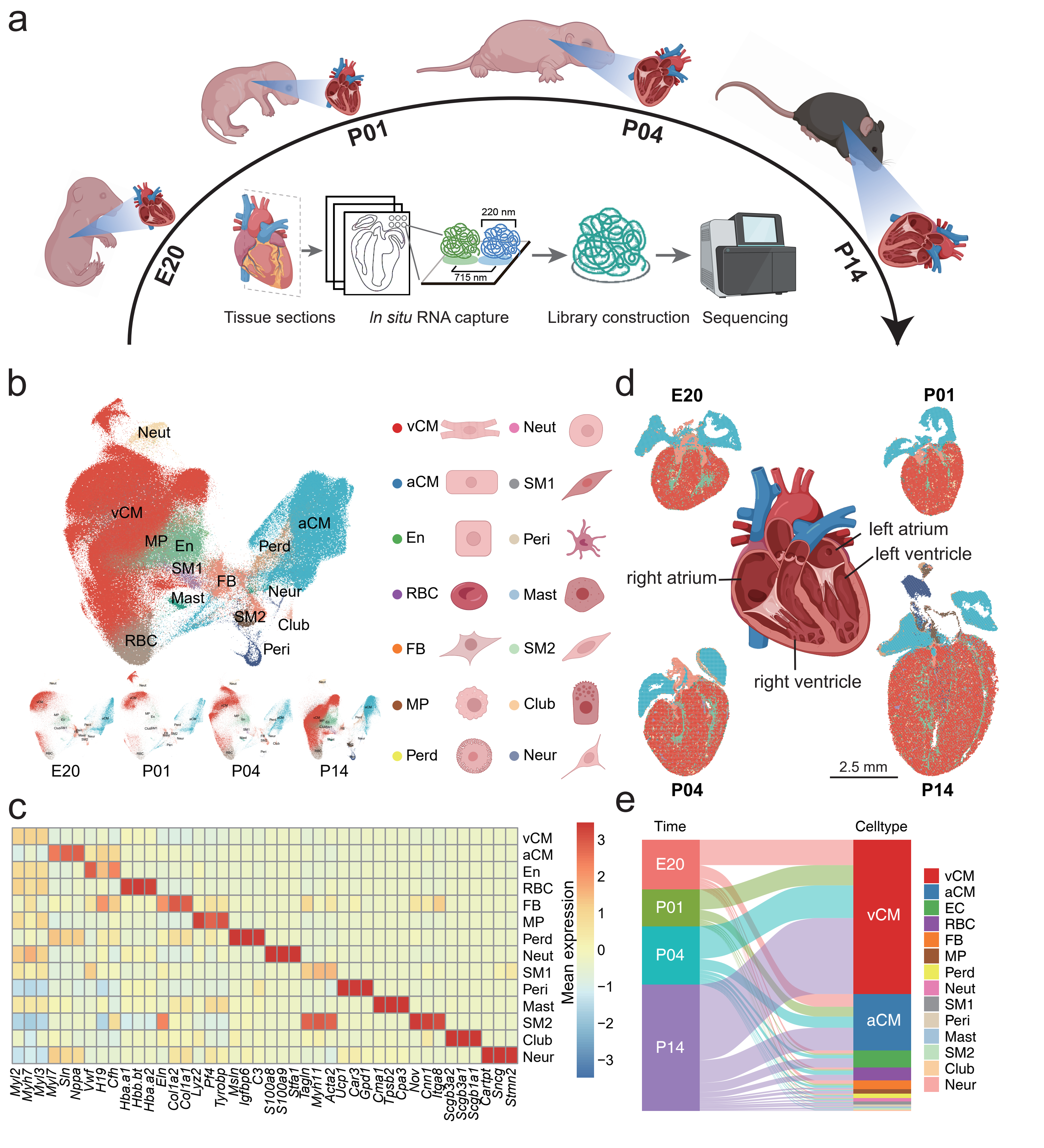

Figure 2

Click here to access/download;Figure;Figure2.pdf

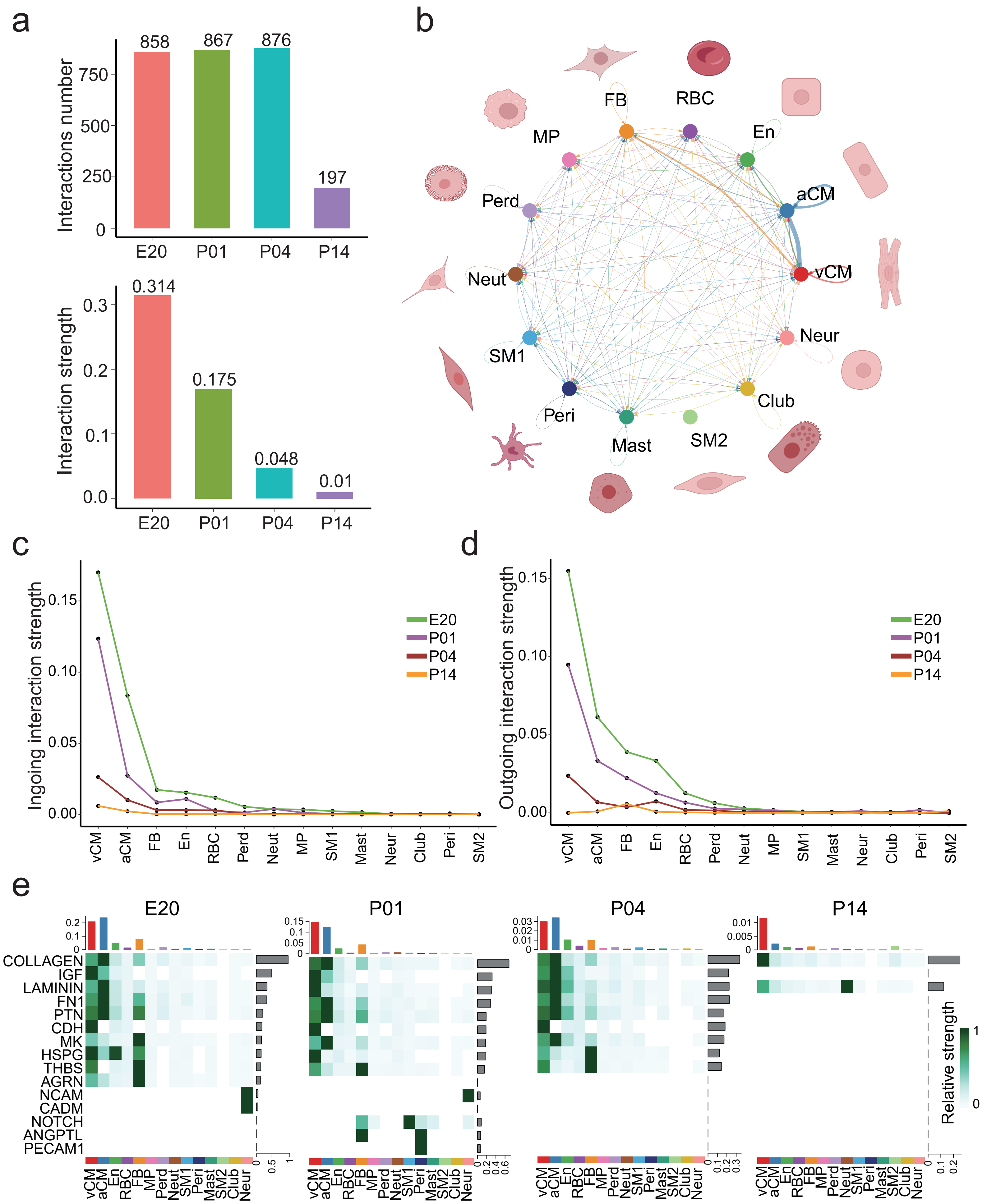

Figure 3

Click here to access/download;Figure;figure3.pdf

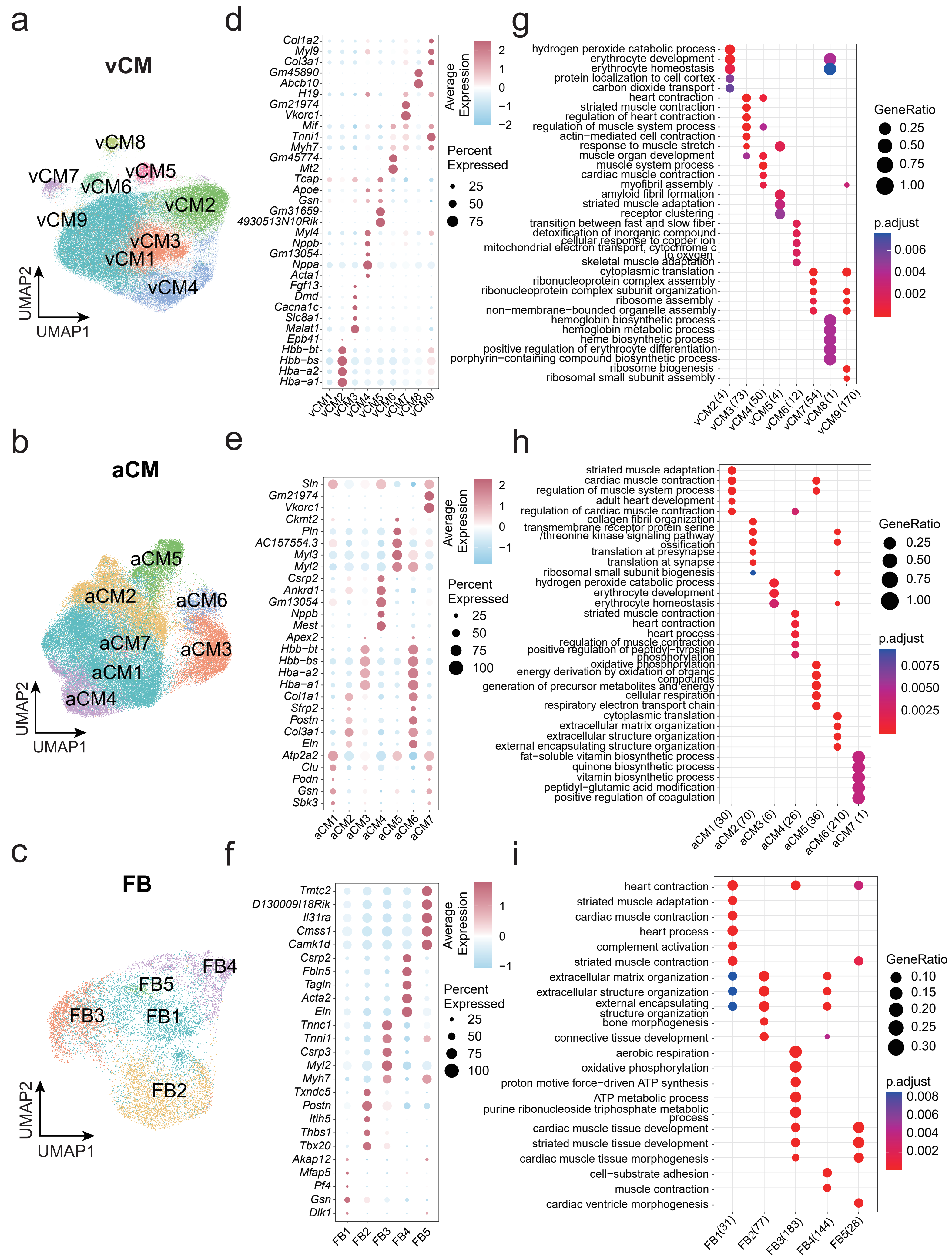

Figure 4

Click here to access/download;Figure;Figure4.pdf

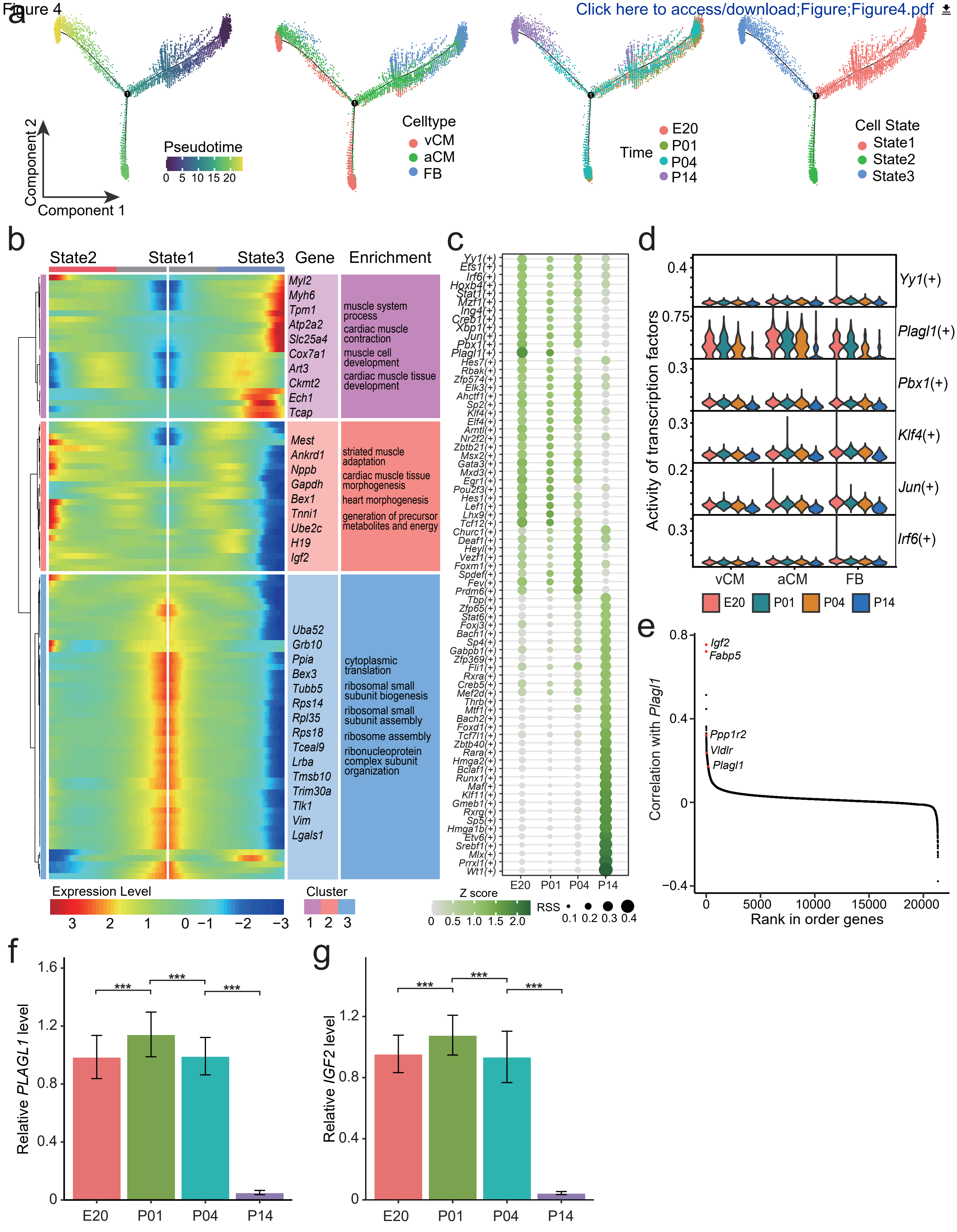

Figure 5

Click here to access/download;Figure;Figure5.pdf

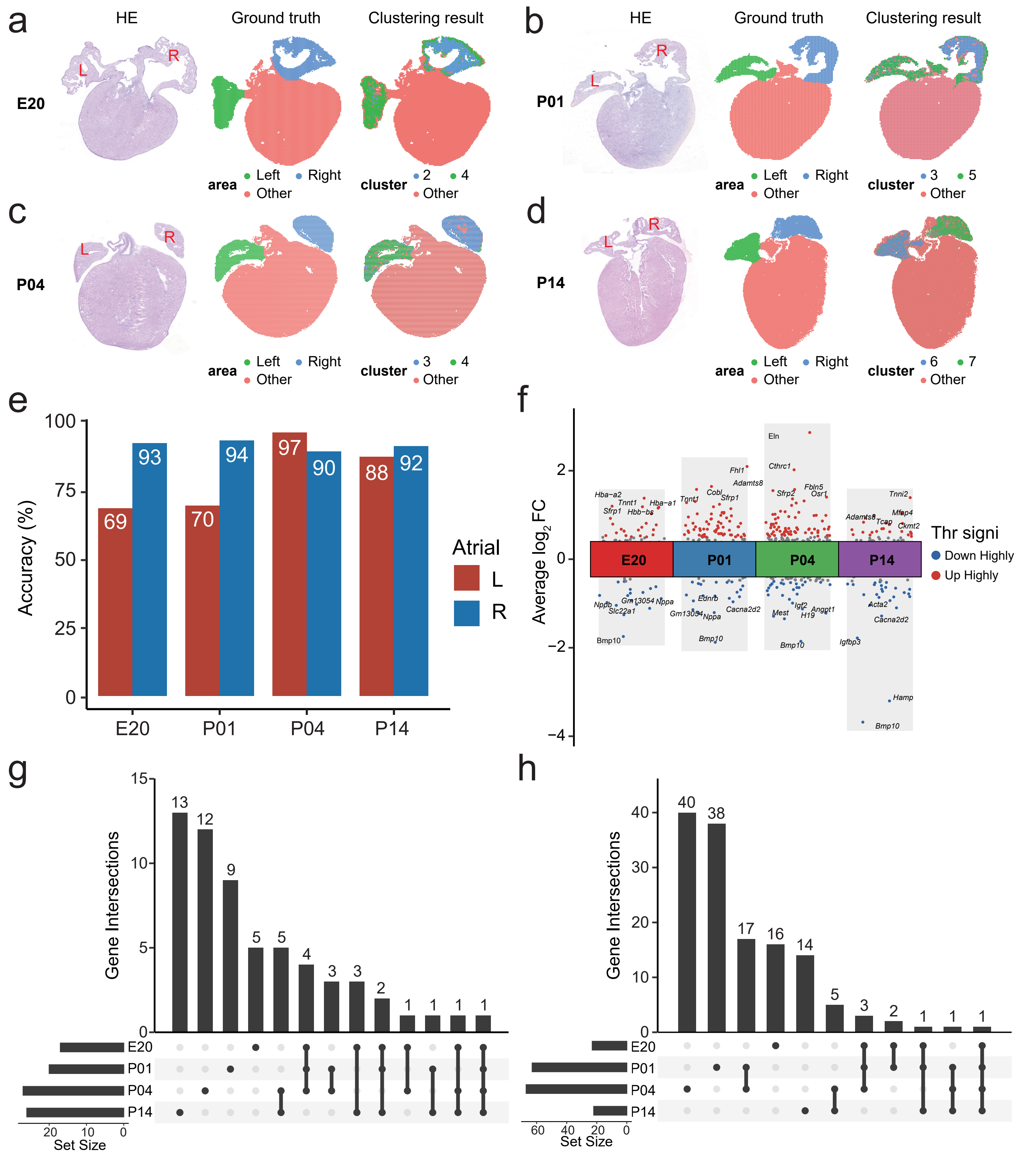

Click here to access/download
Supplementary Material
Supplementary data_V2.docx

Supplementary Tables

Click here to access/download
Supplementary Material
Supplementary Tables_V2.xlsx

Supplementary Figure 1

Click here to access/download
Supplementary Material
SF1.pdf

Supplementary Figure 2

Click here to access/download
Supplementary Material
SF2.pdf

Supplementary Figure 3

Click here to access/download
Supplementary Material
SF3.pdf

Supplementary Figure 4

Click here to access/download

Supplementary Material

SF4.pdf

Supplementary Figure 5

Click here to access/download
Supplementary Material
SF5.pdf

Supplementary Figure 6

Click here to access/download
Supplementary Material
SF6.pdf

Supplementary Figure 7

Click here to access/download
Supplementary Material
SF7.pdf

Supplementary Figure 8

Click here to access/download
Supplementary Material
SF8.pdf
