# Supplementary material for: Exploring the cellular and molecular basis of murine cardiac development through spatiotemporal transcriptome sequencing
Source: Gigascience. 2025 Feb 17;14:giaf012. doi: 10.1093/gigascience/giaf012 (PMC11831923; doi:10.1093/gigascience/giaf012)
Supplement: giaf012_Response_to_Reviewer_Comments_Original_Submission [file giaf012_response_to_reviewer_comments_original_submission.pdf]

Author's Response To Reviewer Comments

Reviewer1:
The manuscript presents a well-executed investigation into the dynamic changes occurring in the murine heart during four developmental stages, utilizing state-of-the-art Stereo-seq technology. The study reveals shifts in cardiac cell populations, such as the increase in certain cardiomyocyte subtypes and the decrease in others, and explores molecular mechanisms that play a role in the loss of regenerative capacity, heart asymmetry, and weakened intercellular interactions. The spatial transcriptomics data provided are of significant value, and the authors adeptly highlight the key findings. However, there are a number of major and minor issues that require attention to strengthen the manuscript.

Major Concerns:
1. Inclusion of Earlier Embryonic Stages:
While E20 is included, this stage is close to the postnatal period and may miss important transitions during earlier embryonic development. Incorporating time points such as E9.5, E12.5, E15.5, and E17.5 would provide a more complete picture of the heart's developmental trajectory and would greatly enhance the study's ability to capture critical phases of embryogenesis. The author may provide additional information using other public spatial transcriptomics data sets from earlier stages, such as those in Chen et al. 2020.
Response:
We appreciate your insightful suggestions and agree that incorporating transitions during earlier embryonic development would be beneficial. We have collected spatial transcriptomics data from early hearts at E9.5, E10.5, E11.5, and E12.5 stages as reported by Chen et al. (DOI: 10.1126/science.abg5159) and have conducted the following additional analyses:
(1) Since Chen et al. did not isolate cardiac cells specifically, we clustered the entire mouse embryo section data (Supplementary figure 6a), annotated cardiomyocytes using vCM markers (Myh12, Myl3, Myh7) and aCM markers (Myh14, Myl7, Nppa), and found that cardiac cells were located in clusters 8 and 16 (Supplementary figure 6b). However, cardiomyocytes in the mouse embryo are still undifferentiated at this stage, making it impossible to distinguish between aCM and vCM.
(2) We further counted the number of cardiomyocytes at four time points, which were 13 (E9.5), 66 (E10.5), 130 (E11.5), and 222 (E12.5), respectively, and demonstrated their spatial distribution (Supplementary figure 6c). Our results are largely consistent with the cardiomyocyte annotation by Chen et al.
(3) Due to the small number of early embryonic cardiomyocytes and the inability to differentiate specific cell subtypes, we did not include them in trajectory analysis. Instead, we presented key genes and transcription factors involved in cardiomyocyte differentiation (Supplementary figure 6d). We observed high expression of regeneration-related genes Igf2, H19, and transcription factor Plagl1, while transcription factors Wt1, Prrxl1, Srebf1 were lowly expressed. Genes related to myocardial contraction such as Tcap, Myh6, Atp2a2 showed an upward trend, consistent with the trends of cardiomyocyte differentiation before and after birth in mice (figure 4b-c).

2. Consistency in Cell Typing Across Figures:
Inconsistencies are noted in the cell subtyping presented in different figures. For instance, while Figure 2 provides detailed subtyping of ventricular and atrial cardiomyocytes (vCM, aCM) and fibroblasts (FB), this level of resolution is absent in Figures 3 and 4. Consistent cell subtyping across all figures is essential for accurately conveying the developmental trajectories and interactions of different cell types during heart development.
Response:
Thank you very much for the valuable suggestions from the reviewer! To make the logic of the article tighter, we have reorganized the order of Figures 2 and 3. The new figure sequence is as follows:
Figure 1: Spatial and temporal atlas of mouse heart development.
Figure 2: Cell-cell interactions of different cell types. We completed analyses of cell-cell interactions at four time points.
Figure 3: Classification of cell subtypes.
Figure 4: Trajectory analysis and transcription factor analysis.
Figure 5: Identification of left and right atria.

We first performed cell annotation on the cardiac spatial transcriptomics data (Fig. 1), then analyzed cell-cell interactions among major cell categories (Fig. 2). Noting that fibroblasts (FB), atrial cardiomyocytes (aCM), and ventricular cardiomyocytes (vCM) are the main cell types in the cardiac interaction network, we conducted subcategory annotations for these three cell types (Fig. 3). We then extracted 500 cells from each subtype to infer cell differentiation trajectories (Fig. 4). Finally, we identified specific genes for the left and right atria (Fig. 5).
We believe this new organization of figures is more logical and will assist readers in better understanding the content of each section and following the progress of the research more smoothly. Once again, thank you for your valuable feedback on the structure of the figures.

3. Experimental Validation of Key Findings:
The manuscript provides computational insights into myocardial regenerative loss and heart asymmetry, but these findings would be significantly strengthened by experimental validation. Validation of the computational predictions, especially the proposed key markers and transcription factors, would elevate the manuscript's overall impact.
Response：
Thank you very much for your valuable suggestions. We have validated the key genes potentially involved in the loss of cardiac regenerative capacity during heart development using qPCR, specifically focusing on Igf2 and Plagl1, a transcription factor that may regulate Igf2 during early heart development. Our observations show that Igf2 remains highly expressed until the heart loses its regenerative capacity, after which its expression level drops sharply. The expression trend of Plagl1 is very similar to that of Igf2, consistent with our spatial transcriptomics data. This further increases the reliability of our conclusions, which we have added to Figure 4f and Figure 4g.
Additionally, due to the characteristic of spatial location information (an increasing number of studies have begun to use spatial transcriptomics experiments as an alternative to traditional in situ hybridization experiments), we can visually observe the expression locations of left atrial and right atrial specific genes (Figure S7). Among the four marker genes that we have enriched, Pitx2 has been reported as a marker gene for the left atriuma, which is consistent with our conclusions. This consistency further increases the reliability of our results.
In summary, the integration of qPCR validation for critical genes like Igf2 and Plagl1, along with the spatial visualization of atrial-specific gene expression provided by spatial transcriptomics, significantly enhances the robustness and credibility of our findings regarding the mechanisms underlying the loss of cardiac regenerative capacity and atrial differentiation.

a. Logan, M., Pagán-Westphal, S. M., Smith, D. M., Paganessi, L. & Tabin, C. J. The transcription factor Pitx2 mediates situs-specific morphogenesis in response to left-right asymmetric signals. Cell 94, 307-317 (1998).

Minor Concerns:
1. Detail in Figure Legends:
The legends for several figures, particularly Figures 3 and 4, lack sufficient detail. For example, Figure 3 does not provide enough information regarding the parameters used in the cell-cell interaction analysis, and Figure 4 is missing specifics about the criteria used for trajectory analysis. Providing more comprehensive figure legends will make it easier for readers to interpret the data without having to repeatedly reference the main text.
Response：
We have rewritten the captions for Figures 1 through 5, paying special attention to Figures 3 and 4. In these captions, we have included more detailed explanations, such as the meanings of the axes and legends, parameters used, significance markers, abbreviations, and symbols. Furthermore, we checked and ensured that consistent terminology and formatting were used across all captions to better assist readers in understanding the data. We believe these improvements will make the captions clearer and easier for readers to comprehend.

2. Figure Readability:
The text in multiple figures is difficult to read due to low resolution and small font sizes. Enhancing the resolution and adjusting the font size and formatting would improve the readability of the figures and

ensure that labels and annotations are clear.
Response：
We greatly appreciate your valuable suggestions. In response, we have saved the figures in high-resolution PDF format and provided PNG files with a resolution of 500 dpi. Moreover, we have adjusted the font sizes and formatting to enhance readability. We carefully reviewed all text within the figures, including axis labels, legends, and titles, to ensure they are clear and legible. We thank you for your attention to these details, as it has significantly improved the overall quality and clarity of the data.

3. Clarification of Figure 5's Contribution:
Figure 5 appears to add little to the overall analysis and could benefit from further explanation. It would be helpful to include a more intuitive visualization that highlights key spatial transcriptomic insights, providing a clearer understanding of the data. Improving the spatial representation would make this figure more informative and relevant.
Response：
We have added the results of unsupervised clustering for the left and right atria. This addition is intended to better illustrate the differences between the clustering outcomes and the actual conditions of the left and right atria. We believe this will enable readers to visually observe the advantages of spatial transcriptomics data with spatial location information in annotating the left and right atria.

Reviewer2:
Kang et al. utilized the highest-resolution spatial transcriptomics sequencing technology (Stereo-Seq) to map the spatial transcriptome atlas of the mouse heart across four developmental stages (E20, P01, P4, P14). This study revealed changes in cells and genes within the heart and, based on high-resolution spatial data, further subclustered vCMs, aCMs, and FBs. Subsequently, it explored changes in interactions among heart cells, possible reasons for the loss of cardiac regeneration ability, and cellular asymmetry, highlighting the importance of spatial location information. Overall, the authors ingeniously emphasized the critical role of position-specific information unique to spatial transcriptomics, providing a valuable data resource for cardiac spatial transcriptomics. However, before publication, further modifications are needed.

Major suggestions:
1. Figure 2 describes the sub-cellular types of vCM, aCM, and FB, but Figure 3 discusses the cell-cell interactions of all cardiac cells. Figure 4 then utilizes the results of sub-cellular clustering obtained from Figure 2 for pseudo-time analysis. The authors should adjust the order of Figure 2 and Figure 3 to make the article more logical.
Response:
Thank you very much for the valuable suggestions from the reviewer! To make the logic of the article tighter, we have reorganized the order of Figures 2 and 3. The new figure sequence is as follows:
Figure 1: Spatial and temporal atlas of mouse heart development.
Figure 2: Cell-cell interactions of different cell types. We completed analyses of cell-cell interactions at four time points.
Figure 3: Classification of cell subtypes.
Figure 4: Trajectory analysis and transcription factor analysis.
Figure 5: Identification of left and right atria.
We first performed cell annotation on the cardiac spatial transcriptomics data (Fig. 1), then analyzed cell-cell interactions among major cell categories (Fig. 2). Noting that fibroblasts (FB), atrial cardiomyocytes (aCM), and ventricular cardiomyocytes (vCM) are the main cell types in the cardiac interaction network, we conducted subcategory annotations for these three cell types (Fig. 3). We then extracted 500 cells from each subtype to infer cell differentiation trajectories (Fig. 4). Finally, we identified specific genes for the left and right atria (Fig. 5).
We believe this new organization of figures is more logical and will assist readers in better understanding the content of each section and following the progress of the research more smoothly. Once again, thank you for your valuable feedback on the structure of the figures.

2. The description of the specific source of the results in Figure 5e is not very clear. The authors need to provide a clearer description of how this result was calculated, such as adding some figures to help readers understand more intuitively.
Response:

Thank you very much for the suggestion. We have added the results of unsupervised clustering for the left and right atria in Figure 5a-d. This allows readers to visually observe the differences in annotation of the left and right atria with and without spatial location information, thereby providing a more intuitive understanding of the meaning of Figure 5e. Additionally, we have detailed in Supplementary Table 9 how we calculated the annotation accuracy presented in Figure 5e. We believe this will significantly enhance readers' comprehension of Figure 5e.

3. The manuscript identified potential key genes and transcription factors associated with the loss of myocardial regenerative capacity. These findings require experimental validation to enhance their reliability. The authors should at least demonstrate through qPCR that genes such as Igf2 and transcription factors like PLAGL1 indeed significantly decrease at P14.
Response:
Thank you very much for your valuable suggestions. We have validated the key genes potentially involved in the loss of cardiac regenerative capacity during heart development using qPCR, specifically focusing on Igf2 and Plagl1, a transcription factor that may regulate Igf2 during early heart development. Our observations show that Igf2 remains highly expressed until the heart loses its regenerative capacity, after which its expression level drops sharply. The expression trend of Plagl1 is very similar to that of Igf2, consistent with our spatial transcriptomics data. This further increases the reliability of our conclusions, which we have added to Figure 4f and Figure 4g.

Minor suggestions:
1. Several figures are illegible due to low resolution or color scheme issues. The authors need to enhance the resolution of the images to improve readability.
Response:
We greatly appreciate your valuable suggestions. In response, we have saved the figures in high-resolution PDF format and provided PNG files with a resolution of 500 dpi. Moreover, we have adjusted the font sizes and formatting to enhance readability. We carefully reviewed all text within the figures, including axis labels, legends, and titles, to ensure they are clear and legible. We thank you for your attention to these details, as it has significantly improved the overall quality and clarity of the data.

2. I suggest that the author carefully review the language of the article. When assessing the clarity of your text, note that the picture you have in your mind and the picture evoked by your writing in your readers' minds should be identical. If you are curious about whether your revised text is clear, try this exercise with a respected colleague who is not familiar with the details of this study. Note that clear text is a prerequisite for any fulsome scientific assessment.
Response:
Thank you very much for your suggestion. We understand that clear text is crucial for any comprehensive scientific assessment, and we have carefully revised the language throughout the manuscript. We believe the text is now sufficiently clear for others to understand.

3. I suggest that the author check the text to avoid basic grammatical errors, such as "(E20, POland P04)" in line 21.
Response:
We have carefully reviewed the text and corrected any grammatical errors. We have ensured grammatical accuracy and clarity to improve the quality of the article. If there are other specific suggestions for changes, please feel free to point them out.

4. Add a separator for the numbers over 1,000. Check all numbers including those in the tables/figures, such as "This database 5 comprises 2021 validated molecular interactions,…". Please check to see if there are other places with similar problems.
Response:
We have carefully reviewed all numbers in the document and have added appropriate separators to all numbers greater than 1000 as suggested by you. We have thoroughly checked the numbers in the document to ensure that we have not missed any similar problems. Thank you very much for your attention to detail, which will help improve the quality and readability of your manuscript.

5. The following sentence requires a space between the number and the unit: "This database was

constructed using the 2022 SCENIC+ motif collection, defining the search space as 500bp upstream of the transcription start site (TSS) and 100bp downstream around the TSS of the gene where the motif is scored."

Response:

Thank the reviewers for their careful review and valuable comments! We will follow your suggestion to add appropriate Spaces between numbers and units to improve the normativity and readability of the text. We did a careful review of the document to ensure that all similar formatting issues had been corrected.
