# Supplementary material for: Exploring the cellular and molecular basis of murine cardiac development through spatiotemporal transcriptome sequencing
Source: Gigascience. 2025 Feb 17;14:giaf012. doi: 10.1093/gigascience/giaf012 (PMC11831923; doi:10.1093/gigascience/giaf012)
Supplement: giaf012_Reviewer_1_Report_Original_Submission — Joseph C. Wu -- 10/13/2024 [file giaf012_reviewer_1_report_original_submission.pdf]

Reviewer Report

Title: Exploring the Cellular and Molecular Basis of Murine Cardiac Development through Spatiotemporal Transcriptome Sequencing

Version: Original Submission **Date:** 10/13/2024

Reviewer name: Joseph C. Wu

Reviewer Comments to Author:

The manuscript presents a well-executed investigation into the dynamic changes occurring in the murine heart during four developmental stages, utilizing state-of-the-art Stereo-seq technology. The study reveals shifts in cardiac cell populations, such as the increase in certain cardiomyocyte subtypes and the decrease in others, and explores molecular mechanisms that play a role in the loss of regenerative capacity, heart asymmetry, and weakened intercellular interactions. The spatial transcriptomics data provided are of significant value, and the authors adeptly highlight the key findings. However, there are a number of major and minor issues that require attention to strengthen the manuscript.

Major Concerns:

1. Inclusion of Earlier Embryonic Stages:

While E20 is included, this stage is close to the postnatal period and may miss important transitions during earlier embryonic development. Incorporating time points such as E9.5, E12.5, E15.5, and E17.5 would provide a more complete picture of the heart's developmental trajectory and would greatly enhance the study's ability to capture critical phases of embryogenesis. The author may provide additional information using other public spatial transcriptomics data sets from earlier stages, such as those in Chen et al. 2020.

2. Consistency in Cell Typing Across Figures:

Inconsistencies are noted in the cell subtyping presented in different figures. For instance, while Figure 2 provides detailed subtyping of ventricular and atrial cardiomyocytes (vCM, aCM) and fibroblasts (FB), this level of resolution is absent in Figures 3 and 4. Consistent cell subtyping across all figures is essential for accurately conveying the developmental trajectories and interactions of different cell types during heart development.

3. Experimental Validation of Key Findings:

The manuscript provides computational insights into myocardial regenerative loss and heart asymmetry, but these findings would be significantly strengthened by experimental validation. Validation of the computational predictions, especially the proposed key markers and transcription factors, would elevate the manuscript's overall impact.

Minor Concerns:

1. Detail in Figure Legends:

The legends for several figures, particularly Figures 3 and 4, lack sufficient detail. For example, Figure 3 does not provide enough information regarding the parameters used in the cell-cell interaction analysis, and Figure 4 is missing specifics about the criteria used for trajectory analysis. Providing more comprehensive figure legends will make it easier for readers to interpret the data without having to

repeatedly reference the main text.
2. Figure Readability:
The text in multiple figures is difficult to read due to low resolution and small font sizes. Enhancing the resolution and adjusting the font size and formatting would improve the readability of the figures and ensure that labels and annotations are clear.
3. Clarification of Figure 5's Contribution:
Figure 5 appears to add little to the overall analysis and could benefit from further explanation. It would be helpful to include a more intuitive visualization that highlights key spatial transcriptomic insights, providing a clearer understanding of the data. Improving the spatial representation would make this figure more informative and relevant.
Conclusion:
Overall, this study addresses a significant area in cardiac development and presents some intriguing findings through spatial transcriptomics. However, the manuscript requires substantial additional work to meet the high standards expected in this field. Specifically, incorporating earlier developmental stages, adding experimental validation, and addressing the presentation issues outlined above are critical to making the study more comprehensive and robust. Without these improvements, the current version may not fully meet the expectations for publication in a journal of this caliber.

Methods

Are the methods appropriate to the aims of the study, are they well described, and are necessary controls included? Choose an item.

Conclusions

Are the conclusions adequately supported by the data shown? Choose an item.

Reporting Standards

Does the manuscript adhere to the journal's guidelines on minimum standards of reporting? Choose an item.

Choose an item.

Statistics

Are you able to assess all statistics in the manuscript, including the appropriateness of statistical tests used? Choose an item.

Quality of Written English

Please indicate the quality of language in the manuscript: Choose an item.

Declaration of Competing Interests

Please complete a declaration of competing interests, considering the following questions:

- Have you in the past five years received reimbursements, fees, funding, or salary from an organisation that may in any way gain or lose financially from the publication of this manuscript, either now or in the future?
- Do you hold any stocks or shares in an organisation that may in any way gain or lose financially from the publication of this manuscript, either now or in the future?
- Do you hold or are you currently applying for any patents relating to the content of the manuscript?
- Have you received reimbursements, fees, funding, or salary from an organization that holds or has applied for patents relating to the content of the manuscript?
- Do you have any other financial competing interests?
- Do you have any non-financial competing interests in relation to this paper?

If you can answer no to all of the above, write 'I declare that I have no competing interests' below. If your reply is yes to any, please give details below.

I declare that I have no competing interests

I agree to the open peer review policy of the journal. I understand that my name will be included on my report to the authors and, if the manuscript is accepted for publication, my named report including any attachments I upload will be posted on the website along with the authors' responses. I agree for my report to be made available under an Open Access Creative Commons CC-BY license (http://creativecommons.org/licenses/by/4.0/). I understand that any comments which I do not wish to be included in my named report can be included as confidential comments to the editors, which will not be published.

Choose an item.

To further support our reviewers, we have joined with Publons, where you can gain additional credit to further highlight your hard work (see: https://publons.com/journal/530/gigascience). On publication of this paper, your review will be automatically added to Publons, you can then choose whether or not to claim your Publons credit. I understand this statement.

Yes Choose an item.
