# Supplementary material for: Exploring the cellular and molecular basis of murine cardiac development through spatiotemporal transcriptome sequencing
Source: Gigascience. 2025 Feb 17;14:giaf012. doi: 10.1093/gigascience/giaf012 (PMC11831923; doi:10.1093/gigascience/giaf012)
Supplement: giaf012_Reviewer_2_Report_Original_Submission — Yuliang Feng -- 11/27/2024 [file giaf012_reviewer_2_report_original_submission.pdf]

Reviewer Report

Title: Exploring the Cellular and Molecular Basis of Murine Cardiac Development through Spatiotemporal Transcriptome Sequencing

Version: Original Submission **Date:** 11/27/2024

Reviewer name: Yuliang Feng

Reviewer Comments to Author:

Kang et al. utilized the highest-resolution spatial transcriptomics sequencing technology (Stereo-Seq) to map the spatial transcriptome atlas of the mouse heart across four developmental stages (E20, P01, P4, P14). This study revealed changes in cells and genes within the heart and, based on high-resolution spatial data, further subclustered vCMs, aCMs, and FBs. Subsequently, it explored changes in interactions among heart cells, possible reasons for the loss of cardiac regeneration ability, and cellular asymmetry, highlighting the importance of spatial location information. Overall, the authors ingeniously emphasized the critical role of position-specific information unique to spatial transcriptomics, providing a valuable data resource for cardiac spatial transcriptomics. However, before publication, further modifications are needed.

Major suggestions:

a. Figure 2 describes the sub-cellular types of vCM, aCM, and FB, but Figure 3 discusses the cell-cell interactions of all cardiac cells. Figure 4 then utilizes the results of sub-cellular clustering obtained from Figure 2 for pseudo-time analysis. The authors should adjust the order of Figure 2 and Figure 3 to make the article more logical.

b. The description of the specific source of the results in Figure 5e is not very clear. The authors need to provide a clearer description of how this result was calculated, such as adding some figures to help readers understand more intuitively.

c. The manuscript identified potential key genes and transcription factors associated with the loss of myocardial regenerative capacity. These findings require experimental validation to enhance their reliability. The authors should at least demonstrate through qPCR that genes such as Igf2 and transcription factors like PLAGL1 indeed significantly decrease at P14.

Minor suggestions:

a. Several figures are illegible due to low resolution or color scheme issues. The authors need to enhance the resolution of the images to improve readability.

b. I suggest that the author carefully review the language of the article. When assessing the clarity of your text, note that the picture you have in your mind and the picture evoked by your writing in your readers' minds should be identical. If you are curious about whether your revised text is clear, try this exercise with a respected colleague who is not familiar with the details of this study. Note that clear text is a prerequisite for any fulsome scientific assessment.

c. I suggest that the author check the text to avoid basic grammatical errors, such as "(E20, POland P04)" in line 21.

d. Add a separator for the numbers over 1,000. Check all numbers including those in the tables/figures,

such as "This database 5 comprises 2021 validated molecular interactions,…". Please check to see if there are other places with similar problems.
e. The following sentence requires a space between the number and the unit: "This database was constructed using the 2022 SCENIC+ motif collection, defining the search space as 500bp upstream of the transcription start site (TSS) and 100bp downstream around the TSS of the gene where the motif is scored."

Methods

Are the methods appropriate to the aims of the study, are they well described, and are necessary controls included? Choose an item.

Conclusions

Are the conclusions adequately supported by the data shown? Choose an item.

Reporting Standards

Does the manuscript adhere to the journal's guidelines on minimum standards of reporting? Choose an item.

Choose an item.

Statistics

Are you able to assess all statistics in the manuscript, including the appropriateness of statistical tests used? Choose an item.

Quality of Written English

Please indicate the quality of language in the manuscript: Choose an item.

Declaration of Competing Interests

Please complete a declaration of competing interests, considering the following questions:

- Have you in the past five years received reimbursements, fees, funding, or salary from an organisation that may in any way gain or lose financially from the publication of this manuscript, either now or in the future?
- Do you hold any stocks or shares in an organisation that may in any way gain or lose financially from the publication of this manuscript, either now or in the future?
- Do you hold or are you currently applying for any patents relating to the content of the manuscript?

- Have you received reimbursements, fees, funding, or salary from an organization that holds or has applied for patents relating to the content of the manuscript?
- Do you have any other financial competing interests?
- Do you have any non-financial competing interests in relation to this paper?

If you can answer no to all of the above, write 'I declare that I have no competing interests' below. If your reply is yes to any, please give details below.

I declare that I have no competing interests' below

I agree to the open peer review policy of the journal. I understand that my name will be included on my report to the authors and, if the manuscript is accepted for publication, my named report including any attachments I upload will be posted on the website along with the authors' responses. I agree for my report to be made available under an Open Access Creative Commons CC-BY license (http://creativecommons.org/licenses/by/4.0/). I understand that any comments which I do not wish to be included in my named report can be included as confidential comments to the editors, which will not be published.

Choose an item.

To further support our reviewers, we have joined with Publons, where you can gain additional credit to further highlight your hard work (see: https://publons.com/journal/530/gigascience). On publication of this paper, your review will be automatically added to Publons, you can then choose whether or not to claim your Publons credit. I understand this statement.

Yes Choose an item.
